# Replication-Coupled DNA-Protein Crosslink Repair by SPRTN and the Proteasome in *Xenopus* Egg Extracts

**DOI:** 10.1016/j.molcel.2018.11.024

**Published:** 2019-02-07

**Authors:** Nicolai B. Larsen, Alan O. Gao, Justin L. Sparks, Irene Gallina, R. Alex Wu, Matthias Mann, Markus Räschle, Johannes C. Walter, Julien P. Duxin

**Affiliations:** 1 Faculty of Health and Medical Sciences, Novo Nordisk Foundation Center for Protein Research, University of Copenhagen, DK-2200 Copenhagen, Denmark; 2 Department of Biological Chemistry and Molecular Pharmacology, Harvard Medical School, Boston, MA 02115, USA; 3 Department of Proteomics and Signal Transduction, Max Planck Institute of Biochemistry, 82152 Martinsried, Germany; 4 Department of Molecular Biotechnology and Systems Biology, Technical University of Kaiserslautern, 67653 Kaiserslautern, Germany; 5 Howard Hughes Medical Institute, Department of Biological Chemistry and Molecular Pharmacology, Harvard Medical School, Boston, MA 02115, USA

**Keywords:** DNA-portein crosslink (DPC), DNA replication, DNA repair, Ubiquitin, Proteasome, SPRTN, TRAIP, translesion synthesis (TLS)

## Abstract

DNA-protein crosslinks (DPCs) are bulky lesions that interfere with DNA metabolism and therefore threaten genomic integrity. Recent studies implicate the metalloprotease SPRTN in S phase removal of DPCs, but how SPRTN is targeted to DPCs during DNA replication is unknown. Using *Xenopus* egg extracts that recapitulate replication-coupled DPC proteolysis, we show that DPCs can be degraded by SPRTN or the proteasome, which act as independent DPC proteases. Proteasome recruitment requires DPC polyubiquitylation, which is partially dependent on the ubiquitin ligase activity of TRAIP. In contrast, SPRTN-mediated DPC degradation does not require DPC polyubiquitylation but instead depends on nascent strand extension to within a few nucleotides of the lesion, implying that polymerase stalling at the DPC activates SPRTN on both leading and lagging strand templates. Our results demonstrate that SPRTN and proteasome activities are coupled to DNA replication by distinct mechanisms that promote replication across immovable protein barriers.

## Highlights

• The proteasome and SPRTN can each degrade DPCs during DNA replication
• Efficient DPC ubiquitylation and proteasome targeting requires TRAIP
• SPRTN can degrade DPCs that are not ubiquitylated
• SPRTN-mediated DPC degradation is a post-replicative process

## Graphical Abstract

**Figure:**
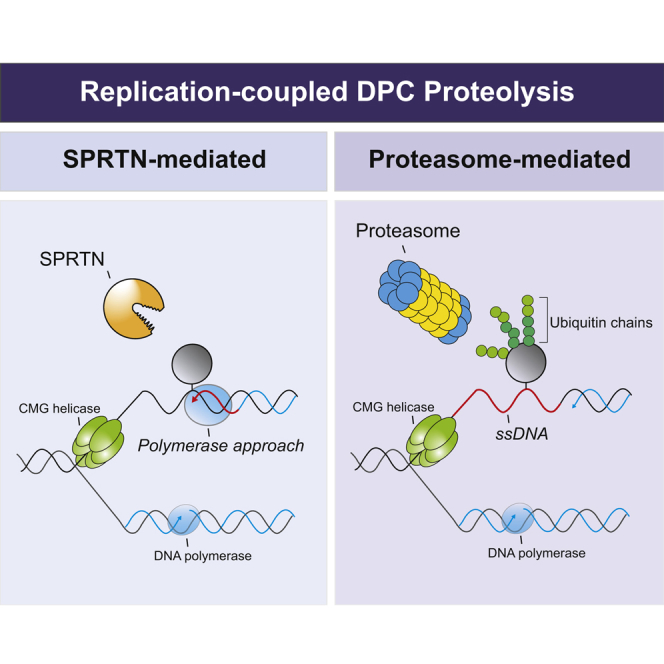

## Teaser

DNA-protein crosslinks (DPCs) pose major threats to genomic integrity. Using a cell-free system, Larsen et al. show that SPRTN and the proteasome operate as independent replication-coupled DPC proteases. The existence of two DPC proteases with distinct modes of action facilitates the degradation of chemically diverse DPCs.

## Introduction

Vertebrate chromatin is composed of myriad proteins that perform a multitude of functions. Sometimes, these proteins are covalently trapped on DNA, yielding DNA-protein crosslinks (DPCs) (Barker et al., 2005, Ide et al., 2011, Tretyakova et al., 2015). While DPCs generated by most crosslinking agents (e.g., formaldehyde, cisplatin-based chemotherapeutics) link proteins to uninterrupted duplex DNA (type I DPCs), abortive reactions by DNA repair enzymes such as topoisomerase I and II form DPCs that are flanked on one side by a single-stranded (type II DPCs) or double-stranded DNA break (type III DPCs), respectively (Ide et al., 2011). Left unrepaired, DPCs stall or inhibit DNA replication and transcription and thereby threaten genomic integrity (Chválová et al., 2007, Duxin et al., 2014, Kuo et al., 2007, Nakano et al., 2012, Novakova et al., 2003).

Given the frequency and cytotoxicity of DPC lesions, cells have evolved pathways to promote their removal. While nucleotide excision repair and homologous recombination have been linked to DPC repair (Ide et al., 2011), recent experiments in yeast identified the metalloprotease Wss1 as a dedicated DPC-repair factor (Stingele et al., 2014). Wss1 removes DPCs from the genome by degrading crosslinked proteins (Balakirev et al., 2015, Stingele et al., 2014). In contemporaneous experiments, we recapitulated replication-coupled DPC proteolysis in *Xenopus* egg extracts (Duxin et al., 2014). In this mechanism, a type I DPC encountered by the replisome is degraded to a short peptide adduct. Degradation of the DPC facilitates replisome bypass and DNA synthesis across the lesion by the translesion synthesis (TLS) polymerase complex Rev1-Polζ (Duxin et al., 2014). In this manner, the replisome simultaneously overcomes DPCs and clears them from the genome. Collectively, the experiments in yeast and in *Xenopus* established the existence of a dedicated, S-phase proteolytic DPC-repair pathway, although the protease acting in vertebrates remained elusive at the time.

Studies in mammalian cells suggest that the proteasome also participates in DPC removal (Baker et al., 2007, Desai et al., 1997, Lin et al., 2008, Mao et al., 2001, Quiñones et al., 2015, Zecevic et al., 2010). Proteasome inhibition prevents the removal of different types of DPCs, including trapped topoisomerases and DNA Polβ (Desai et al., 1997, Lin et al., 2008, Mao et al., 2001, Quiñones et al., 2015), and sensitizes cells to formaldehyde treatment (Ortega-Atienza et al., 2015). In addition, DPC polyubiquitylation was reported in the case of covalent topoisomerase I (Desai et al., 1997). However, polyubiquitylation of the more abundant type I DPCs could not be observed (Nakano et al., 2009), and it is therefore unclear whether DPCs are generally targeted by the proteasome. In *Xenopus* egg extracts, inhibition of the proteasome on its own does not significantly stabilize type I DPCs during DNA replication (Duxin et al., 2014). Therefore, whether the proteasome acts on different types of DPCs and whether this process operates during DNA replication remain open questions.

Recently, the metalloprotease SPARTAN (SPRTN) has been implicated in DPC degradation in higher eukaryotes. SPRTN shares homology with the yeast DPC protease Wss1 and is proposed to be functionally similar (Stingele et al., 2015, Vaz et al., 2017). In humans, mutations in SPRTN that compromise its protease activity cause Ruijs-Aalfs syndrome (RJALS), which is characterized by genomic instability, premature aging, and hepatocellular carcinoma (Lessel et al., 2014). In mice, loss of SPRTN is embryonically lethal, and conditional inactivation of SPRTN in murine embryonic fibroblasts (MEFs) blocks cell proliferation (Maskey et al., 2014). Although SPRTN was initially characterized as a regulator of TLS (Centore et al., 2012, Davis et al., 2012, Mosbech et al., 2012), several recent reports suggest that its essential role in genome maintenance involves DPC proteolysis (Lopez-Mosqueda et al., 2016, Maskey et al., 2017, Mórocz et al., 2017, Stingele et al., 2016, Vaz et al., 2016). SPRTN is predominantly expressed in S phase and associates with replisome components (Ghosal et al., 2012, Kim et al., 2013, Mosbech et al., 2012, Vaz et al., 2016). In the absence of SPRTN, cells accumulate DPCs and exhibit impaired replication fork progression (Lessel et al., 2014, Mórocz et al., 2017, Vaz et al., 2016). The data suggest that DPCs readily form *in vivo* and that cells rely on SPRTN-dependent DPC removal to suppress genome instability, cancer, and aging.

SPRTN proteolytic activity is regulated via different mechanisms. First, SPRTN undergoes monoubiquitylation (Mosbech et al., 2012), which prevents its recruitment to chromatin (Stingele et al., 2016). DPC induction triggers SPRTN deubiquitylation by an unknown ubiquitin protease, allowing SPRTN to localize to chromatin and initiate DPC degradation (Stingele et al., 2016). Once SPRTN is recruited to chromatin, DNA binding stimulates its protease activity (Lopez-Mosqueda et al., 2016, Mórocz et al., 2017, Stingele et al., 2016, Vaz et al., 2016), and evidence indicates that SPRTN is uniquely activated by single-stranded DNA (ssDNA) (Stingele et al., 2016). SPRTN also degrades itself, which may switch off its proteolytic function when repair is complete (Stingele et al., 2016, Vaz et al., 2016). Although these findings suggest that SPRTN activity is subject to elaborate regulation, they do not explain how SPRTN is specifically directed to DPCs during DNA replication or how non-specific replisome destruction is avoided.

We investigated the molecular mechanisms that link DPC degradation to DNA replication. Here, we report that SPRTN and the proteasome function as two replication-coupled DPC proteases. Proteasome recruitment to DPCs depends on replication-dependent DPC ubiquitylation. In contrast, SPRTN-mediated DPC degradation can occur in the absence of DPC ubiquitylation, but instead requires the extension of a nascent strand to the DPC. Our results reveal how SPRTN and proteasome activities are targeted to DPCs to facilitate replication across these covalent protein barriers.

## Results

### DPCs Are Ubiquitylated and Degraded during DNA Replication

**Figure 1:**
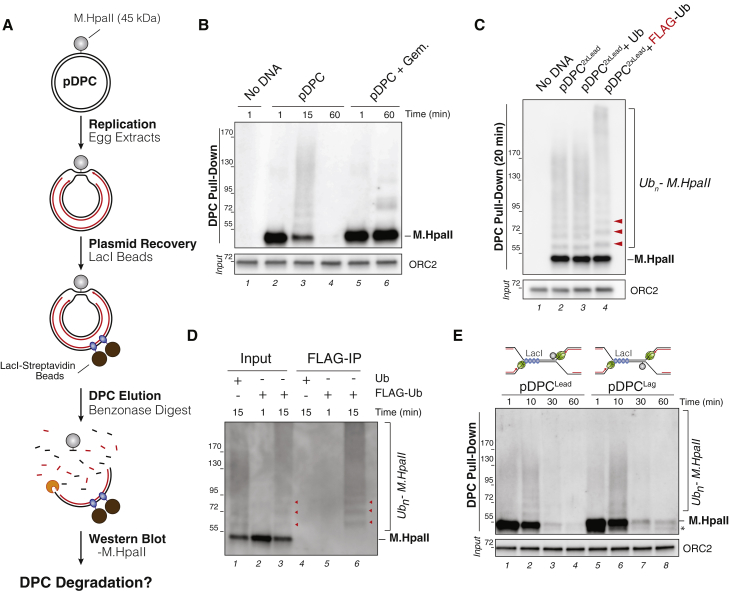
Replication-Coupled Ubiquitylation and Degradation of a DPC (A) Schematic of the DPC recovery assay. (B) pDPC was replicated in egg extracts. Geminin (+ Gem.) was added where indicated to block DNA replication. DPCs were recovered as illustrated in (A) at the indicated time points, and DPCs were blotted with a M.HpaII antibody. Input samples were blotted with an origin recognition complex subunit 2 (ORC2) antibody. (C) pDPC^2xLead^, a plasmid containing two DPCs (one on each leading strand; see Figure 2A), was replicated in egg extracts supplemented with free ubiquitin (Ub) or FLAG-ubiquitin. At 20 min, DPCs were recovered and blotted as in (B). Red arrowheads indicate the mobility shift induced by FLAG-ubiquitin. (D) pDPC^2xLead^ was replicated in egg extracts supplemented with free ubiquitin (Ub) or FLAG-ubiquitin. At the indicated time point, DPCs were recovered as in (B) (Input) and immunoprecipitated with anti-FLAG-resin (FLAG-IP). Ubiquitylated DPCs were detected with M.HpaII antibody. Red arrowheads indicate the location of mono-, di-, and tri-ubiquitylated M.HpaII. (E) pDPC^Lead^ or pDPC^Lag^ was replicated in egg extract in the presence of LacI to ensure that a single replication fork encounters the DPC (Duxin et al., 2014). Recovered DPCs were blotted as in (B). The asterisk indicates residual uncrosslinked M.HpaII.

To investigate DPC repair, the 45-kDa DNA methyltransferase HpaII (M.HpaII) was trapped at a fluorinated sequence on a plasmid to generate a type I DPC (Chen et al., 1991). During replication of the resulting plasmid (pDPC) in *Xenopus* egg extracts, converging forks transiently stall at the DPC, after which daughter plasmid molecules are resolved (Figure S1A; Duxin et al., 2014). The daughter molecule containing the DPC initially migrates as an open circular (OC) species and is then gradually converted to a supercoiled (SC) repair product through proteolysis of the DPC and TLS across the resulting peptide adduct (Figures S1A and S1B). To monitor the integrity of the DPC, we pulled down the plasmid under stringent conditions, digested the DNA, and analyzed M.HpaII via immunoblotting (Figure 1A). At 15 min, when replication was under way (Figure S1B), covalently attached M.HpaII migrated as a ladder of slow mobility species that subsequently disappeared (Figure 1B, lanes 2–4). The addition of FLAG-ubiquitin to the extract shifted the mobility of the M.HpaII species (Figure 1C, lane 4), indicating that they correspond to ubiquitylated M.HpaII. These slow mobility M.HpaII species were also precipitated by FLAG resin during DNA replication (Figure 1D, lane 6). When DNA replication initiation was blocked with Geminin (Figure S1B) (Tada et al., 2001, Wohlschlegel et al., 2000), M.HpaII persisted in a largely unmodified form (Figure 1B, lanes 5–6), demonstrating that DPC ubiquitylation and degradation are dependent on DNA replication. In the absence of replication, a different set of modified M.HpaII species slowly appeared (Figure 1B, lane 6). These species were cleaved by the SUMO protease Ulp1 (data not shown), and their appearance was dependent on the activity of the SUMO ligase UBC9 (Figure S1C). In contrast, the replication-dependent species did not involve SUMOylation (Figure S1D). Therefore, DPCs undergo both replication-dependent ubiquitylation, which contributes to proteolysis (see below), and replication-independent SUMOylation, the function of which is still unknown.

Replication forks promote the destruction of DPCs encountered on the leading and lagging strand templates (Duxin et al., 2014). However, because the replicative Cdc45-Mcm-GINS (CMG) helicase translocates on the leading strand template (Fu et al., 2011), it is possible that DPCs encountered on the two parental strands undergo different processing. To address this, we replicated a plasmid containing a lac repressor array that is flanked on one side by a DPC on the top or bottom strand (Figure 1E). The rightward fork stalls at the array, whereas the leftward fork encounters the DPC on the leading or lagging strand templates, respectively (Dewar et al., 2015, Duxin et al., 2014). As shown in Figure 1E, DPCs encountered on either strand were ubiquitylated and degraded, suggesting that leading and lagging strand DPCs are recognized and processed similarly.

We next asked whether a specific ubiquitin linkage is formed on the DPC. The obutain1 and associated molecule with the SH3 domain of the signal transducing adaptor molecule (AMSH) deubiquitinases (DUBs), which are specific for Lys-48 and Lys-63 linkages, respectively, partially reduced the length of M.HpaII-linked ubiquitin chains (Figure S1E). YOD1, which hydrolyzes all of the other ubiquitin linkages, also partially cleaved DPC ubiquitin chains (Figure S1E). Polyubiquitylated M.HpaII persisted even after treatment with a DUB cocktail that targeted all linkages (Figure S1F), but treatment with ubiquitin carboxyl-terminal hydrolase 2 (USP2), which cleaves ubiquitin moieties, attached directly to target proteins (Hospenthal et al., 2015), collapsed M.HpaII into a single band (Figures S1E and S1F). These results suggest that ubiquitylated M.HpaII contains multiple ubiquitin chain types added by one or more ubiquitin ligases.

Previously, we demonstrated that DPC degradation is drastically inhibited by ubiquitin-vinyl-sulfone (UbVS), which inhibits DUBs and thereby depletes free ubiquitin in extracts (Dimova et al., 2012, Duxin et al., 2014). Consistent with this result, UbVS delayed the ubiquitylation of M.HpaII and strongly inhibited DPC proteolysis (Figure S1G, lanes 7–11), effects that were rescued with free ubiquitin (Figure S1G, lanes 12–16). We conclude that when a replication fork encounters a DPC, the DPC undergoes extensive polyubiquitylation before being degraded.

### SPRTN and the Proteasome Accumulate on Replicating DPC Plasmids

**Figure 2:**
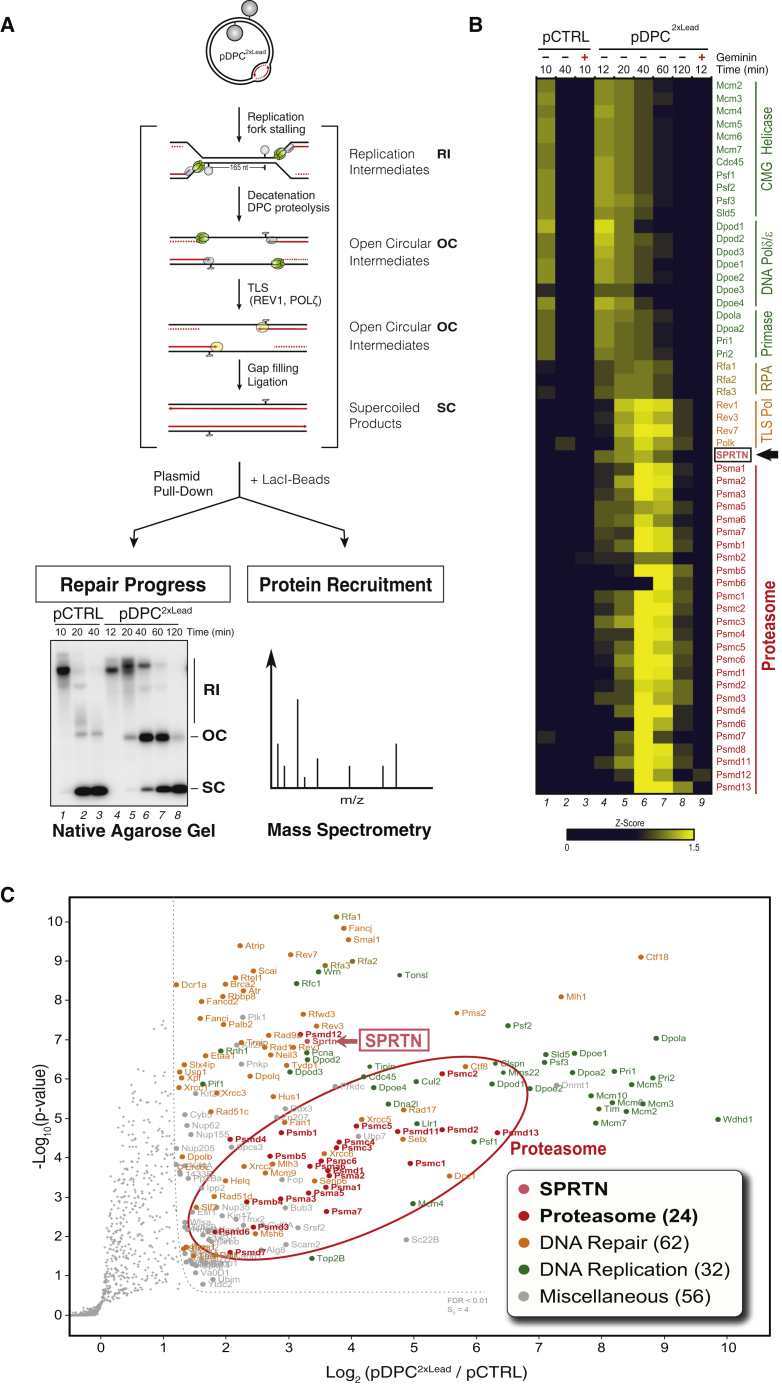
SPRTN and the Proteasome Are Recruited to a DPC Plasmid during Replication (A) Depiction of replication, recovery, and analysis of pDPC^2xLead^ by PP-MS. To monitor the progress of the repair reaction, pDPC^2xLead^ was replicated in the presence of [α-^32^P]dATP, and replication intermediates were analyzed by agarose gel electrophoresis (lower autoradiograph). In parallel, plasmids were isolated together with the bound proteins by a LacI pull-down (Budzowska et al., 2015) and analyzed by label-free MS. (B) Heatmap showing the mean of the *Z* scored log_2_ label-free quantitation LFQ intensity from four biochemical replicates of pCTRL and pDPC^2xLead^. Geminin was added to block replication where indicated. (C) Analysis of protein recruitment to pDPC^2xLead^ compared to pCTRL. Both plasmids were recovered at 40 min. The volcano plot shows the mean difference of the protein intensity plotted against the p value calculated by a modified, one-sided t test. Full results are reported in Tables S1 and S2.

To identify DPC protease(s), we combined plasmid pull-down with quantitative high-resolution mass spectrometry (PP-MS) (Figure 2A). In contrast to chromatin MS (CHROMASS), which detects proteins on randomly damaged sperm chromatin (Räschle et al., 2015), PP-MS identifies proteins associated with defined DNA lesions and discrete repair intermediates (Figure 2A). To validate PP-MS, we first replicated an undamaged plasmid (pCTRL) in egg extracts and isolated it during (10 min) or after (40 min) replication (Figure 2A, lanes 1–3). As expected, CMG, all three replicative DNA polymerases, and most of the replisome components, were significantly enriched when replication was ongoing (Figures 2B, columns 1–2, and S2A), whereas the addition of Geminin abolished their recruitment (Figures 2B, column 3, S2A, and S2B). Thus, PP-MS is a robust method to detect the proteins associated with plasmids in egg extracts.

We next applied PP-MS to DPC repair. To maximize the yield of DPC repair factors, we replicated pDPC^2xLead^, a plasmid containing two DPCs positioned 165 nt apart, such that both converging forks encountered a DPC on the leading strand template (Figure 2A). Following fork stalling at the DPC, the daughter molecules underwent decatenation, and the OC plasmids were repaired by TLS (Figures 2A, lanes 4–8, and S2C). Consistent with replication fork stalling at leading strand DPCs (Figure S2C) (Duxin et al., 2014, Fu et al., 2011), replisome components persisted for up to 40 min on pDPC^2xLead^ (Figures 2B, columns 4–8, and S2D, MCM6 panel). The TLS polymerases REV1, Polζ, and Polκ were recruited to pDPC^2xLead^ following replisome unloading (Figures 2B and S2D, REV1 panel), and their peak binding correlated with the transition from OC to SC plasmids (Figure 2A, lanes 6–7), which depends on the REV1-Polζ complex (Duxin et al., 2014). By 120 min, when all of the molecules had undergone replication-coupled repair (Figures 2A, lane 8, and 2C), repair factors were largely lost from DNA (Figure 2B, column 8).

Consistent with recent findings that SPRTN functions in S phase DPC repair, we observed a specific enrichment of SPRTN on replicating pDPC^2xLead^ (Figure 2B, columns 4–8). SPRTN recruitment occurred during the peak of proteolysis (20–60 min), depended on DNA replication, and was not observed on pCTRL (Figures 2B, 2C, S2D, and S2E). The 26S proteasome was also specifically recovered on DPC plasmids. A total of 26 proteasome subunits showed significant enrichment on pDPC^2xLead^ compared to pCTRL (Figure 2B), and their recruitment also peaked between 20 and 60 min and depended on DNA replication (Figures 2B, 2C, S2D, and S2E). Collectively, these experiments provide an unbiased resource of candidate DPC repair factors (Tables S1 ans S2) and single out SPRTN and the proteasome as two proteases that may mediate DPC destruction in egg extracts. They also illustrate the ability of PP-MS to identify proteins associated with different stages in the repair of a chemically defined DNA lesion.

### Both SPRTN and the Proteasome Degrade DPCs during Replication

**Figure 3:**
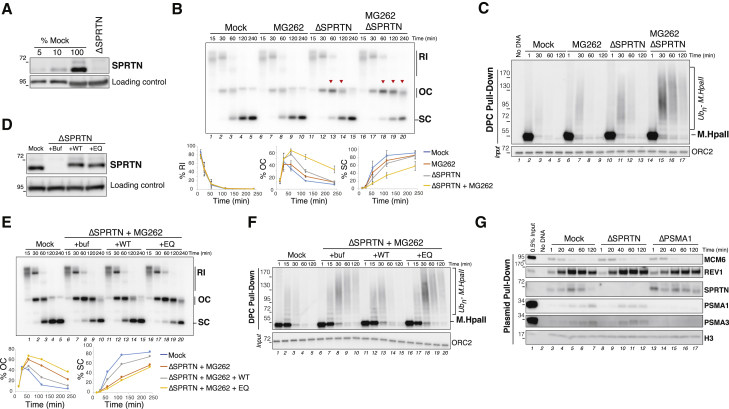
SPRTN and the Proteasome Degrade DPCs during Replication (A) Mock-depleted and SPRTN-depleted egg extracts were blotted with SPRTN and MCM6 (loading control) antibodies. (B) The extracts from (A) were used to replicate pDPC^2xLead^ in the presence of [α-^32^P]dATP. MG262 at 200 μM was added where indicated. Samples were analyzed by native agarose gel electrophoresis. Red arrowheads indicate the accumulation of OC repair intermediates. Note that OC molecules that accumulate are subjected to 5′ to 3′ end resection and smear-down on the gel (lanes 14, 19, and 20;Duxin et al., 2014). Replication intermediates (RI), open circular (OC), and supercoiled species (SC) were quantified as a percentage of total lane signal (lower graphs). The mean percentages across three independent experiments are plotted, with error bars representing the SD. (C) DPCs from (B) were recovered and monitored as in Figure 1B. (D) Mock-depleted and SPRTN-depleted egg extracts were blotted with SPRTN and MCM6 (loading control) antibodies. SPRTN-depleted extracts were supplemented with either buffer (+Buf), recombinant FLAG-SPRTN (+WT), or recombinant catalytically inactive FLAG-SPRTN E89Q (+EQ). (E) The extracts from (D) were used to replicate pDPC^2xLead^ in the presence of [α-^32^P]dATP. MG262 at 200 μM was added where indicated. Samples were analyzed and quantified as in (B). The quantification of a representative biological replicate is shown. (F) DPCs from (E) were monitored as in Figure 1B. (G) Mock-depleted, SPRTN-depleted, or proteasome subunit *α* type-1 (PSMA1)-depleted extracts were used to replicate pDPC^2xLead^. Plasmids were recovered, and protein-recruitment to the plasmid was monitored with the indicated antibodies (Budzowska et al., 2015).

To explore the roles of SPRTN and the proteasome in DPC proteolysis, we replicated pDPC^2xLead^ in the presence of the proteasome inhibitor MG262 or in extracts depleted of SPRTN (Figure 3A). Proteasome inhibition did not significantly inhibit DPC repair and only caused a minimal delay in the conversion of OC intermediates to SC repair products (Figure 3B, lanes 6–10) (Duxin et al., 2014). SPRTN-depleted extracts exhibited a more pronounced but still transient persistence of OC molecules compared to mock-depleted extracts (Figure 3B, lanes 11–15). In combination, however, SPRTN depletion and MG262 treatment strongly delayed the conversion of OC intermediates to SC products (Figures 3B, lanes 16–20) without affecting DNA replication kinetics (Figure S3A), indicating that in the absence of both proteases, DPC repair is specifically inhibited. Accordingly, whereas SPRTN depletion or MG262 treatment alone resulted in a modest delay in DPC degradation (Figures 3C, lanes 6–13, S3B, and S3C for independent experiments), in combination these treatments stabilized ubiquitylated M.HpaII species for up to 2 hr (Figures 3C, lanes 14–17, S3B, and S3C). DPC degradation and generation of SC repair products were largely restored by the addition of recombinant wild-type (WT) SPRTN but not catalytically inactive (EQ) SPRTN (Figures 3D–3F). We also confirmed the role of the proteasome on DPC degradation via immunodepletion, which closely resembled MG262 treatment (Figures S3D and S3E).

Given that depletion of SPRTN or inhibition of the proteasome did not prevent DPC degradation, we hypothesized that these two proteases act independently. To test this idea, we pulled down pDPC^2xLead^ from extracts depleted of either SPRTN or the proteasome. As shown in Figure 3G, neither SPRTN nor proteasome depletion impaired the recruitment of the other protease to chromatin. We conclude that during DNA replication, both SPRTN and the proteasome can degrade DPCs independently of each other.

### SPRTN Can Degrade Non-ubiquitylated DPCs

**Figure 4:**
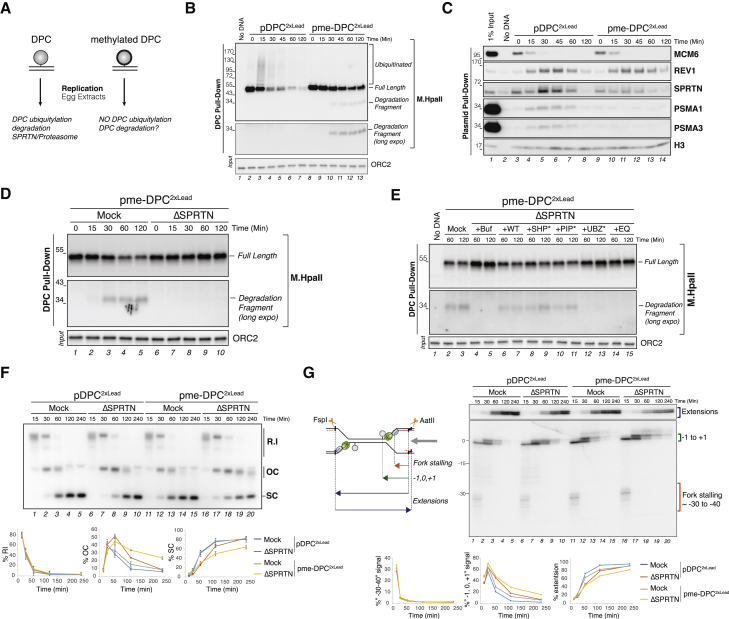
SPRTN, but Not the Proteasome, Can Degrade Non-ubiquitylated DPCs (A) Strategy to address the role of DPC ubiquitylation via reductive methylation of the DPC. (B) pDPC^2xLead^ and pme-DPC^2xLead^ were replicated in egg extracts, and DPCs were monitored as in Figure 1B. Note the concomitant disappearance of full-length M.HpaII and appearance of a degradation product during replication of pme-DPC^2xLead^. Both a long and a short exposure of the M.HpaII blot are shown. (C) pDPC^2xLead^ and pme-DPC^2xLead^ were replicated in egg extracts, and recruitment of the indicated proteins to the plasmid was monitored as in Figure 3G. (D) pme-DPC^2xLead^ was replicated in mock-depleted or SPRTN-depleted egg extracts. DPCs were monitored as in Figure 1B. (E) pme-DPC^2xLead^ was replicated in mock-depleted and SPRTN-depleted extracts. SPRTN-depleted extracts were supplemented with either buffer (+buf), or recombinant FLAG-SPRTN variants (see Figure S4D). DPCs were monitored as in Figure 1B. (F) pDPC^2xLead^ and pme-DPC^2xLead^ were replicated in mock-depleted or SPRTN-depleted extracts. Samples were analyzed as in Figure 3B. The mean of three independent experiments is quantified. Error bars represent the SD. (G) Samples from (F) were digested with FspI and AatII and separated on a denaturing polyacrylamide gel. The schematic depicts the nascent leading strands and extension products liberated by FspI and AatII digestion (green hexamer, CMG helicase; red lines, nascent DNA). The locations of the corresponding bands on the gel are indicated by brackets. The −30 to −40 species, the −1,0,1 species, and extension products were quantified and plotted below. Quantification of each species is plotted as a percentage of the entire signal of the lane. The quantification of a representative biological replicate is shown.

We next investigated the role of DPC ubiquitylation. To this end, we chemically methylated the lysines of M.HpaII (Walter et al., 2006), thereby generating a DPC that cannot be ubiquitylated (me-DPC) (Figure 4A). As shown in Figure 4B, methylated M.HpaII recovered from replication reactions migrated as a single, unmodified band, reflecting a block of DPC ubiquitylation (lanes 8–13). Notably, even in the absence of ubiquitylation, plasmid-associated M.HpaII slowly decreased, and an M.HpaII degradation product of ∼34 kDa accumulated (Figure 4B, lanes 10–13; note that the M.HpaII antibody is detecting one of possibly multiple degradation products). In plasmid pull-downs, M.HpaII methylation abolished proteasome recruitment, while SPRTN recruitment was reduced but still detectable (Figure 4C, lanes 9–14), suggesting that SPRTN is the sole protease acting on the methylated DPC. Consistent with this idea, SPRTN depletion stabilized methylated M.HpaII and abolished the formation of the degradation fragment (Figure 4D, lanes 6–10). This defect was reversed by SPRTN-WT but not SPRTN-EQ (Figure 4E, lanes 6–7 and 14–15). Conversely, MG262 did not prevent me-DPC proteolysis (Figures S4A and S4B). Consistent with the absence of DPC proteolysis, SPRTN depletion also caused a marked stabilization of OC intermediates during the replication of pme-DPC^2xLead^ and a corresponding delay in the generation of replicated SC molecules (Figure 4F, lanes 16–20). We conclude that SPRTN has a unique ability to degrade non-ubiquitylated DPCs. In contrast, DPC ubiquitylation is essential to target the proteasome.

### SPRTN Requires Its Ubiquitin Binding Motifs to Degrade DPCs

We next explored the importance of the protein-interacting regions of SPRTN. In addition to its SprT metalloprotease domain, SPRTN contains C-terminal p97 (SHP), proliferating cell nuclear antigen (PCNA) (PIP), and ubiquitin (UBZ)-interacting regions (Figure S4C). We generated recombinant SPRTN with mutated SHP, PIP, or UBZ domains (Figures S4D and S4E; Davis et al., 2012, Mosbech et al., 2012) and tested their activity in pme-DPC^2xLead^ replication. Whereas SPRTN depletion blocked degradation of me-DPCs, re-addition of SPRTN-SHP^∗^ or SPRTN-PIP^∗^ reverted this effect (Figure 4E, lanes 8–9 and 10–11), suggesting that interactions with p97 and PCNA are not essential for the function of SPRTN as a DPC protease. In contrast, SPRTN-UBZ^∗^ failed to restore DPC proteolysis after SPRTN depletion (Figure 4E, lanes 12–13). Efficient generation of SC repair products was likewise supported by SPRTN-SHP^∗^ and SPRTN-PIP^∗^, but not by SPRTN-UBZ^∗^ (Figure S4F). The role of SPRTN ubiquitin binding domains was confirmed by deleting both C-terminal UBZ domains (SPRTN 1–435), which also resulted in defective DPC degradation and replication (Figures S4G and S4H). In summary, our data demonstrate that SPRTN can act on DPCs in the absence of DPC ubiquitylation. However, SPRTN activity is still dependent on its ubiquitin-interacting motifs, suggesting that SPRTN is recruited by a ubiquitylated protein other than the DPC itself.

### SPRTN-Mediated DPC Proteolysis Ensures Efficient TLS

As shown above, SPRTN depletion leads to a transient accumulation of OC intermediates during the replication of a DPC plasmid. To determine how SPRTN depletion affects replication across a DPC, we first replicated pDPC^2xLead^ and analyzed nascent leading strands on a denaturing polyacrylamide gel (Figure 4G) (Duxin et al., 2014). In mock-depleted extracts, the nascent leading strand first paused 30 to 40 nt upstream of the DPC due to the footprint of CMG, which stalled at the DPC (fork stalling) (Figure 4G, lanes 1–5). Subsequently, the nascent leading strand advanced and stalled again at the lesion site (−1, 0, +1) before being extended past the lesion via TLS polymerases REV1-Polζ (extension) (Figures 4G and S4I for the annotation of −1, 0, and +1 products). SPRTN depletion did not inhibit leading strands from reaching the lesion, but it did prolong the stalling observed at −1, 0, and +1 positions by ∼30 min, which correlated with a delay in the appearance of the extension products (Figures 4G, lanes 6–10, S4K, and S4M). This TLS defect was rescued by SPRTN-WT but not SPRTN-EQ, which further inhibited synthesis across the lesion (Figures S4J and S4K). The inhibitory effect of SPRTN depletion was neutralized by pre-treatment of the DPC with proteinase K (Figures S4L–S4N). We conclude that SPRTN-mediated DPC proteolysis facilitates TLS past the lesion. In the absence of SPRTN, the DPC is still degraded by the proteasome, but TLS across the resulting peptide adduct generated by the proteasome is likely not as efficient.

We next investigated the role of SPRTN in facilitating replication across the methylated DPC, which cannot be acted on by the proteasome. In this context, SPRTN depletion also induced a marked TLS defect, as seen by the prolonged stalling of leading strands at the DPC (Figure 4G, lanes 16–20). Despite the absence of DPC proteolysis, the approach of leading strands to the lesion was unaffected (Figure 4G, see disappearance of −30 to −40 products), demonstrating that CMG disappears from the DPC on schedule. This loss of the CMG footprint is due to CMG bypass of the intact DPC (Sparks et al., 2018). Thus, in the presence and absence of the proteasome pathway, SPRTN is required to facilitate TLS across the lesion.

### SPRTN and the Proteasome Can Degrade DPCs in the Absence of the Replisome

**Figure 5:**
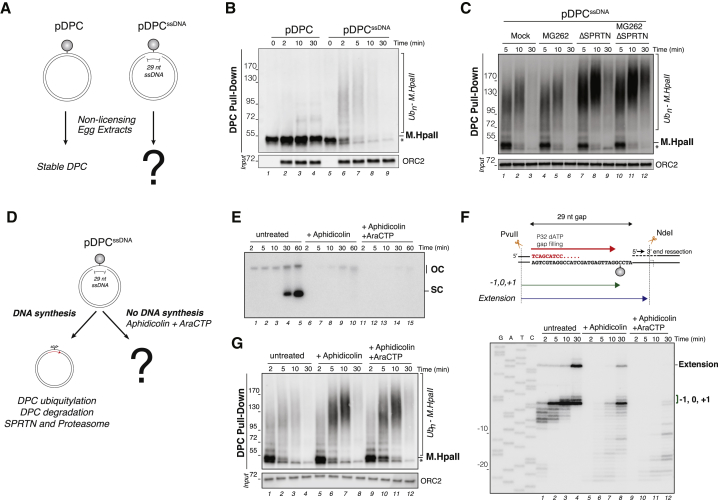
DPC Ubiquitylation and Degradation Can Occur in the Absence of the Replisome (A) Schematic comparing pDPC and pDPC^ssDNA^ in non-licensing egg extracts. (B) pDPC and pDPC^ssDNA^ were incubated in non-licensing egg extracts. DPCs were recovered and monitored as in Figure 1B. Note that time 0 was withdrawn before incubating plasmids in egg extracts, which explains the absence of ORC2 input in lanes 1 and 5. (C) pDPC^ssDNA^ was incubated in mock-depleted and SPRTN-depleted non-licensing extracts in the presence of 200 μM MG262 where indicated. DPCs were monitored as in Figure 1B. (D) Schematic comparing the fate of pDPC^ssDNA^ in the presence or absence of gap-filling synthesis. (E) pDPC^ssDNA^ was incubated in non-licensing egg extracts in the presence of [α-^32^P]dATP. Extracts were supplemented with 700 μM aphidicolin and 1 mM araCTP where indicated. Samples were analyzed as in Figure 3B. (F) Samples from (E) were digested with PvuII and NdeI and separated on a denaturing polyacrylamide gel. The different extension products are depicted in the upper scheme. (G) Samples from (E) were used to monitor DPC ubiquitylation and degradation as in Figure 1B.

We next addressed how SPRTN and proteasome activities are coupled to DNA replication. In one scenario, the replisome recruits or activates these proteases. Alternatively, DNA replication generates a structure that targets the proteases to DPCs. To distinguish between the two models, we tested whether ssDNA could trigger DPC degradation in the absence of the replisome. To this end, we generated a plasmid in which M.HpaII is linked to one strand across from a 29-nt gap (pDPC^ssDNA^; Figure 5A). We then monitored M.HpaII degradation on pDPC^ssDNA^ in extracts that do not support MCM2–7 loading or replication initiation (non-licensing extracts). In this setting, pDPC^ssDNA^ triggered rapid polyubiquitylation and degradation of M.HpaII, whereas pDPC did not (Figure 5B, lanes 1–4 and 6–9). In addition, on pDPC^ssDNA^, ubiquitylated M.HpaII species were stabilized most by the combined inhibition of the proteasome and depletion of SPRTN (Figures 5C, lanes 10–12, and S5A–S5C). Therefore, both SPRTN and the proteasome can degrade DPCs in the absence of a full replisome when the lesion resides on ssDNA. Consistent with this conclusion, purified Wss1 and SPRTN are activated by ssDNA (Balakirev et al., 2015, Stingele et al., 2016).

### Polymerase Extension Controls SPRTN-Mediated DPC Degradation

When pDPC^ssDNA^ was incubated in non-licensing extracts, we detected a small amount of DNA synthesis that was absent on pDPC (Figures S5D and S5E). This synthesis reflected extension of the free 3′ end to the DPC, followed by TLS past the lesion (Figure S5F). We asked whether this gap-filling synthesis is required to trigger DPC ubiquitylation and degradation (Figure 5D). To this end, we treated egg extracts with aphidicolin, which greatly diminished gap filling, or with a combination of aphidicolin and the chain terminator *ara*-cytidine-5′-triphosphate (araCTP), which inhibited gap filling almost completely (Figures 5E and 5F). As shown in Figure 5G, aphidicolin or aphidicolin and araCTP impaired DPC proteolysis, although M.HpaII ubiquitylation occurred normally. Thus, efficient DPC proteolysis but not DPC ubiquitylation requires gap-filling synthesis.

**Figure 6:**
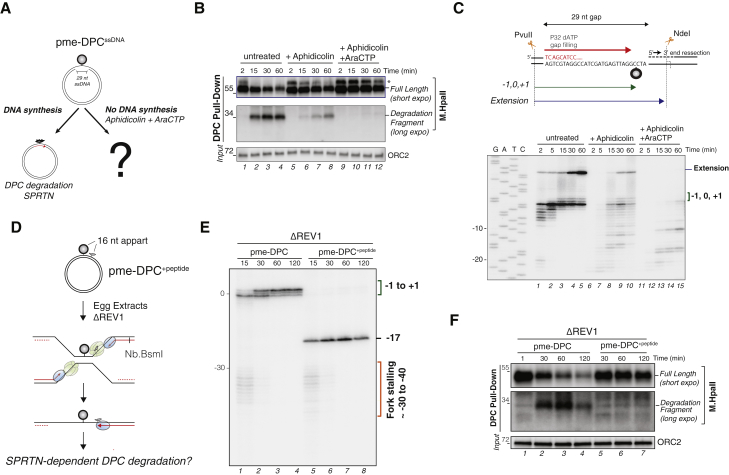
SPRTN-Dependent DPC Degradation Requires Nascent Strand Extension to the Lesion (A) Schematic comparing the fate of pme-DPC^ssDNA^ in the presence and absence of gap-filling synthesis. (B) pme-DPC^ssDNA^ was incubated in non-licensing egg extracts supplemented with 700 μM aphidicolin and 1 mM araCTP where indicated. DPC degradation was monitored as in Figure 1B. The asterisk denotes a crosslinked methyl-M.HpaII species generated on the GAP substrate, likely caused by the incomplete degradation of ssDNA by benzonase. (C) Samples from (B) were analyzed as in Figure 5F. (D) Depiction of pme-DPC^+peptide^ replication. (E) pme-DPC and pme-DPC^+peptide^ were replicated in REV1-depleted extracts in the presence of [α-^32^P]dATP. Samples were digested with Nb.BsmI, which cuts the leftward leading strand, as depicted in (D). Nascent leading strands were then separated on a polyacrylamide denaturing gel. (F) Samples from (E) were used to monitor DPC degradation as in Figure 1B.

Given that DPC degradation but not ubiquitylation was delayed, we reasoned that DPC proteolysis by SPRTN but not the proteasome requires gap-filling synthesis. To monitor SPRTN activity, we examined a gapped substrate containing methylated M.HpaII (Figure 6A). As seen during DNA replication, methylated M.HpaII underwent ubiquitylation-independent degradation, giving rise to the SPRTN-dependent proteolytic fragment (Figure S5G). The efficiency of proteolysis and appearance of the DPC fragment correlated with the amount of gap-filling synthesis, as these were partially inhibited by aphidicolin (Figures 6B, lanes 5–8, and 6C, lanes 6–10) and completely blocked by the combination of aphidicolin and araCTP (Figures 6B, lanes 9–12, and 6C, lanes 11–15). In contrast, if aphidicolin and araCTP were added at 3 min, when the majority of 3′ ends had reached the crosslink (Figures S6A–S6C) but before the marked accumulation of the DPC fragment, M.HpaII degradation occurred normally (Figure S6D, lanes 5–8). We conclude that nascent strand extension to the immediate vicinity of the DPC is a prerequisite to trigger SPRTN-mediated DPC degradation in the context of pDPC^ssDNA^.

To test whether strand extension triggers SPRTN activity at a replication fork, a short peptide adduct was placed 16 nt upstream of the methylated DPC, yielding pme-DPC^+peptide^ (Figure 6D). The peptide should inhibit polymerase extension while having no impact on CMG progression. To ensure that no leading strands reached the DPC, pme-DPC^+peptide^ was replicated in REV1-depleted extracts. A matched pme-DPC substrate lacking the peptide served as a control. As seen in Figure 6E, after first pausing at the −30 to −40 positions due to CMG collision with the DPC, leading strands on pme-DPC^+peptide^ were extended but then permanently stalled at the upstream peptide adduct (lanes 5–8, −17 position). Under these conditions, methylated M.HpaII persisted, and the SPRTN-mediated proteolytic fragment never appeared (Figure 6F, lanes 5–7). In contrast, M.HpaII proteolysis proceeded normally on pme-DPC (Figure 6F, lanes 1–4), where leading strands were allowed to reach the DPC (Figure 6E, lanes 1–4). These results demonstrate that in the context of replication, CMG-DPC collision is insufficient to activate SPRTN. Instead, SPRTN activity is strictly dependent on the subsequent extension of a nascent strand to the lesion, which can only occur once the CMG helicase has dissociated or moved past the protein adduct.

Having defined the requirements for SPRTN proteolysis, we repeated the experiment with unmethylated M.HpaII, which should be ubiquitylated and therefore degraded by the proteasome in the absence of polymerase extension. To this end, we replicated pDPC^+peptide^ in REV1-depleted extracts in the presence or absence of MG262 (Figure S6E). Unmethylated M.HpaII underwent rapid polyubiquitylation and degradation, although leading strands never reached the lesion (Figures S6F and S6G). In this context, MG262 stabilized polyubiquitylated M.HpaII (Figure S6G). Thus, DPC ubiquitylation and degradation by the proteasome do not require polymerase advancement to the lesion site.

### TRAIP Stimulates DPC Ubiquitylation and Proteasome Targeting

**Figure 7:**
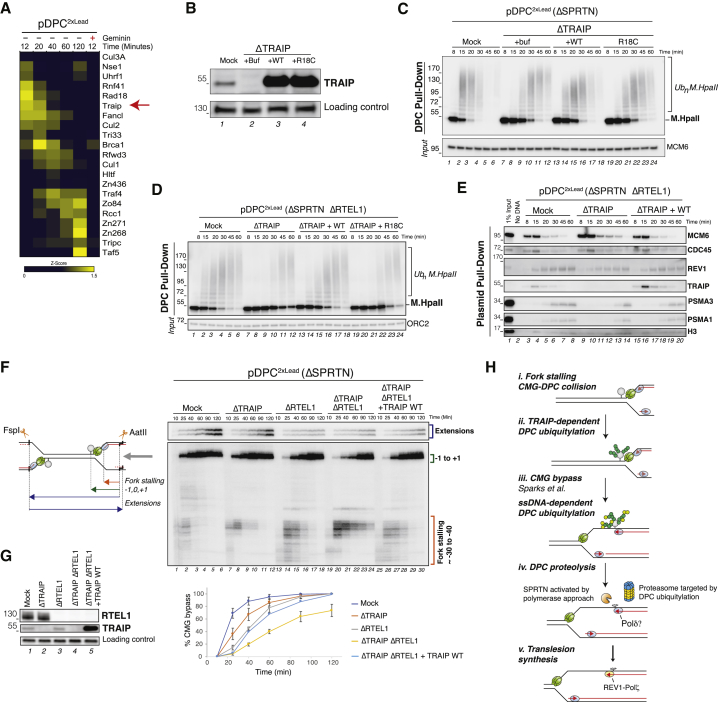
TRAIP Ubiquitin Ligase Stimulates DPC Ubiquitylation and Proteasome Targeting (A) Heatmap showing the mean of the *Z* scored log2 LFQ intensity of potential E3 ubiquitin ligases. Proteins with similar intensities in the geminin or mock control lacking the DNA substrate were excluded. (B) Extracts were depleted with SPRTN and either control immunoglobulin G (IgG) or TRAIP antibodies and blotted for TRAIP and RTEL1 (loading control). TRAIP-depleted extracts were supplemented with buffer (+Buf), recombinant TRAIP(WT), or TRAIP(R18C). (C) Extracts from (B) were used to replicate pDPC^2xLead^. DPCs were monitored as in Figure 1B. (D) SPRTN- and RTEL1-depleted extracts were either mock depleted or TRAIP depleted. TRAIP-depleted extracts were supplemented with buffer (+Buf), recombinant TRAIP(WT), or TRAIP(R18C). These extracts were used to replicate pDPC^2xLead^, and DPCs were monitored as in Figure 1B. (E) The indicated extracts were used to replicate pDPC^2xLead^. Recruitment of the indicated proteins to the plasmid was monitored as in Figure 3G. (F) Extracts described in (D) were used to replicate pDPC^2xLead^ in the presence of [α-^32^P]dATP, and nascent strand intermediates were analyzed as in Figure 4G. CMG bypass was measured based on the disappearance of the −30 to −40 CMG footprint (Sparks et al., 2018). The mean of three independent experiments is graphed for mock-, TRAIP-, and RTEL1-TRAIP-depleted samples. Error bars represent the SD. The TRAIP-RTEL1-depleted samples supplemented with TRAIP(WT) represents the mean of two experiments and plotted without error bars. (G) Samples from (F) were blotted with TRAIP, RTEL1, or SLD5 (loading control) antibodies. (H) Model for replication-coupled DPC proteolysis in *Xenopus* egg extracts. Black lines, parental DNA; red lines, nascent DNA; green hexamers, CMG helicase; blue spheres, replicative polymerases; yellow spheres, TLS polymerase; gray sphere, DPC; orange, SPRTN; yellow and blue, the proteasome; dark green, TRAIP-dependent ubiquitin chains; light green, ubiquitin chains deposited by a second E3 ligase activated by ssDNA.

Finally, we addressed which E3 ligase ubiquitylates the DPC. Our PP-MS analysis identified several ubiquitin ligases that were enriched on replicating pDPC^2xLead^ (Figure 7A). Among these, TRAIP was a good candidate because it was strongly enriched early in the reaction at the onset of DPC ubiquitylation (Figure 7A, 12 and 20 min), and its recruitment to chromatin depended on replication (Figure 7A, ±Geminin). Moreover, TRAIP-deficient cells exhibit impaired replication fork progression upon stress and sensitivity to crosslinking agents (Feng et al., 2016, Harley et al., 2016, Hoffmann et al., 2016). To clearly monitor DPC ubiquitylation and proteasome-mediated DPC degradation, we performed experiments in the absence of SPRTN. In this setting, TRAIP depletion delayed DPC ubiquitylation and degradation by 10–15 min compared to the mock reaction (Figures 7B, 7C, lanes 1–12, S7A, and S7B) without affecting DNA replication kinetics (Figure S7C). Both DPC ubiquitylation and proteolysis were largely restored by recombinant wild-type (WT) TRAIP (Figures 7B and 7C, lanes 13–18), but not a TRAIP mutant harboring an amino acid substitution in the RING domain (R18C) (Figures 7B and 7C, lanes 19–24) that causes primordial dwarfism (Harley et al., 2016). In contrast, when pDPC^ssDNA^ was incubated in non-licensing extracts depleted of TRAIP, M.HpaII ubiquitylation was unaffected (Figure S7D), suggesting the existence of a second E3 ligase that operates on the DPC in the context of ssDNA. These results indicate that TRAIP promotes DPC ubiquitylation and proteolysis by the proteasome during replication.

We recently demonstrated that in the absence of DPC proteolysis, CMG bypasses an intact DPC, and this bypass is required for efficient DPC proteolysis (J.L.S., unpublished data). Moreover, we showed that bypass requires the DNA helicase regulator of telomere elongation helicase 1 (RTEL1). In the absence of RTEL1, ubiquitin chains form on the DPC with normal kinetics, but they are shorter, indicating that the DPC can be ubiquitylated even before CMG has bypassed the lesion (Figure S7E, compare lanes 2–3 and 8–9 and J.L.S., unpublished data). Based on these findings, we postulated that TRAIP promotes DPC ubiquitylation upon replisome collision with the DPC and that subsequent CMG bypass generates ssDNA surrounding the DPC, stimulating DPC ubiquitylation by the second, ssDNA-activated E3 ligase. If this is the case, then the dependence of DPC ubiquitylation on TRAIP should be more pronounced in the absence of RTEL1. In RTEL1- and SPRTN-depleted extracts, TRAIP depletion delayed M.HpaII ubiquitylation by up to 30 min (Figures 7D, lanes 7–12, and S7E), and this effect was rescued by TRAIP WT but not TRAIP R18C (Figure 7D, lanes 13–24). Consistent with the inhibition in DPC ubiquitylation, TRAIP depletion in the absence of RTEL1 delayed proteasome recruitment to chromatin (Figure 7E, compare lanes 3–8 and lanes 9–14), which was partially rescued by TRAIP WT (Figure 7E, lanes 15–20). While DNA replication kinetics were unaffected by TRAIP depletion (Figures S7C and S7F), both in the presence and absence of RTEL1, TRAIP depletion delayed the disappearance of the CMG footprint at the DPC, which is consistent with a defect in CMG bypass (Figures 7F, lanes 1–12 and 13–24, and 7G). This effect on CMG bypass was not observed in methylated DPC (Figure S7G), strongly suggesting that the relevant target of TRAIP ubiquitylation is the DPC. We conclude that DPC ubiquitylation and proteasome targeting occurs in two stages: first, when forks collide with a DPC, TRAIP-dependent DPC ubiquitylation promotes CMG bypass, and second, CMG bypass creates ssDNA surrounding the DPC that enables further DPC ubiquitylation by a second, unknown E3 ubiquitin ligase.

## Discussion

We previously demonstrated that DPCs are degraded in a replication-dependent process, but how this occurs was unclear (Duxin et al., 2014). Using a newly developed PP-MS proteomic workflow, we identified SPRTN and the proteasome as two proteases that are recruited to a DPC lesion during DNA replication. We further demonstrate that SPRTN and the proteasome operate in different pathways that are differentially activated by DNA replication (Figure 7H). The implications of these findings are discussed below.

### Polymerase Approach Targets SPRTN

Our results raise the possibility that the stalling of a DNA polymerase at a DPC targets and activates SPRTN. Previous studies reported direct interactions between SPRTN and DNA polymerase delta subunit 3 (POLD3), one of the accessory subunits of DNA Polδ (Ghosal et al., 2012, Kim et al., 2013), suggesting that Polδ may direct SPRTN to the DPC. To date, we have not been able to achieve sufficient depletion of Polδ from egg extracts to prevent gap-filling synthesis (data not shown), precluding a direct test of this model. Purified SPRTN is activated by DNA *in vitro* (Lopez-Mosqueda et al., 2016, Mórocz et al., 2017, Stingele et al., 2016, Vaz et al., 2016), with ssDNA being particularly potent (Stingele et al., 2016). We therefore speculate that SPRTN activation requires the presence of a DNA polymerase on one side of a DPC and a short tract of ssDNA on the other side. This dual requirement would specifically target SPRTN to DPCs during replication and avoid indiscriminate destruction of replisome components or other chromatin proteins.

A model of SPRTN activation by polymerase-DPC collision has numerous implications. First, because CMG blocks the ability of leading strands to reach a DPC on the leading strand template, our data imply that proteolysis by SPRTN can only occur if CMG is no longer present in front of the DPC (Figure 7F). Accordingly, we show that the CMG helicase readily bypasses leading strand DPCs and that this process requires the helicase activity of RTEL1 (Sparks et al., 2018). Consistent with a requirement for leading strand extension in SPRTN activity, in the absence of RTEL1-mediated DPC bypass, DPC proteolysis by SPRTN is impaired. Second, SPRTN-dependent DPC proteolysis can likely be uncoupled from the replication fork. Supporting this idea, we show that SPRTN efficiently degrades a DPC linked to ssDNA in the absence of the replisome via a process that mimics post-replicative repair (Figures 6A–6C). By restricting SPRTN to act behind the replication fork, cells ensure that irreplaceable replisome factors such as CMG are not accidentally degraded during replication. Finally, SPRTN activation by polymerase-DPC collision suggests a common mechanism of DPC degradation on the leading and lagging strands; if Polε remains associated with CMG during DPC bypass, this would liberate the leading strand for Polδ recruitment. In this way, both leading- and lagging-strand DPC proteolysis would be triggered by the collision of Polδ with the adduct.

SPRTN contains C-terminal domains that interact with p97, PCNA, and ubiquitin, but their importance in SPRTN activity is unclear. While some reports suggested that these domains are not essential (Maskey et al., 2014, Stingele et al., 2016), more recent evidence indicates that both the ubiquitin and PCNA binding interactions of SPRTN are important for its role as a DPC protease in human cells (Mórocz et al., 2017). In *Xenopus* egg extracts, the ubiquitin binding domain of SPRTN is important for efficient DPC proteolysis, even in the absence of DPC ubiquitylation, suggesting that SPRTN interacts with another ubiquitylated protein near the lesion. Given our model that polymerase-DPC collision triggers SPRTN activity, a possible candidate is PCNA, which is ubiquitylated during post-replicative repair (Hoege et al., 2002). Consistent with this idea, RAD18 is epistatic to SPRTN for DPC repair (Mórocz et al., 2017). Although the PIP motif was not required for SPRTN activity in egg extracts, this may reflect the presence of tandem UBZ domains in *Xenopus* SPRTN that could compensate for reduced PCNA binding.

### DPC Ubiquitylation Promotes Proteasome-Mediated DPC Degradation

It was previously proposed that the proteasome can degrade DPCs, but evidence that this process occurs during DNA replication was lacking. Our work in *Xenopus* egg extracts shows that replication triggers rapid polyubiquitylation of a type I DPC. When DPC ubiquitylation is prevented via lysine methylation of the DPC, proteasome recruitment is abolished, strongly supporting a role for DPC ubiquitylation in proteasome targeting. Although the interplay between TRAIP and the second E3 ubiquitin ligase and their specific functions in proteasome targeting are unclear at present, a comprehensive model for replication-coupled DPC ubiquitylation is starting to emerge (Figure 7H). Our data suggest that one of the earliest events following the replisome-DPC encounter is TRAIP-mediated ubiquitylation of the DPC (Figure 7H, i–ii). Three observations support this notion. First, TRAIP is enriched on the DPC plasmid early in the reaction before CMG bypass (already present at 12 min, before CMG bypass is observed [Figure S2C]). Second, TRAIP-dependent DPC ubiquitylation is independent of RTEL1 and therefore does not require CMG bypass (Figure 7D). Third, TRAIP depletion delayed CMG bypass, and this effect was abrogated by the methylation of the DPC (Figures 7F and S7G). Following CMG bypass, ssDNA surrounding the DPC likely stimulates the activity of the second E3 (Figure 7H, iii). Supporting this idea, we show here that when the DPC is linked to ssDNA, DPC ubiquitylation and proteasome-dependent DPC degradation occurs independently of the replisome or TRAIP (Figures 5 and S7D). Whether TRAIP-dependent DPC ubiquitylation promotes proteasome targeting independently of CMG bypass is an interesting question. For example, TRAIP ubiquitylation of impassable DPCs may gradually stimulate their processing by the proteasome to facilitate CMG bypass. The role of TRAIP in promoting efficient CMG bypass predicts that TRAIP should also be required for optimal SPRTN activity. TRAIP mutations in humans induce microcephalic primordial dwarfism (Harley et al., 2016). Future work will be required to address whether this phenotype is attributable to defective replication bypass and degradation of DPCs or other functions of TRAIP.

### SPRTN and the Proteasome Are Not Redundant DPC Proteases

How do our findings relate to the defective replication fork progression and formaldehyde sensitivity observed in SPRTN-deficient cells (Lessel et al., 2014, Vaz et al., 2016)? We showed that SPRTN depletion delays TLS, indicating that SPRTN and the proteasome are not redundant. We speculate that SPRTN is able to degrade DPCs to peptide adducts that are sufficiently small for efficient TLS. The protease active site of Wss1 is highly solvent exposed, suggesting that it should be able to cleave DPCs close to the DNA attachment site (Stingele et al., 2016). In contrast, the active sites of the proteasome are buried inside the 20S core particle. Threading of the unfolded DPC through the cylindrical 20S particle would likely be interrupted upon its encounter with the attached DNA, resulting in a larger peptide adduct. Thus, when DPCs are channeled into the proteasomal pathway, SPRTN may still be required in a second proteolytic step to reduce the peptide adduct to a few amino acids. Our findings predict that in SPRTN-deficient cells, CMG becomes uncoupled from the leading strand due to defective TLS. In bacteria, helicase uncoupling greatly slows the rate of DNA unwinding (Kim et al., 1996), and this is also true in vertebrates (J.L.S., unpublished data). Therefore, we speculate that defective fork progression in SPRTN-deficient cells reflects slow unwinding by uncoupled CMG. Defective TLS may also contribute to the formaldehyde sensitivity of SPRTN-deficient cells, as REV1- and REV3-deficient cells are also sensitive to formaldehyde treatment (Ridpath et al., 2007).

DPCs are expected to exhibit great variability in size, structure, and attachment chemistry. While agents such as formaldehyde crosslink proteins to duplex DNA, abortive reactions by topoisomerase form DPCs that are flanked by a DNA break. Hence, while both SPRTN and the proteasome readily degrade M.HpaII, it is conceivable that other crosslinked proteins are preferentially processed by one or the other protease. For example, SPRTN is expected to be particularly critical for DPCs that lack available lysines and therefore cannot be ubiquitylated, which may account for the DPCs that accumulate in SPRTN-deficient cells (Stingele et al., 2016, Vaz et al., 2016). In contrast, the proteasome may be essential for very large DPCs that cannot be bypassed by the replication fork and require “pre-trimming” by the proteasome. Alternatively, the proteasome may be critical in removing DPCs flanked by DNA breaks outside of replication or when DPCs are encountered by the transcription machinery. The use of at least two DPC proteases with orthogonal mechanisms and triggers represents a versatile system to degrade a wide variety of DPCs.

## STAR★Methods

### Key Resources Table

**Table:** 

REAGENT or RESOURCE	SOURCE	IDENTIFIER
**Antibodies**		

Rabbit polyclonal anti-Rev1-N	Budzowska et al., 2015	N/A
Rabbit polyclonal anti-Rev1-C	Budzowska et al., 2015	N/A
Rabbit polyclonal anti-Orc2	Fang and Newport, 1993	N/A
Rabbit polyclonal anti-Cdc45	Mimura and Takisawa, 1998	N/A
Rtel1	Sparks et al., 2018	N/A
Sld5	Dewar et al., 2017	N/A
Rabbit polyclonal anti-Psma1	New England Peptides	3514
Rabbit polyclonal anti-Psma3	New England Peptides	3516
Rabbit polyclonal anti-Sprtn	New England Peptides	3703
Rabbit polyclonal anti-Traip	New England Peptides	3472
Rabbit polyclonal anti-MCM6	New England Peptides	2926
Rabbit polyclonal anti-Sprtn-N	Pocono Rabbit Farm and Laboratory	Rabbit # 31053
Rabbit polyclonal anti-M.HpaII	Pocono Rabbit Farm and Laboratory	Rabbit # 31495 and 31496
Rabbit polyclonal anti-H3	Cell Signaling	Cat# 9715S; RRID:AB_331563

**Bacterial and Virus Strains**		

*E. coli* T7 express	New England Biolabs	C2566H
*E. coli* Rosetta 2 (DE3) pLysS	Novagen	71-401-3

**Chemicals, Peptides, and Recombinant Proteins**		

Geminin	McGarry and Kirschner, 1998	N/A
LacI-biotin	Duxin et al., 2014	N/A
M.HpaII	Duxin et al., 2014	N/A
*xl*SPRTN-WT	This study	N/A
*xl*SPRTN-EQ	This study	N/A
*xl*SPRTN-SHP^x^	This study	N/A
*xl*SPRTN-PIP^x^	This study	N/A
*xl*SPRTN-UBZ^x^	This study	N/A
*xl*SPRTN-1-435	This study	N/A
*xl*TRAIP-WT	This study	N/A
*xl*TRAIP-R18C	This study	N/A
UbVS	Boston Biochem	U-202
Human Recombinant Ubiquitin	Boston Biochem	U-100H
Human Recombinant FLAG-Ubiquitin	Boston Biochem	U-120
dnUBC9	Azuma et al., 2003	N/A
Human chorionic ganotropin	Sigma	CG10-10VL
MG262	Boston Biochem	I-120
Alpha-32P-deoxyadenosinetriphosphate	Perkin Elmer	BLU512H250UC
Gamma-32P-adenosinetriphosphate	Perkin Elmer	BLU502A100UC
Proteinase K, recombinant	Roche	3115879001
Aphidicolin	Sigma	A0781-1MG
Ara-cytidine-5′triphosphate	Jena Bioscience	NU-1170S
Exonuclease I	New England Bioscience	M0293S
Protein A Sepharose Fast Flow	GE Health Care	17-1279-01
RNase A	Thermo Fisher	EN0531
Gel Loading Dye II	Invitrogen	AM8547
EDTA-free Complete protease inhibitor cocktail	Roche	11873580001
Anti-FLAG M2 affinity resin	Sigma	A2220-5ML
3xFLAG peptide	Sigma	F4766-4MG
Streaptavidin-coupled magnetic beads M-280	Invitrogen	11205D
Benzonase	Novagen	70746-3
FLAG M2 magnetic beads	Sigma	M8823-1ML
His-tag Dynabeads	Thermo Fisher Scientific	10103D
LysC	Life Technologies	90051
Trypsin	Life Technologies	90305
AluI Methyltransferase	New England BioLabs	M0220S

**Critical Commercial Assays**		

UbiCREST	Boston Biochem	K-400
Reductive Alkylation Kit	Hampton Research	HR2-434
Quickchange II mutagenesis kit	Aglient	200523
Thermo Sequenase Cycle Sequencing kit	USB	785001KT
Bac to Bac Expression System	Thermo Fisher Scientific	10359016

**Deposited Data**		

ProteomeXchange	This study	PXD008831

**Experimental Models: Cell Lines**		

Sf9 Insect cells	Thermo Fosher Scientific	B82501

**Experimental Models: Organisms/Strains**		

*Xenopus laevis* (females)	Nasco	LM0053MX
*Xenopus laevis* (males)	Nasco	LM00715MX

**Oligonucleotides**		

JLS2: GGGAGCTGAATGCCGCGCGAATAATGGTTTCTTAGACGT	This study	N/A
JLS3: CATCCACTAGCCAATTTATGCTGAGGTACCGGATTGAGTA GCTACCGGATGCTGAGGGGAT CCACTAGCCAATTTATCATGG	This study	N/A
NBL104: AATTCCTCAGCATCCGGTTCGAACTCAATAGCTTACCT CAGCCA	This study	N/A
Gap_capture: GGTACCGGATTGAGTAGCTACCGGATGCTGA	This study	Tag Copenhagen
dFdC_Lead: TCAGCATCCGGTAGCTACTCAATC[C5-Fluro dC]GGTACC	This study	BioSynthesis
AluI-HpaII_dFdC: TCAGCATC[C5-FlurodC]GGTTCGAACTCAATAG[C5-FlurodC]TTACC-3	This study	BioSynthesis
dFdC_Lag: TGAGGTAC[C5-FlurodC]GGATTGAGTAGCTACCGGATGC	This study	BioSynthesis
Reference ladder: 5′-CATTCAGCTCCCGGAGACGGTCACAGCTTG TCTGTAAGCGGATGCCGGGAGCAGACAAGCCCGTCAGGGCGCGT CAGCGGGTGTGGCGGG TGTCGGGGCTGGCTTAACTATGCGGCA TCAGAGCAGATTGTACTGAGAGTGCACCATATGGC TGAGGTACCG	This study	Tag Copenhagen
Sequencing ladder: CATTCAGCTCCCGGAGACGGTC	This study	Tag Copenhagen
Primer A: 5′ – GAT CGG ATC CAT GGA CTA CAA AGA CGA TGA CGA CAA GGG TGA TAT GCA GAT GTC GGT AG – 3′	This study	IDT
Primer B: 5′- GAT CCT CGA GTT ATT ATG TAT TGC AGT TTT GTA AGC AGG TGT CTA AAT G −3′	This study	IDT

**Recombinant DNA**		

pJLS2	This study	N/A
pJLS3	This study	N/A
pNBL104	This study	N/A
pFastBac1-*xl*SPRTN-WT	This study	N/A
pFastBac1- *xl*SPRTN-EQ	This study	N/A
pFastBac1-*xl*SPRTN-SHP^x^	This study	N/A
pFastBac1-*xl*SPRTN-PIP^x^	This study	N/A
pFastBac1-*xl*SPRTN-UBZ^x^	This study	N/A
pFastBac1-*xl*SPRTN-1-435	This study	N/A
pH6-SUMO-*xl*TRAIP-WT	This study	N/A
pH6-SUMO-*xl*TRAIP-R18C	This study	N/A

**Software and Algorithms**		

ImageJ 1.51	NIH	https://imagej.nih.gov/ij
MaxQuant	Cox and Mann, 2008	http://www.coxdocs.org
Perseus 1.5.6.0	Tyanova et al., 2016	http://www.coxdocs.org

### Contact for Reagent and Resource Sharing

Further information and requests for resources and reagents should be directed to and will be fulfilled by the Lead Contact, Julien P. Duxin (julien.duxin@cpr.ku.dk).

### Experimental Model and Subject Details

Egg extracts were prepared using *Xenopus laevis* (Nasco Cat #LM0053MX, LM00715MX). All experiments involving animals were approved by the Harvard Medical Area Institutional Animal Care and Used Committee and by the Danish Animal Experiments Inspectorate, and are conform to relevant regulatory standards and European guidelines.

### Method Details

#### *Xenopus* Egg Extracts and DNA Replication

Preparation of *Xenopus* egg extracts was performed as described previously (Lebofsky et al., 2009, Walter et al., 1998). For high-speed supernatant (HSS) preparation, 6 female frogs (Nasco) were primed by injection with 80 IU of human chorionic gonadotropin (hCG, Sigma). 2-7 days after priming, frogs were injected with 625 IU of hCG and placed in individual tanks containing 100 mM NaCl. 18-20 hours post injection, eggs were collected and used for extract preparation. Eggs were first dejellied in cysteine buffer for 7 min (2.2% cysteine-HCl, pH 7.7), washed 3 times in in 0.5X MMR buffer (final concentration: 50 mM NaCl, 1 mM KCl, 0.25 mM MgSO_4_, 1.25 mM CaCl_2_, 2.5 mM HEPES, 0.05 mM EDTA, pH 7.8) and washed 3 times in ELB sucrose buffer (2.5 mM MgCl_2_, 50 mM KCl, 10 mM HEPES, 250 mM sucrose, 1 mM DTT, 50 μg/mL cyclohexamide, pH 7.8). Eggs were packed for 1 min at 176 x g for 1 min and crushed for 20 min at 20, 000 x g in a swing bucket rotor at 4°C in the presence of cytochalasin B (final concentration: 2.5 μg/mL), aprotinin (final concentration: 5 μg/mL) and leupeptin (final concentration: 5 μg/mL). Crude interphase extract was recovered post-centrifugation and spun in ultracentrifuge for 90 min at 260,000 x g at 2°C following addition of cyclohexamide (final concentration: 50 μg/mL), DTT (final concentration: 1 mM), aprotinin (final concentration: 10 μg/mL), leupeptin (final concentration: 10 μg/mL) and cytochalasin B (final concentration: 5 μg/mL). Following centrifugation, the small lipid layer on top was removed. The soluble HSS was harvested, snap frozen in 33 uL aliquots and stored at −80°C. For nucleoplasmic egg extract (NPE), 20 female frogs were injected and the crude interphase extract was prepared in the same manner than for HSS. Once collected the crude interphase extract was supplemented with cyclohexamide (final concentration: 50 μg/mL), DTT (final concentration: 1 mM), aprotinin (final concentration: 10 μg/mL), leupeptin (final concentration: 10 μg/mL), cytochalasin B (final concentration: 5 μg/mL) and nocadazole (final concentration: 3.3 ug/mL). The extract was spun at 20 000 x g at 4°C for 10 min. The lipid layer on top was removed and the interphase extract decanted to a new tube. The interphase extract was supplemented with ATP (final concentration: 2 mM), phosphocreatine (final concentration: 20 mM) and creatine phosphokinase (final concentration: 5 μg/mL) and nuclear assembly reactions were initiated by adding demembranated sperm chromatin to a final concentration of 4,400/μL. The nuclear assembly reaction was incubated at room temperature for 60-85 min, and then spun for 2 min at 20 000 x g in a swing-bucket rotor. The nuclear layer on top was recovered and then spun in a swinging bucket rotor at 260,000 x g at 2°C for 30 min. Lipids on top were removed and the clear soluble NPE was harvested. 10 μL NPE aliquots were snap-frozen and kept at −80°C.

For DNA replication, plasmids were first incubated in HSS (final concentration: 7.5 ng DNA/μL HSS), supplemented with nocadazole (final concentration: 3 μg/mL) and ATP regeneration mix (final concentration: 20 mM phosphocreatine, 2 mM ATP, 5 μg/mL creatine phosphokinase, for 20-30 min at room temperature to license the DNA. Two volumes of NPE supplemented with 4 mM DTT, 20 mM phosphocreatine, 2 mM ATP, 5 μg/mL creatine phosphokinase were then added to 1 volume of licensing reaction to initiate replication. Where indicated, HSS was supplemented with Geminin at a final concentration of 10 μM and incubated for 10 min at room temperature prior to addition of plasmid DNA. For replication in the presence of LacI, plasmid DNA (75 ng/uL) was incubated with an equal volume of 12 μM LacI for 1 hr prior to HSS addition (Duxin et al., 2014). For UbVS treatment, NPE was supplemented with 22.5 μM ubiquitin vinyl sulfone (UbVS) (Boston Biochem) and incubated for 15 min prior to mixing with HSS (15 μM final concentration). Where indicated, recombinant ubiquitin or FLAG-ubiquitin (Boston Biochem) were added to NPE at a concentration of 120 μM (80 μM final concentration). For SPRTN depletion-rescue experiments, NPE was supplemented with 30 nM recombinant wild-type or mutant *Xenopus* SPRTN. For TRAIP depletion-rescue experiments, NPE was supplemented with 100 nM of recombinant wild-type or R18C *Xenopus* TRAIP. To block *de novo* SUMOylation, dnUBC9 was added to extracts to a final concentration of 10 μM (Azuma et al., 2003). Where indicated, proteasome activity was inhibited via the addition of 200 μM MG262 (Boston Biochem) to extracts (final concentration). For DNA labeling, reactions were supplemented with [α-^32^P]dATP. To analyze plasmid replication intermediates, 1 μL of each reaction was added to 5 μL of replication stop solution A (5% SDS, 80 mM Tris pH 8.0, 0.13% phosphoric acid, 10% Ficoll) supplemented with 1 μL of Proteinase K (20 mg/ml) (Roche). Samples were incubated for 1 hr at 37°C prior to separation by 0.9% native agarose gel electrophoresis and visualization using a phosphorimager (Lebofsky et al., 2009). For analysis of nascent leading strand products, 3-4 μL of each replication reaction was added to 10 volumes of 50 mM Tris pH 7.5, 0.5% SDS, 25 mM EDTA, and replication intermediates were purified by phenol chloroform extraction. For incubation in non-licensing extracts, one volume of HSS and two volumes of NPE were premixed prior to the addition of plasmid DNA (final concentration of 10 ng/μL). Where indicated, aphidicolin (Sigma) and *ara*-cytidine-5′-triphosphate (araCTP) (Jena Bioscience), were added to a final concentration of 700 μM and 1 mM, respectively. All experiments were performed at least in duplicate and a representative experiment is shown. Radioactive signal was quantified using ImageJ (NIH, USA).

#### Preparation of DNA constructs

To generate pDPC we first created pJLS2 by replacing the AatII-BsmBI fragment from pJD2 (Duxin et al., 2014) with the following sequence:

5′-GGGAGCTGAATGCCGCGCGAATAATGGTTTCTTAGACGT-3′ which contains a Nb.BsmI site.

To generate pDPC^2xLead^, the SacI-BssHII fragment from pJLS2 was replaced with the following sequence:

5′CATCCACTAGCCAATTTATGCTGAGGTACCGGATTGAGTAGCTACCGGATGCTGAGGGGATCCACTAGCCAATTTATCATGG-3′.

pJLS2 or pJLS3 were nicked with Nt.BbvcI and ligated with the following oligo containing a fluorinated cytosine: 5′-TCAGCATCCGGTAGCTACTCAATC[C5-Fluro dC]GGTACC-3′ and subsequently crosslinked to M.HpaII-His_6_ to generate pDPC or pDPC^2xLead^, respectively, as previously described (Duxin et al., 2014). To this end, the modified fluorinated DNA was gel purified and mixed with M.HpaII-His_6_ in reaction buffer (50 mM Tris-HCl pH 7.5, 5 mM 2-mercaptoethanol, 10 mM EDTA) supplemented with 100 μM of S-adenosylmethionine (NEB) for 12 hr at 37°C. To generate pDPC^PK^, pDPC was treated with Proteinase K (37°C overnight in presence of 0.5% SDS) to reduce the DPC to a 4 amino acids peptide adduct. The plasmid was subsequently recovered by phenol/chloroform extraction. To generate pDPC^+peptide^ the ApoI-NdeI fragment of pJLS2 was replaced with the following sequence: 5′- AATTCCTCAGCATCCGGTTCGAACTCAATAGCTTACCTCAGCCA-3′, generating pNBL104. pNBL104 was nicked with Nt.BbvCI and ligated with the following oligo containing both a fluorinated AluI site and a fluorinated M.HpaII site: 5′- TCAGCATC[C5-FlurodC]GGTTCGAACTCAATAG[C5-FlurodC]TTACC-3′. AluI Methyltransferase (New England BioLabs) was first crosslinked to the plasmid, degraded with Proteinase K (37°C overnight in presence of 0.5% SDS) and the plasmid was recovered by phenol/chloroform extraction. The peptide-containing plasmid was then crosslinked to M.HpaII-His_6_ as described above. To generate pDPC^ssDNA^, pJLS2 was nicked with Nb.BbvCI and ligated with the following fluorinated oligo: 5′-TGAGGTAC[C5-FlurodC]GGATTGAGTAGCTACCGGATGC-3′. The dFdC-containing plasmid was cut with Nt.BbvCI and the resulting 31bp fragment was melted off and captured by annealing to an excess complimentary oligo 5′-GGTACCGGATTGAGTAGCTACCGGATGCTGA-3′. Excess oligos were then degraded by Exonuclease I (New England BioLabs) treatment. The gapped plasmid was then recovered by phenol/chloroform extraction and crosslinked to M.HpaII-His_6_ as described above.

#### Antibodies and Immunodepletion

The following antibodies used were described previously: REV1 (Budzowska et al., 2015), ORC2 (Fang and Newport, 1993). M.HpaII antibody was raised against full length M.HpaII-His_6_ expressed and purified from bacteria under denaturing conditions (Pocono Rabbit Farm & Laboratory). PSMA1, PSMA3, SPRTN, TRAIP and MCM6 antibodies were raised by New England Peptide by immunizing rabbits with Ac-CAEEPVEKQEEPMEH-OH, Ac-CKYAKESLEEEDDSDDDNM-OH, Aoa-DVLQDKINDHLDTCLQNCNT-OH, Ac-CTSSLANQPRLEDFLK-OH and Ac-CLVVNPNYMLED-OH, respectively. SPRTN-N antibody was raised against a fragment of *Xenopus laevis* SPRTN encompassing amino acids 67-287 which was tagged on N terminus with His_6_. The protein fragment was purified from bacteria under denaturing conditions and the antibody was raised by Pocono Rabbit Farm & Laboratory. Western blotting analysis for H3 was carried out with commercial antibody from Cell Signaling (Cat #9715S).

To immunodeplete SPRTN from *Xenopus* egg extracts, one volume of Protein A Sepharose Fast Flow (PAS) (GE Health Care) was mixed with 4 volumes of affinity purified SPRTN peptide antibody (1 mg/mL) and incubated overnight at 4°C. The beads were then washed twice with 500 μL PBS, once with ELB (10 mM HEPES pH 7.7, 50 mM KCl, 2.5 mM MgCl_2_, and 250 mM sucrose), three times with ELB supplemented with 0.5 M NaCl, and twice with ELB. One volume of precleared HSS or NPE was then depleted by mixing with 0.2 volumes of antibody-bound beads then incubating at room temperature for 20 min. The depletion procedure was repeated once. To immunodeplete PSMA1, one volume of PAS beads was mixed with 10 volumes of affinity purified PSMA1 peptide antibody (1 mg/mL). The beads were washed as described above, and one volume of precleared HSS or NPE was then depleted by mixing with 0.2 volumes of antibody-bound beads and then incubating at room temperature for 20 min. The depletion procedure was repeated three times for HSS and twice for NPE. For SPRTN and PSMA1 combined depletion, one volume of PAS beads was mixed with 4 volumes of affinity purified SPRTN peptide antibody and 10 volumes of affinity purified PSMA1 peptide antibody. The beads were washed and depletion was performed as described for PSMA1 immunodepletion. The immunodepletion of REV1 was performed as previously described (Budzowska et al., 2015). To immunodeplete TRAIP, one volume of Protein A Sepharose Fast Flow (PAS) (GE Health Care) was mixed with 2.5 volumes of affinity purified TRAIP antibody (1 mg/mL) and incubated overnight at 4°C. The beads were washed as described above, and one volume of precleared HSS or NPE was then depleted by mixing with 0.2 volumes of antibody-bound beads and then incubating at room temperature for 20 min. The depletion procedure was repeated twice for HSS and twice for NPE. Experiments in Figures 7 and S7E–S7G were performed using the SPRTN-N antibody where one volume of PAS was mixed with 3 volumes of SPRTN-N serum and incubated overnight at 4°C. The beads were washed as described above, and one volume of precleared HSS or NPE was then depleted by mixing with 0.2 volumes of antibody-bound beads and then incubating at room temperature for 20 min. The depletion procedure was repeated once.

#### Nascent-Strand Analysis

Nascent strand analysis was performed as previously described (Räschle et al., 2008). Briefly, purified DNA was digested with the indicated restriction enzymes followed by addition of 0.5 volumes of Gel Loading Dye II (Denaturing PAGE) (Life Technologies). DNA fragments were subsequently separated on 5% or 7% denaturing polyacrylamide gels, transferred to filter paper, dried, and visualized using a phosphorimager. Radioactive signal was quantified using ImageJ (NIH, USA).

Reference oligo used in Figure S4I: 5′-CATTCAGCTCCCGGAGACGGTCACAGCTTG TCTGTAAGCGGATGCCGGGAGCAGACAAGCCCGTCAGGGCGCGTCAGCGGGTGTGGCGGGTGTCGGGGCTGGCTTAACTATGCGGCATCAGAGCAGATTGTACTGAGAGTGCACCATATGGCTGAGGTACCG-3′.

Primer used for dideoxy-sequencing ladder in Figure S4I: 5′- CAT TCA GCT CCC GGA GAC GGT C – 3′.

#### Protein Expression and Purification

M.HpaII-His_6_ was expressed and purified as previously described (Duxin et al., 2014). Briefly, pHpaII-Avitag-His_6_ was transformed in T7 Express Competent E.coli cells (NEB), cells cultured in the presence of 100 μg/mL ampicillin until the OD_600_ reached 0.7. The culture was supplemented with 0.5 mM IPTG for 3 hours, collected by centrifugation and resuspended in 15 mL Lysis Buffer (20 mM Tris pH 8.5, 500 mM KCl, 10% glycerol, 10 mM imidazole and protease inhibitors (Roche)). Cells were lysed by sonication and cleared by centrifugation at 20, 000 x g for 30 min. Cleared lysate was applied onto Ni-NTA resin (QAGEN). The resin was washed with 25 mL of Lysis Buffer containing 30 mM imidazole and the protein eluted with Elution Buffer (20 mM Tris pH 8.5, 100 mM KCl, 10% glycerol and 250 mM imidazole). Eluate was dialyzed overnight in Storage Buffer (20 mM Tris pH 8.5, 100 mM KCl, 1 mM DTT, 30% glycerol) and protein aliquots snap frozen and kept at −80°C. To generate lysine-methylated M.HpaII, purified M.HpaII-His_6_ was first denatured by dialyzing against 20 mM HEPES pH 7.5, 100 mM KCl, 6M Guanidine HCl, 10% glycerol. Denatured M.HpaII protein was then methylated using Reductive Alkylation Kit (Hampton Research) via the addition of dimethylamine borane and formaldehyde according to the manufacturer’s protocols. The methylation reaction was stopped by addition of 100 mM Tris pH 7.5 and 5 mM DTT (final concentrations). Methylated M.HpaII was then renatured by sequentially dialyzing against Renaturing Buffer (20 mM Tris pH 8.5, 100mM KCl, 1mM DTT, 10% glycerol) supplemented with 4, 2, and 0 M Guanidine HCl for 1 hr each at 4°C. The renatured protein was then dialyzed against storage buffer (20 mM Tris pH 8.5, 100 mM KCl, 1 mM DTT, 30% glycerol) and stored at −80°C.

LacI-biotin protein was purified from T7 Express Competent cells (NEP) (Duxin et al., 2014). Briefly, pET11a-LacI and pBirAcm (Avidity) were co-transformed and cells cultured in the presence of 100 μg/mL ampicillin and 34 μg/mL chloramphenicol at 37°C until OD_600_ reached 0.6. The culture was supplemented with 1 mM IPTG and 50 μM biotin for 2 hours. Cells were collected by centrifugation and resuspended in Buffer 1 (50 mM Tris pH 7.5, 5 mM EDTA, 100 mM NaCl, 10% sucrose, 1 mM DTT, protease inhibitors (Roche), 0.2 mg/mL lysozyme (Sigma), 0.1% Brij 58) and rotated for 30 min at room temperature. The cell lysate was pelleted by centrifugation for 60 min at 20, 000 x g and the insoluble pellet was resuspended in 10 mL of Extraction Buffer (50 mM Tris pH 7.5, 5 mM EDTA, 1M NaCl, 30 mM IPTG, 1 mM DTT and protease inhibitors). The resuspended pellet was homogenized by sonication and pelleted again for 60 min at 20, 000 x g. The supernatant was collected and 1% polymin P was added to 0.045%. Lysate was rotated for 30 min at 4°C and pelleted at 20, 000 x g for 20 min. The supernatant was transferred to a new tube and ammonium sulfate was added to a final saturation of 37% followed by rotation for 30 min at 4°C. The pellet was recovered and resuspended in 2 mL of Wash Buffer (50 mM Tris pH 7.5, 1 mM EDTA, 100 mM NaCl, 1 mM DTT and protease inhibitors). The resuspension was applied to a column containin 1 mL of softlink avidin resin and inbutated for 1 hour at 4°C. The column was washed with 15 mL of Wash Buffer, and the protein eluted with Elution buffer (50 mM Tris pH 7.5, 1 mM EDTA, 100 mM NaCl, 1 mM DTT and 5 mM biotin). Protein was dialyzed overnight with Dialysis Buffer (50 mM Tris pH 7.5, 1 mM EDTA, 150 mM NaCl, 1 mM DTT and 30% glycerol) and stored at −80°C.

*Xenopus* SPRTN with an N-terminal FLAG tag was cloned into pFastBac1 (Thermo Fisher Scientific) using primers A and B. SPRTN mutations were introduced via Quikchange mutagenesis and confirmed by Sanger sequencing. SPRTN Baculoviruses were prepared using the Bac-to-Bac system (Thermo Fisher Scientific) according to the manufacturer’s protocols. SPRTN was expressed in 250 mL suspension cultures of Sf9 insect cells (Thermo Fisher Scientific) by infection with SPRTN baculovirus for 48 hr. Sf9 cells were subsequently collected via centrifugation and resuspended in Lysis Buffer (50 mM Tris pH 7.5, 500 mM NaCl, 10% Glycerol, 1X Roche EDTA-free Complete protease inhibitor cocktail, 0.5 mM PMSF, 0.2% Triton X-100). To lyse cells, the suspension was subjected to three freeze/thaw cycles, passed through a 21 g needle, and then sonicated. The cell lysate was spun at 25000 rpm in a Beckman SW41 rotor for 1hr. The soluble fraction was collected and then incubated with 200 μL anti-FLAG M2 affinity resin (Sigma) for 90 min at 4°C. The resin was then washed once with 10 mL Lysis Buffer, twice with Wash Buffer (50 mM Tris pH 7.5, 500 mM NaCl, 10% Glycerol, 0.2% Triton X-100), and three times with Buffer A (50 mM Tris pH 7.5, 500 mM NaCl, 10% Glycerol). FLAG-SPRTN was eluted with Buffer A supplemented with 100 μg/mL 3xFLAG peptide (Sigma). Elution fractions containing FLAG-SPRTN protein were pooled and dialyzed against 20 mM Tris pH 7.5, 300 mM NaCl, 10% Glycerol, 1mM DTT at 4°C for 12 hr and then dialyzed against Storage Buffer (20 mM Tris pH 7.5, 150 mM NaCl, 10% Glycerol, 1mM DTT) at 4°C for 3 hr. Aliquots of FLAG-SPRTN were then stored at −80°C.

*Xenopus* recombinant TRAIP wild-type (WT) and TRAIP R18C were expressed and purified with a 6xHis-SUMO tag in bacteria. Briefly, Rosetta 2 (DE3) pLysS competent cells (Novagen) were transformed with pH_6_-SUMO-TRAIP WT or pH_6_-SUMO TRAIP R18C and cells grown in the presence of 100 μg/mL ampicillin and 27 μg/mL chloramphenicol at 37°C until OD_600_ reached 0.6. Cells were then transferred to 16°C for 30 min and supplemented with 0.1 mM IPTG and 50 μM ZnSO_4_ overnight. The culture was collected by centrifugation and resuspended in Lysis Buffer (20 mM HEPES pH 7.5, 400 mM sodium acetate, 10% glycerol, 20 mM imidazole, 10 μM ZnSO_4_, 0.1% NP-40, 1 mM DTT and protease inhibitors). The lysate was sonicated, and ammonium sulfate and polyethyleneimine were added to final concentrations of 300 mM and 0.45%, respectively, and incubated for 15 min at 4°C. The lysate was centrifuged at 40, 000 x g for 45 min and the soluble fraction recovered and precipitated with ammonium sulfate. The precipitated fraction was collected by centrifugation at 40,000 x g for 45 min and resuspended in Lysis Buffer and rotated for 30 min with NiNTA resin at room temperature. The resin was washed three times with Wash Buffer (20 mM HEPES pH 7.5, 400 mM sodium acetate, 10% glycerol, 20 mM imidazole, 10 μM ZnSO_4_, 0.01% NP-40, 1 mM DTT and protease inhibitors) and the protein was eluted from resin with Elution Buffer (20 mM HEPES pH 7.5, 400 mM sodium acetate, 10% glycerol, 120 mM imidazole, 10 uM ZnSO_4_, 0.01% NP-40, 1 mM DTT). The eluate was then dialized with Dialysis Buffer (20 mM HEPES pH 7.5, 400 mM sodium acetate, 120 mM imidazole, 10% glycerol) overnight at 4°C in the presence of 0.03 mg/mL Ulp1. Aliquots were flash frozen and stored at −80°C.

*Xenopus* recombinant 6xHis-Geminin was expressed and purified as previously described (McGarry and Kirschner, 1998). Briefly, BL21 cells were transformed with pET28a-His-Geminin and cultured until the OD_600_ reached 0.6. The culture was supplemented with 0.5 mM IPTG for 3 hours, collected by centrifugation and resuspended in 10 mL Buffer S (50 mM NaPi pH 7.6, 5 mM BME, 1 mM PMSF and 2 mM benzamidine) containing 10 mg lysozyme and 1% Triton X-100. Cells were incubated for 10 min at room temperature and supplemented with 1 mL of 5M NaCl. Cells were then sonicated and pelleted at at 20, 000 x g for 30 min. Cleared lysate was supplemented with 20 mM imidazole and applied onto Ni-NTA resin (QAGEN) for 1 hour at 4C. The resin was washed with Buffer W (50 mM NaPi pH 7.6, 0.5 M NaCl, 0.1% Triton X-100, 5 mM BME, 20 mM imidazole), and the protein eluted with Elution Buffer (50 mM NaPi pH 7.6, 0.5M NaCl, 5 mM BME). The protein was dialyzed overnight with (10 mM Tris pH 8, 0.5M NaCl and 5% glycerol). Aliquots were flash frozen and stored at −80°C.

Primer A: 5′ – GAT CGG ATC CAT GGA CTA CAA AGA CGA TGA CGA CAA GGG TGA TAT GCA GAT GTC GGT AG – 3′

Primer B: 5′- GAT CCT CGA GTT ATT ATG TAT TGC AGT TTT GTA AGC AGG TGT CTA AAT G −3′

#### Plasmid Pull-Downs

Plasmid pull-down assays were performed as previously described (Budzowska et al., 2015). 6 μL/pull down of streptavidin-coupled beads (Dynabeads M-280, Invitrogen) were washed three times with wash buffer 1 (50 mM Tris pH7.5, 150 mM NaCl, 1 mM EDTA pH 8, 0.02% Tween-20). Biotinylated LacI was added to the beads at 12 pmol/6 μL of beads, and incubated at RT for 1 hour. The beads were washed four times with pull-down buffer (10 mM HEPES pH 7.7, 50 mM KCl, 2.5 mM MgCl_2_, 250 mM sucrose, 0.25 mg/mL BSA, 0.02% Tween-20) and resuspended in 40 μL of the same buffer and stored on ice. At the indicated time point, 6 to 8 μL of reaction samples were withdrawn and gently mixed with the beads. The suspension was rotated for 30 min at 4°C. The beads were then washed 2 times with wash buffer 2 (10 mM HEPES pH 7.7, 50 mM KCl, 2.5 mM MgCl2, 0.25 mg/mL BSA and 0.03% Tween-20). After washing, beads were resuspended in 40 μL of 2x Laemmli sample buffer and equal volume of protein samples were resolved on SDS-PAGE gels. Proteins associated with the chromatin fraction were visualized by western blotting with the indicated antibodies and developed using the chemiluminescence function on Amersham Imager 600 (GE Healthcare).

#### DPC Pull-Downs

We developed a modified plasmid pull-down protocol to specifically isolate M.HpaII DPCs from extracts (Figure 1A). Streptavidin-coupled magnetic beads (Dynabeads M-280, Invitrogen; 5 μL per pull-down) were washed twice with 50 mM Tris pH 7.5, 150mM NaCl, 1mM EDTA pH 8, 0.02% Tween-20. Biotinylated LacI was added to the beads (1 pmol per 5 μL of beads) and incubated at room temperature for 40 min. The beads were then washed four times with DPC pull-down buffer (20 mM Tris pH 7.5, 150 mM NaCl, 2 mM EDTA pH 8, 0.5% IPEGAL-CA630) and then stored in the same buffer on ice until needed. At the indicated times during DNA replication or gap filling, equal volumes (2-10 μL) of reaction were withdrawn and stopped in 300 μL of DPC pull-down buffer on ice. After all of the time points were taken, 5 μL of LacI-coated streptavidin Dynabeads were added to each sample and allowed to bind for 30-60 min at 4°C rotating. 20 μL of pull-down supernatant was mixed with 20 μL of 2X Laemmli sample buffer for input. The beads were subsequently washed four times with DPC pull-down buffer and then twice with Benzonase buffer (20 mM Tris pH 7.5, 150 mM NaCl, 2 mM MgCl_2_, 0.02% Tween-20) before being resuspended in 15 μL Benzonase buffer containing 1 μL Benzonase (Novagen). Samples were incubated for 1hr at 37°C to allow for DNA digestion and DPC elution, after which the beads were pelleted and the supernatant M.HpaII eluate was mixed with 2X Laemmli sample buffer for subsequent western blotting analysis.

For FLAG immunoprecipitation analysis of isolated DPCs (Figure 1D), the M.HpaII eluate resulting from Benzonase treatment was instead diluted to 300 μL in Benzonase buffer. FLAG M2 magnetic beads (Invitrogen; 5 μL per pull-down) were added to each sample and allowed to bind for 60 min at 4°C rotating. The beads were subsequently washed four times with Benzonase buffer. To elute precipitated proteins, the beads were then resuspended in 0.1 M Glycine pH 3 and incubated with gentle shaking for 10 min at room temperature. After pelleting the beads, the supernatant was neutralized with 10mM Tris pH 11 and mixed with 2X Laemmli buffer.

For UbiCREST analysis of isolated DPCs (Figures S1E and S1F), pull down samples were washed with DPC pull-down buffer as described initially but were instead then washed and resuspended in 1X DUB reaction buffer and treated with the indicated deubiquitinase(s) (Boston Biochem; Hospenthal et al., 2015) at 37°C for 30 min. The samples were subsequently washed twice with Benzonase buffer and eluted with Benzonase treatment as previously described.

To monitor M.HpaII degradation pDPC^Lead^ or pDPC^Lag^ plasmids were pre-bound with purified LacI (untagged) for 60 min at RT as previously described (Duxin et al., 2014). Pre-bound plasmids were replicated at 5 ng/μL final concentration in HSS/NPE, and reactions stopped in DPC pull-down buffer. DPC plasmids were pulled down washed and benzonase treated as described above. After elution with benzonase, the eluates were incubated with His-tag dynabeads to recover M.HpaII-His_6_ DPCs (Life Technologies) in HIS wash buffer (50 mM sodium phosphate buffer, pH8, 150 mM NaCl, 0.02% Tween-20) for 10 minutes at 4°C. This step was added to avoid cross reactivity between free LacI and the M.HpaII antibody. Beads were washed three times in HIS wash buffer and eluted in HIS elution buffer (300 mM imidazole, 50 mM sodium phosphate buffer, pH8, 300 mM NaCl, 0.01% Tween20) shaking at RT for 5 min. The supernatant M.HpaII-His_6_ eluate was mixed with 2X Laemmli sample buffer for subsequent western blotting analysis.

#### Plasmid Pull-down Mass Spectrometry (PP-MS)

Plasmid DNA was replicated in egg extracts at 5 ng/μL (final concentration). At the indicated time points, 8 μL of the reaction were withdrawn and plasmids and associated proteins were recovered by plasmid pull down using LacI coated beads (Budzowska et al., 2015). After 30 min incubation at 4°C, samples were washed twice in 10 mM HEPES pH 7.7, 50 mM KCl, 2.5 mM MgCl_2_, 0.03% Tween 20, and once in 10 mM HEPES pH 7.7, 50 mM KCl, 2.5 mM MgCl_2_. Samples were washed one additional time in 50 μL of 10 mM HEPES pH 7.7, 50 mM KCl, 2.5 mM MgCl_2_ and transferred to a new tube to remove residual detergent. Beads were dried out and resuspended in 50 μL denaturation buffer (8 M Urea, 100 mM Tris pH 8.0). Cysteines were reduced (1 mM DTT, 15 minutes at RT) and alkylated (5 mM iodoacetamide, 45 min at RT). Proteins were digested and eluted from beads with 1.5 μg LysC (Sigma) for 2.5 hr at RT. Eluted samples were transferred to a new tube and diluted 1:4 with ABC (50 mM ammonium bicarbonate). 2.5 μg trypsin was added and incubated for 16 hours at 30°C. NaCl was added to 400 mM final concentration, and peptides were acidified and purified by stage tipping on C18 material. Samples were analyzed on a Q Exactive HF Orbitrap mass spectrometer (Thermo Scientific) and quantified by the label free algorithm implemented in the MaxQuant software, as previously described (Räschle et al., 2015). MS experiments were carried out in quadruplicates. A fifth replicate was used to isolate the DNA repair intermediates shown in Figure 2A. The mass spectrometry data have been deposited to the ProteomeXchange repository with the dataset identifier PXD008831.

### Quantification and Statistical Analysis

All bioinformatics analysis was carried out with the Perseus software Version 1.5.6.0. For each comparison, the processed data was filtered to contain at least 3 valid values in at least one of the replicate group. A modified, one-sided T-Test implemented in Perseus (Tyanova et al., 2016) was carried out using a False Discovery Rate (FDR) cut-off of 0.01 and S0 = 4. Autoradiographs were quantified using ImageJ. Error bars represent standard deviations.

### Data and Software Availability

Perseus is provided by the group of Jürgen Cox at the MPI of Biochemistry and can be freely downloaded at: http://www.coxdocs.org.

## Supplemental Information

Document S1. Figures S1–S7

Table S1. Protein Enrichment on pDPC^2xLead^; Related to Figure 2 The table summarizes the results from pairwise comparisons using a modified t test with a permutation-based FDR cut-off implemented in the Perseus frame work (Tyanova et al., 2016). To test for protein enrichment on plasmid substrates a one-sided t-Test was carried out with a FDR < 0.01 and S0 = 4. For each protein in column A significant enrichment is indicated in column E-K (1: significant, 0: not significant, NA: not detected or not enough valid values). Column Al-CQ list the log2 fold change (columns labeled DIFF) and the associated –LOG_10_(p value) (columns labeled PVAL). All experiments were measured in quadruplicates. The combined p value is the product of individual p values across the time series corrected by random permutation across the entire data matrix (see (Tyanova et al., 2016) for details). pDPC, plasmid substrate crosslinked with M.HpaII at both leading strand templates (pDPC^2xLead^) (see Figure 2); pCTR, undamaged control plasmid (pCTRL); Gem, reactions containing the replication inhibitor geminin; UB-VS, reactions containing ubiquitin vinyl sulfone.

Table S2. Dynamic Recruitment of DNA Repair Factors to pDPC^2xLead^; Related to Figure 2 The table shows the z-scored log2 LFQ intensities (mean from 4 biochemical replicates) for all quantified proteins (column A-O). A subjective chronological order for selected proteins is provided (column Q).

Document S2. Article plus Supplemental Information
