# Supplementary material for: Replication-Coupled DNA-Protein Crosslink Repair by SPRTN and the Proteasome in Xenopus Egg Extracts
Source: Mol Cell. 2019 Feb 7;73(3):574–588.e7. doi: 10.1016/j.molcel.2018.11.024 (PMC6375733; doi:10.1016/j.molcel.2018.11.024)
Supplement: Document S1. Figures S1–S7 [file mmc1.pdf]

Molecular Cell, Volume *73*

Supplemental Information

Replication-Coupled DNA-Protein Crosslink Repair

by SPRTN and the Proteasome in *Xenopus* Egg Extracts

Nicolai B. Larsen, Alan O. Gao, Justin L. Sparks, Irene Gallina, R. Alex Wu, Matthias Mann, Markus Räschle, Johannes C. Walter, and Julien P. Duxin

Supplemental Figure Legends

Figure S1. Replication-coupled DPC ubiquitylation and replication-independent DPC SUMOylation. Related to Figure 1. (**A**) Schematic illustrating replication intermediates generated during replication of pDPC (Duxin et al., 2014). (**B**) pDPC was replicated in egg extracts in the presence of [α-^{32}P]dATP. Geminin (+Gem.) was supplemented where indicated to block DNA replication (Tada et al., 2001; Wohlschlegel et al., 2000). Samples were analyzed by agarose gel electrophoresis. RI, replication intermediates; OC, open circular; SC, supercoiled. (**C**) pDPC^{2xLead} was incubated in egg extracts that do not support CMG licensing and replication initiation. Recombinant UBC9 dominant-negative (+dnUBC9) was added where indicated to block SUMOylation (Azuma et al., 2003). DPCs were recovered and monitored as in Figure 1B. * indicates residual uncrosslinked M.HpaII. (**D**) pDPC^{2xLead} was replicated in egg extracts supplemented with buffer (+buf) or UBC9 dominant-negative (+dnUBC9). DPCs were recovered and monitored as in Figure 1B. (**E**) pDPC^{2xLead} was replicated in egg extracts. At 15 minutes, when most DPCs are polyubiquitylated, DPCs were recovered and subsequently treated with the indicated deubiquitinating enzyme (DUB) (Hospenthal et al. 2015). DPCs were monitored as in Figure 1B with a M.HpaII antibody. A red dashed line was added to compare the length of ubiquitin chains between the different treatments. (**F**) pDPC^{2xLead} was replicated and DPCs monitored as in (E). DPCs were treated with the indicated combinations of DUBs prior to electrophoresis. (**G**) pDPC^{2xLead} was replicated in egg extracts in the presence of buffer (+buf), 15 μM of ubiquitin-vinyl-sulfone (UbVS), or 15 μM of UbVS and 80 μM of recombinant ubiquitin. DPCs were recovered and monitored as in Figure 1B.

Figure S2. Validation of PP-MS. (**A**) Analysis of protein recruitment to pCTRL at 10 min compared to pCTRL at 40 min (x axis) and pCTRL at 10 min compared to pCTRL + Geminin at 10 min (y axis). The plot shows the mean difference of the protein intensity of 4 biochemical replicates for each of the conditions indicated. (**B**) Analysis of protein recruitment to pCTRL compared to pCTRL + Geminin at 10 min. The volcano plot shows the mean difference of the protein intensity plotted against the *P* value of 4 biochemical replicates. (**C**) Samples from Figure 2A (autoradiograph) were also digested with FspI and AatII and nascent strand separated on a denaturing polyacrylamide gel. The schematic on top depicts the expected species of nascent leading strands and extension products liberated by FspI and AatII digestion of pDPC^{2xLead} replication intermediates (green hexamer, CMG helicase; red lines, nascent DNA). The locations of the corresponding bands on the gel are indicated by brackets. (**D**) pDPC^{2xLead} and pCTRL were replicated in egg extracts and

recovered at the indicated time point as depicted in Figure 2A (Budzowska et al., 2015). Samples were blotted with the indicated antibodies. Note that this experiment was performed with a different extract exhibiting faster replication kinetics than the one used for MS analysis. (**E**) Analysis of protein recruitment to pDPC^{2xLead} at 40 min compared to pCTRL at 10 min (x axis) and pDPC^{2xLead} at 40 min compared to pDPC^{2xLead} + Geminin at 12 min (y axis). The plot shows the mean difference of the protein intensity of 4 biochemical replicates for each of the conditions indicated.

Figure S3. SPRTN and the proteasome degrade DPCs. Related to Figure 3. (**A**) Mock-depleted and SPRTN-depleted egg extracts were used to replicate pCTRL in the presence of [α-^{32}P]dATP. 200 *μ*M MG262 was added where indicated. Samples were analyzed by native agarose gel electrophoresis as in Figure 3B. The quantification of a representative biological replicate is shown. (**B**) and (**C**) Biological replicates of Figure 3C. (**D**) Mock-depleted, PSMA1-depleted, SPRTN-depleted or PSMA1-SPRTN-depleted extracts were blotted against SPRTN and two proteasome subunits (PSMA1 and PSMA3). ORC2 was blotted as loading control. (**E**) Extracts from (D) were used to replicate pDPC^{2xLead}. DPCs were recovered and monitored as in Figure 1B.

Figure S4. SPRTN-mediated DPC proteolysis facilitates TLS. Related to Figure 4. (**A**) pme-DPC^{2xLead} was replicated in egg extracts supplemented with either DMSO (Mock) or 200 *μ*M of MG262 and analyzed as in Figure 3B. The quantification of a representative biological replicate is shown. (**B**) Samples from (A) were used to monitor DPC degradation as in Figure 1B using anti-M.HpaII antibody. Two different exposures are shown as indicated. (**C**) Schematic of human and *Xenopus laevis* SPRTN protein. Note that *Xenopus laevis* SPRTN contains a duplication of the C-terminal UBZ domain. (**D**) Alignment of the different functional motifs of human and *Xenopus laevis* SPRTN. Residues mutated to generate EQ, SHP*, PIP* and UBZ* are indicated. (**E**) Mock-depleted and SPRTN-depleted extracts were blotted against SPRTN or MCM6 (loading control). SPRTN-depleted extracts were supplemented with either buffer (+buf), or the indicated recombinant FLAG-SPRTN variants. (**F**) Extracts from (D) were used to replicate pme-DPC^{2xLead} in the presence of [α-^{32}P]dATP. Samples were analyzed by agarose gel electrophoresis as in Figure 3B and quantified for open circular (OC) and supercoiled (SC) products. The quantification of a representative biological replicate is shown. (**G**) Mock-depleted and SPRTN-depleted extracts were used to replicate pme-DPC^{2xLead}. SPRTN-depleted extracts were supplemented with either buffer (+buf), or recombinant FLAG-SPRTN variants, as indicated. Samples were analyzed by agarose gel electrophoresis as in Figure 3B and quantified for open circular and supercoiled products. The quantification of a representative biological replicate is shown. (**H**) Samples from (G) were used to monitor SPRTN-

mediated DPC degradation as in Figure 4E. (**I**) Nascent strand intermediates stall at -1, 0 and +1 and not at 0, +1 and +2 as previously reported (Duxin et al., 2014). 30 min samples of pDPC were analyzed by denaturing polyacrylamide gel electrophoresis following nb.BsmI digest alongside a reference oligo and a sequencing ladder generated with ddCTP and ddGTP. In lane 3, the 30 min sample of pDPC was premixed with the reference oligo before electrophoresis. In lanes 5 and 7, the reference oligo was premixed with the cytosine (ddCTP) and guanine (ddGTP) samples of the sequencing ladder, respectively. Note that the middle band of the three discrete bands generated during replication of pDPC aligns with the reference oligo (0 position). Note also that the sequencing ladder alignment with the reference oligo is shifted 1 nucleotide (lanes 4-8). This is likely caused by a faster migration of the sequencing ladder induced by the terminal dideoxynucleotide. In Duxin et al. 2014, the nascent strand stalling positions were determined by comparing pDPC to a sequencing ladder generated by dideoxy sequencing causing the incorrect assignment of the stalling intermediates. (**J**) Mock-depleted and SPRTN-depleted egg extracts were used to replicate pDPC in the presence of [α-^{32}P]dATP. SPRTN-depleted extracts were supplemented with either buffer (+buf), recombinant FLAG-SPRTN (+WT), or recombinant catalytically inactive FLAG-SPRTN E89Q (+EQ). Samples were analyzed by native agarose gel electrophoresis as in Figure 3B. Red arrowheads indicate accumulation of OC repair intermediates. Error bars represent standard deviation of 3 independent experiments. (**K**) Samples from (I) were digested with FspI and AatII and separated on a denaturing polyacrylamide gel and quantified as in Figure 4G. The quantification of a representative biological replicate is shown. (**L**) Depiction of pDPC^{PK} generation. (**M**) Mock-depleted and SPRTN-depleted egg extracts were used to replicate pDPC and pDPC^{PK} in the presence of [α-^{32}P]dATP. Samples were digested with FspI and AatII and separated on a denaturing polyacrylamide gel and quantified as in Figure 4G. The quantification of a representative biological replicate is shown. (**N**) Mock-depleted and REV1-depleted egg extracts were used to replicate pDPC and pDPC^{PK}. Samples were digested with NcoI and AatII and separated on a polyacrylamide denaturing gel as in Figure 4G. The quantification of a representative biological replicate is shown. Note that the plasmid used in this experiment contains the DPC on the bottom strand (pDPC^{Bot} in (Duxin et al., 2014)).

Figure S5. DPC ubiquitylation and degradation can occur in the absence of the replisome. Related to Figure 5. (**A**) and (**B**) Biological replicates of Figure 5C. (**C**) pDPC^{ssDNA} was incubated in mock-depleted, PSMA1-depleted, SPRTN-depleted or PSMA1-SPRTN-depleted non-licensing extracts. DPCs were recovered and monitored with a M.HpaII antibody as in Figure 1B. * indicates residual uncrosslinked M.HpaII. (**D**) Schematic representation of gap filling synthesis when

pDPC^{ssDNA} is incubated in non-licensing extracts (non-replicating). (**E**) pDPC or pDPC^{ssDNA} were incubated in non-licensing egg extracts in the presence of [α-^{32}P]dATP. Samples were analyzed by agarose gel electrophoresis as in Figure 3B. (**F**) Samples from (E) were digested with PvuII and NdeI and separated on a denaturing polyacrylamide gel (bottom autoradiograph). The different extension products are depicted in the upper scheme. (**G**) pDPC^{ssDNA} or pme-DPC^{ssDNA} were incubated in non-licensing egg extracts. DPCs were recovered and monitored as in Figure 1B. Samples at time 0 were withdrawn prior to incubating plasmids in egg extracts and thus lack input signal.

Figure S6. SPRTN-, but not proteasome-dependent DPC degradation requires nascent strand extension to the lesion. Related to Figure 6. (**A**) Depiction of aphidicolin and AraCTP addition after 3 min of incubation of pme-DPC^{ssDNA} in non-licensing extracts. (**B**) pme-DPC^{ssDNA} was incubated in non-licensing extracts. At 3 min extracts were supplemented with 700 μM aphidicolin and 1 mM araCTP where indicated. Samples were analyzed by agarose gel electrophoresis as in Figure 3B. (**C**) Samples from (E) were digested with PvuII and NdeI and separated on a denaturing polyacrylamide gel (bottom autoradiograph). The different extension products are depicted in the upper scheme. (**D**) DPCs from (B) were recovered and monitored as in Figure 1B. (**E**) Depiction of pDPC$^{+peptide}$ replication in REV1-depleted extracts. (**F**) pDPC$^{+peptide}$ was replicated in REV1 depleted extracts supplemented with either DMSO or 200 μM MG262 where indicated. DNA was digested with nb.BsmI and nascent leading strands were then separated on a polyacrylamide denaturing gel as in Figure 6F. (**G**) Samples from (F) were used to monitor DPC degradation as in Figure 1B. * indicates residual uncrosslinked M.HpaII.

Figure S7. TRAIP ubiquitin ligase stimulates replication-coupled DPC ubiquitylation. Related to Figure 7. (**A**) pDPC^{2xLead} was replicated in SPRTN-depleted extracts that were also either Mock-depleted or TRAIP-depleted. DPCs were recovered and monitored as in Figure 1B. (**B**) Biological replicate of experiment in (A). (**C**) Extracts described in (A) were used to replicate pDPC^{2xLead} in the presence of [α-^{32}P]dATP. Samples were analyzed by native agarose gel electrophoresis as in Figure 3B. Error bars represent the standard deviation of 3 independent experiments. (**D**) pDPC^{ssDNA} was incubated in non-licensing extracts that were SPRTN-depleted and either Mock-depleted or TRAIP-depleted. DPCs were recovered and monitored as in Figure 1B. * indicates residual uncrosslinked M.HpaII. (**E**) pDPC^{2xLead} was replicated in SPRTN-depleted extracts that were either Mock-depleted,

RTEL1-depleted, TRAIP-depleted, or RTEL1 and TRAIP co-depleted. DPCs were recovered and monitored as in Figure 1B. (**F**) Samples from Figure 7D were replicated in the presence of [α-^{32}P]dATP. Samples were analyzed by native agarose gel electrophoresis as in Figure 3B. The quantification of a representative biological replicate is shown. (**G**) pme-DPC^{2xLead} was replicated in Mock or TRAIP-depleted extracts in the presence of [α-^{32}P]dATP and nascent strand intermediates were analyzed as in Figure 4G. Error bars represent the standard deviation of 3 independent experiments.

Figure S1

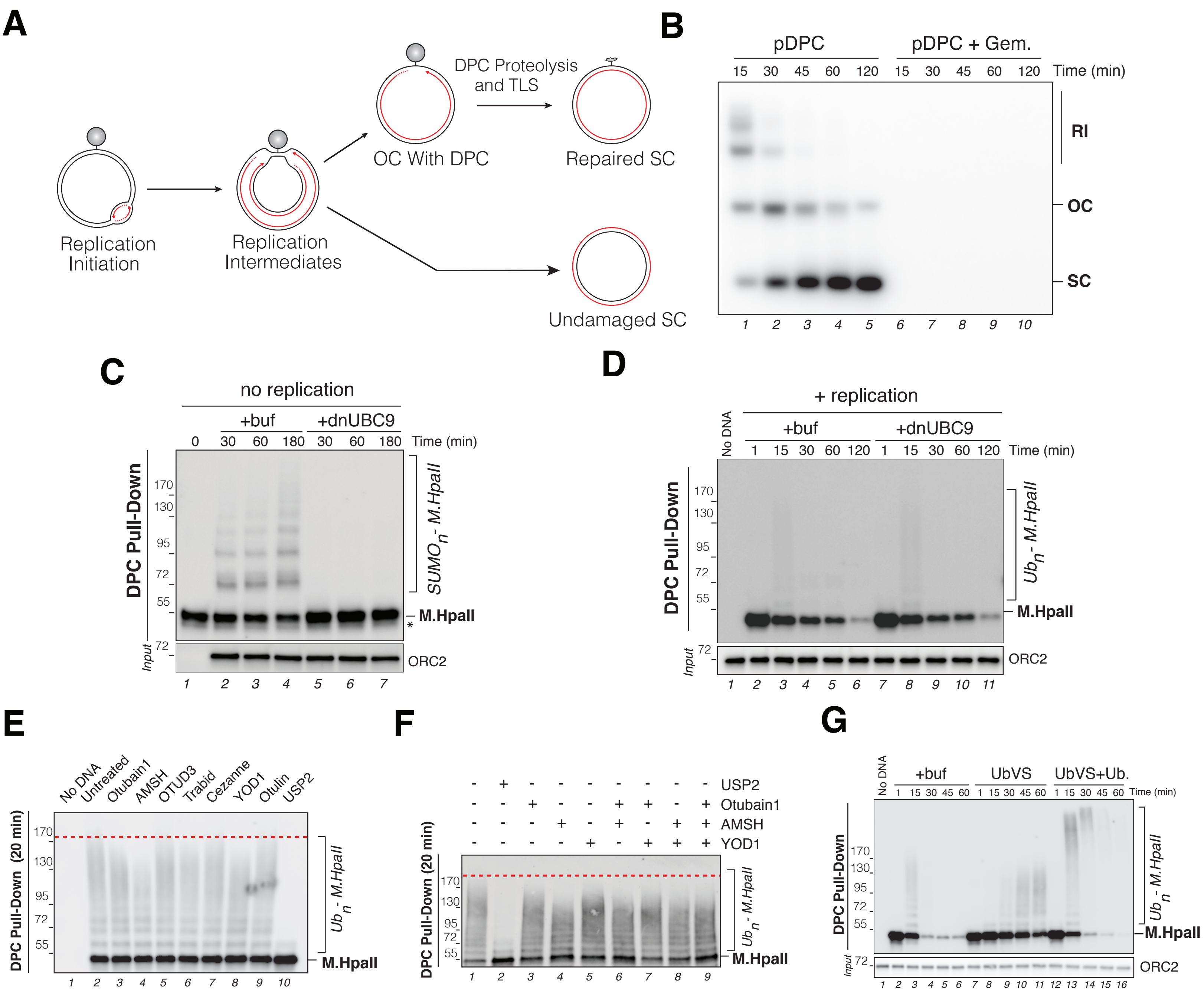
A
Replication Initiation
Replication Intermediates
OC With DPC
DPC Proteolysis and TLS
Repaired SC
Undamaged SC
B
pDPC
pDPC + Gem.
Time (min)
RI
OC
SC
C
no replication
+buf
+dnUBC9
Time (min)
DPC Pull-Down
Input
SUMOn- M.HpaII
M.HpaII
ORC2
D
+ replication
No DNA
+buf
+dnUBC9
Time (min)
DPC Pull-Down
Input
Ubn- M.HpaII
M.HpaII
ORC2
E
No DNA
Untreated
Otubain1
AMSH
OTUD3
Trabid
Cezanne
YOD1
Otulin
USP2
DPC Pull-Down (20 min)
Ubn - M.HpaII
M.HpaII
F
USP2
Otubain1
AMSH
YOD1
DPC Pull-Down (20 min)
Ubn - M.HpaII
M.HpaII
G
No DNA
+buf
UbVS
UbVS+Ub.
Time (min)
DPC Pull-Down
Input
Ubn - M.HpaII
M.HpaII
ORC2

Figure S2

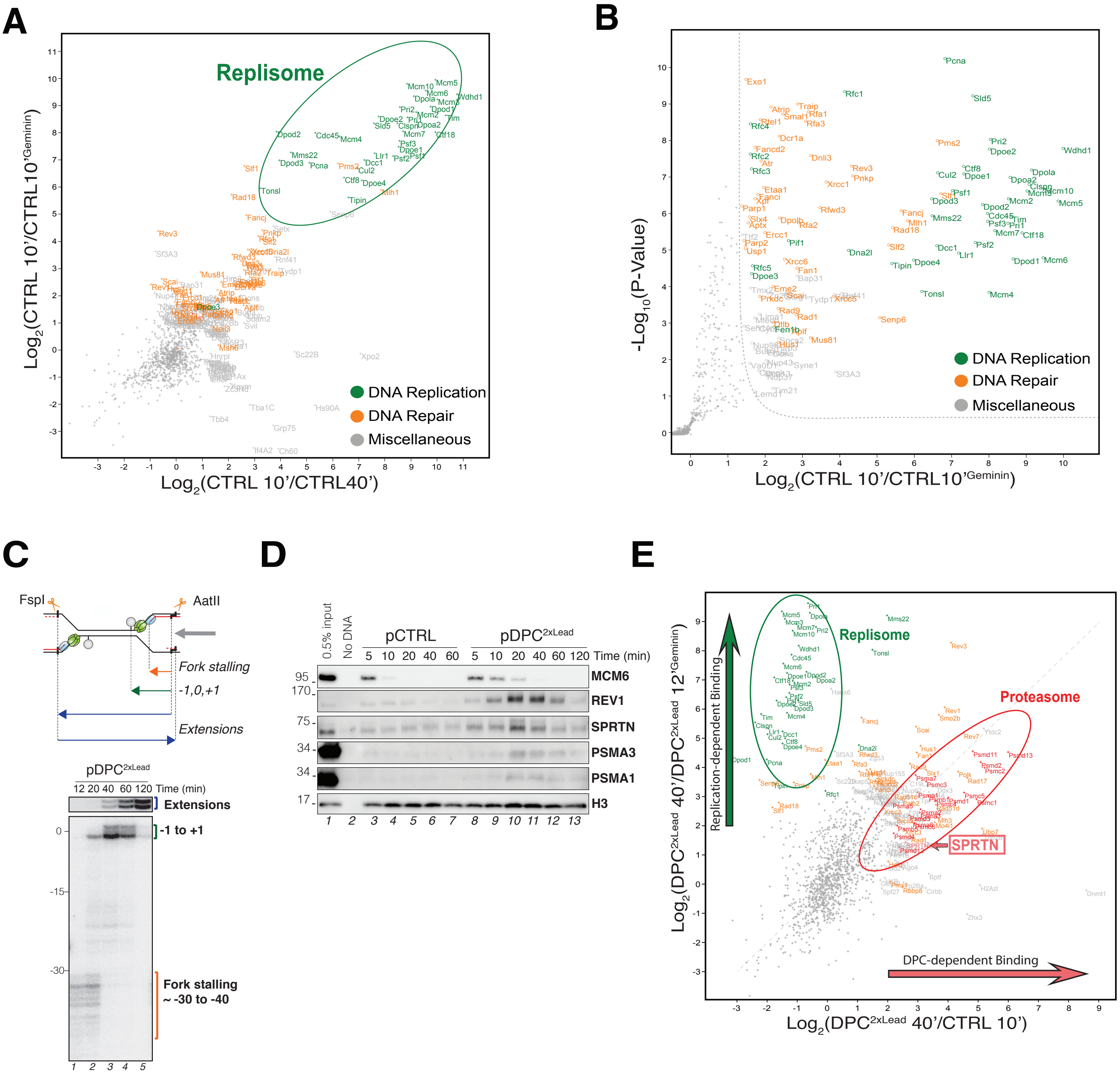
A
Replisome
Log2(CTRL 10'/CTRL10'Geminin)
Log2(CTRL 10'/CTRL40')
DNA Replication
DNA Repair
Miscellaneous
B
-Log10(P-Value)
Log2(CTRL 10'/CTRL10'Geminin)
DNA Replication
DNA Repair
Miscellaneous
C
FspI
AatII
Fork stalling
-1,0,+1
Extensions
pDPC2xLead
12 20 40 60 120 Time (min)
Extensions
-1 to +1
Fork stalling
~ -30 to -40
D
0.5% input
No DNA
pCTRL
pDPC2xLead
Time (min)
MCM6
REV1
SPRTN
PSMA3
PSMA1
H3
E
Replisome
Proteasome
Replication-dependent Binding
DPC-dependent Binding
SPRTN
Log2(DPC2xLead 40'/DPC2xLead 12'Geminin)
Log2(DPC2xLead 40'/CTRL 10')

Figure S3

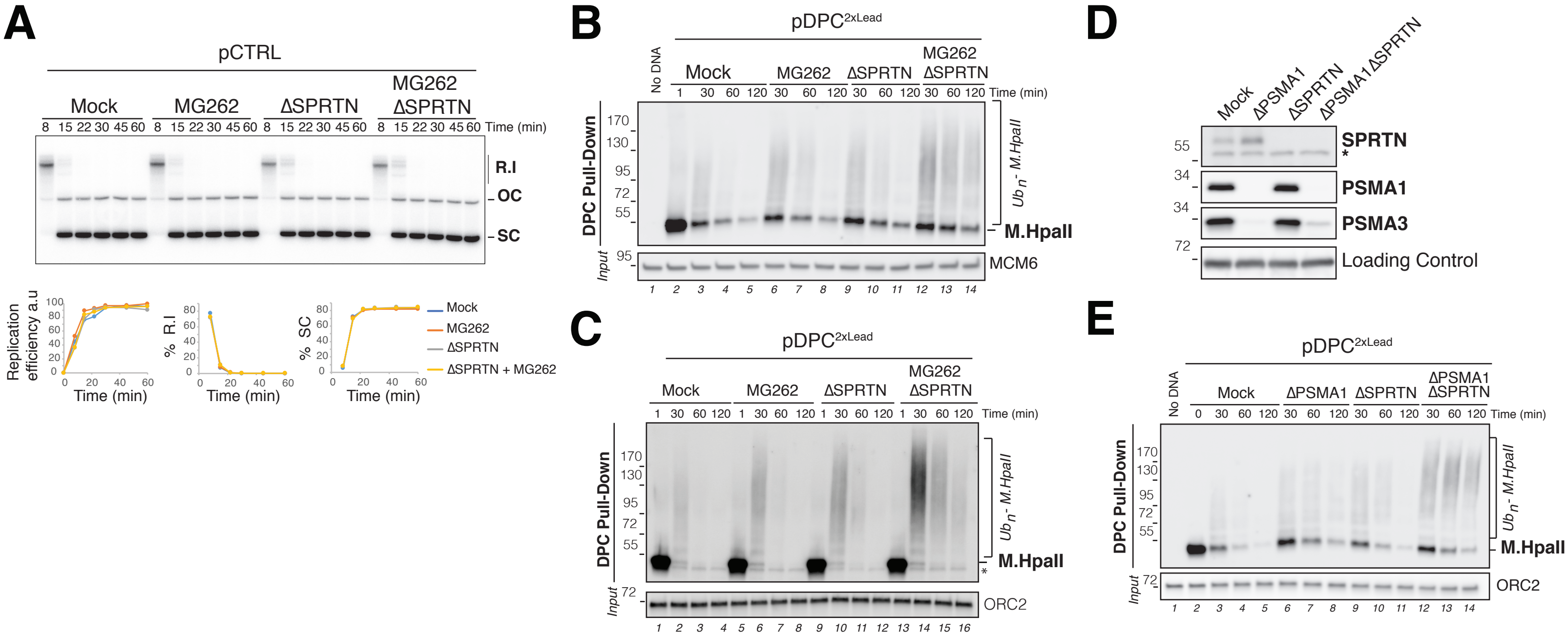
A
pCTRL
Mock
MG262
ΔSPRTN
MG262 ΔSPRTN
8 15 22 30 45 60 Time (min)
R.I
OC
SC
Replication efficiency a.u
% R.I
% SC
Time (min)
Mock
MG262
ΔSPRTN
ΔSPRTN + MG262
B
pDPC2xLead
No DNA
1 30 60 120 Time (min)
DPC Pull-Down
Input
Ubn- M.HpaII
M.HpaII
MCM6
C
pDPC2xLead
ORC2
D
Mock
ΔPSMA1
ΔSPRTN
ΔPSMA1 ΔSPRTN
SPRTN
PSMA1
PSMA3
Loading Control
E
pDPC2xLead
ΔPSMA1
ΔSPRTN
ΔPSMA1 ΔSPRTN
ORC2

Figure S4

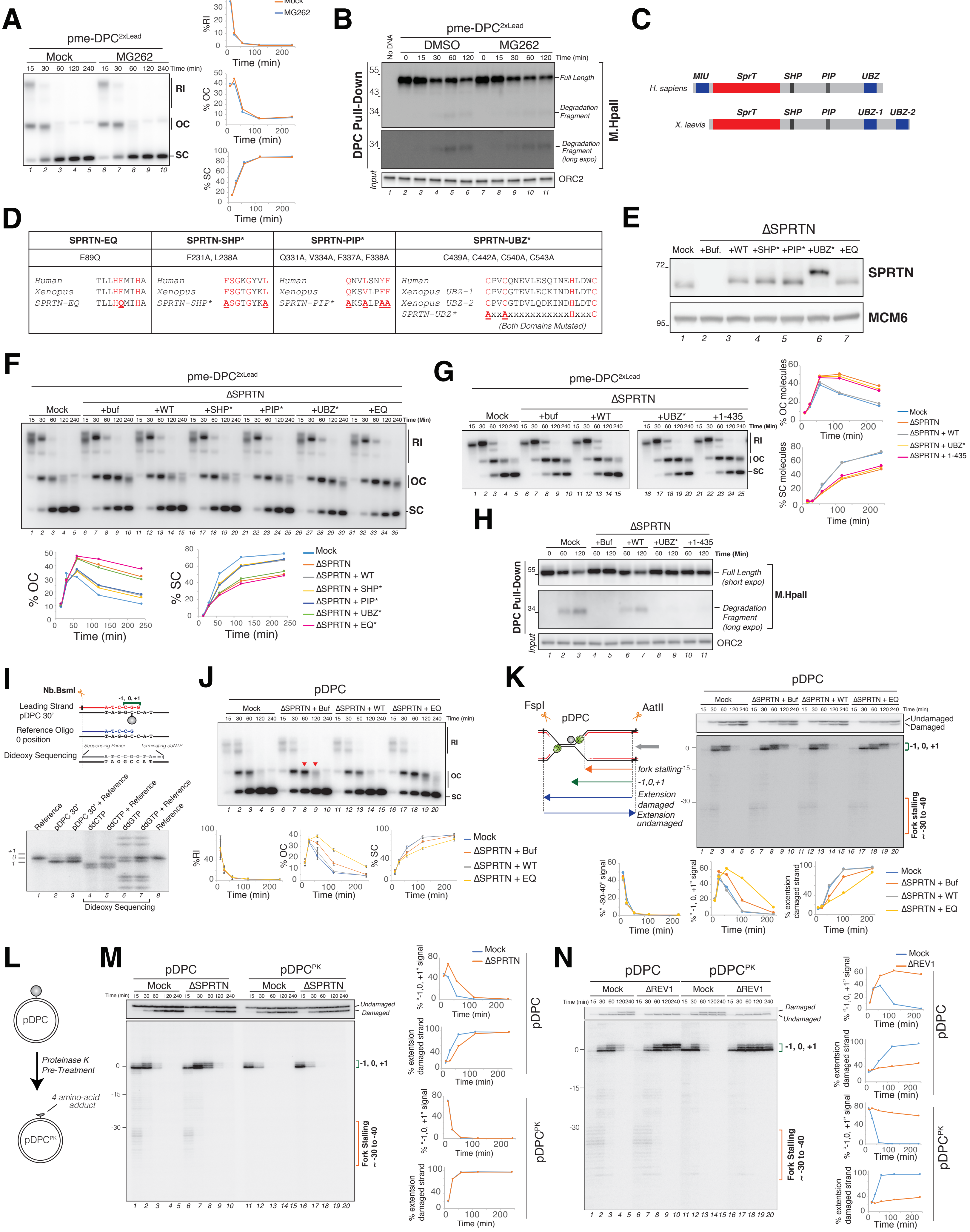
A
pme-DPC2xLead
Mock
MG262
15 30 60 120 240 15 30 60 120 240 Time (min)
RI
OC
SC
1 2 3 4 5 6 7 8 9 10
Mock
MG262
%RI
% OC
% SC
Time (min)
B
pme-DPC2xLead
No DNA
DMSO
MG262
0 15 30 60 120 0 15 30 60 120 Time (min)
55
43
34
Full Length
Degradation Fragment
Degradation Fragment (long expo)
M.HpaII
DPC Pull-Down
Input
ORC2
1 2 3 4 5 6 7 8 9 10 11
C
MIU
SprT
SHP
PIP
UBZ
H. sapiens
UBZ-1
UBZ-2
X. laevis
D
SPRTN-EQ
SPRTN-SHP*
SPRTN-PIP*
SPRTN-UBZ*
E89Q
F231A, L238A
Q331A, V334A, F337A, F338A
C439A, C442A, C540A, C543A
Human TLLHEMIHA
Xenopus TLLHEMIHA
SPRTN-EQ TLLHQMIHA
Human FSGKGYVL
Xenopus FSGTGYKL
SPRTN-SHP* ASGTGYKA
Human QNVLSNYF
Xenopus QKSVLPFF
SPRTN-PIP* AKSALPAA
Human CPVCQNEVLESQINEHLDWC
Xenopus UBZ-1 CPVCGTEVLECKINDHLDTC
Xenopus UBZ-2 CPVCGTDVLQDKINDHLDTC
SPRTN-UBZ* AxxAxxxxxxxxxxxHxxxC
(Both Domains Mutated)
E
ΔSPRTN
Mock +Buf. +WT +SHP* +PIP* +UBZ* +EQ
72
95
SPRTN
MCM6
1 2 3 4 5 6 7
F
pme-DPC2xLead
ΔSPRTN
Mock +buf +WT +SHP* +PIP* +UBZ* +EQ
Time (Min)
RI
OC
SC
% OC
% SC
Time (min)
Mock
ΔSPRTN
ΔSPRTN + WT
ΔSPRTN + SHP*
ΔSPRTN + PIP*
ΔSPRTN + UBZ*
ΔSPRTN + EQ*
G
pme-DPC2xLead
ΔSPRTN
Mock +buf +WT +UBZ* +1-435
Time (Min)
RI
OC
SC
% OC molecules
% SC molecules
Time (min)
Mock
ΔSPRTN
ΔSPRTN + WT
ΔSPRTN + UBZ*
ΔSPRTN + 1-435
H
ΔSPRTN
Mock +Buf +WT +UBZ* +1-435
0 60 120 60 120 60 120 60 120 60 120 Time (Min)
55
34
Full Length (short expo)
Degradation Fragment (long expo)
M.HpaII
DPC Pull-Down
Input
ORC2
1 2 3 4 5 6 7 8 9 10 11
I
Nb.BsmI
Leading Strand pDPC 30'
-1, 0, +1
Reference Oligo 0 position
Dideoxy Sequencing
Sequencing Primer
Terminating ddNTP
Reference
pDPC 30'
pDPC 30' + Reference
ddCTP
ddCTP + Reference
ddGTP
ddGTP + Reference
Reference
+1
0
-1
1 2 3 4 5 6 7 8
Dideoxy Sequencing
J
pDPC
Mock
ΔSPRTN + Buf
ΔSPRTN + WT
ΔSPRTN + EQ
Time (min)
RI
OC
SC
%RI
% OC
% SC
Time (min)
Mock
ΔSPRTN + Buf
ΔSPRTN + WT
ΔSPRTN + EQ
K
FspI
pDPC
AatII
fork stalling
-1,0,+1
Extension damaged
Extension undamaged
pDPC
Mock
ΔSPRTN + Buf
ΔSPRTN + WT
ΔSPRTN + EQ
Time (min)
Undamaged
Damaged
-1, 0, +1
Fork stalling ~ -30 to -40
% "-30-40" signal
% "-1, 0, +1" signal
% extension damaged strand
Time (min)
Mock
ΔSPRTN + Buf
ΔSPRTN + WT
ΔSPRTN + EQ
L
pDPC
Proteinase K Pre-Treatment
4 amino-acid adduct
pDPCPK
M
pDPC
pDPCPK
Mock
ΔSPRTN
Mock
ΔSPRTN
Time (min)
Undamaged
Damaged
-1, 0, +1
Fork Stalling ~ -30 to -40
Mock
ΔSPRTN
% "-1, 0, +1" signal
% extension damaged strand
Time (min)
pDPC
pDPCPK
N
pDPC
pDPCPK
Mock
ΔREV1
Mock
ΔREV1
Time (min)
Damaged
Undamaged
-1, 0, +1
Fork Stalling ~ -30 to -40
Mock
ΔREV1
% "-1, 0, +1" signal
% extension damaged strand
Time (min)
pDPC
pDPCPK

Figure S5

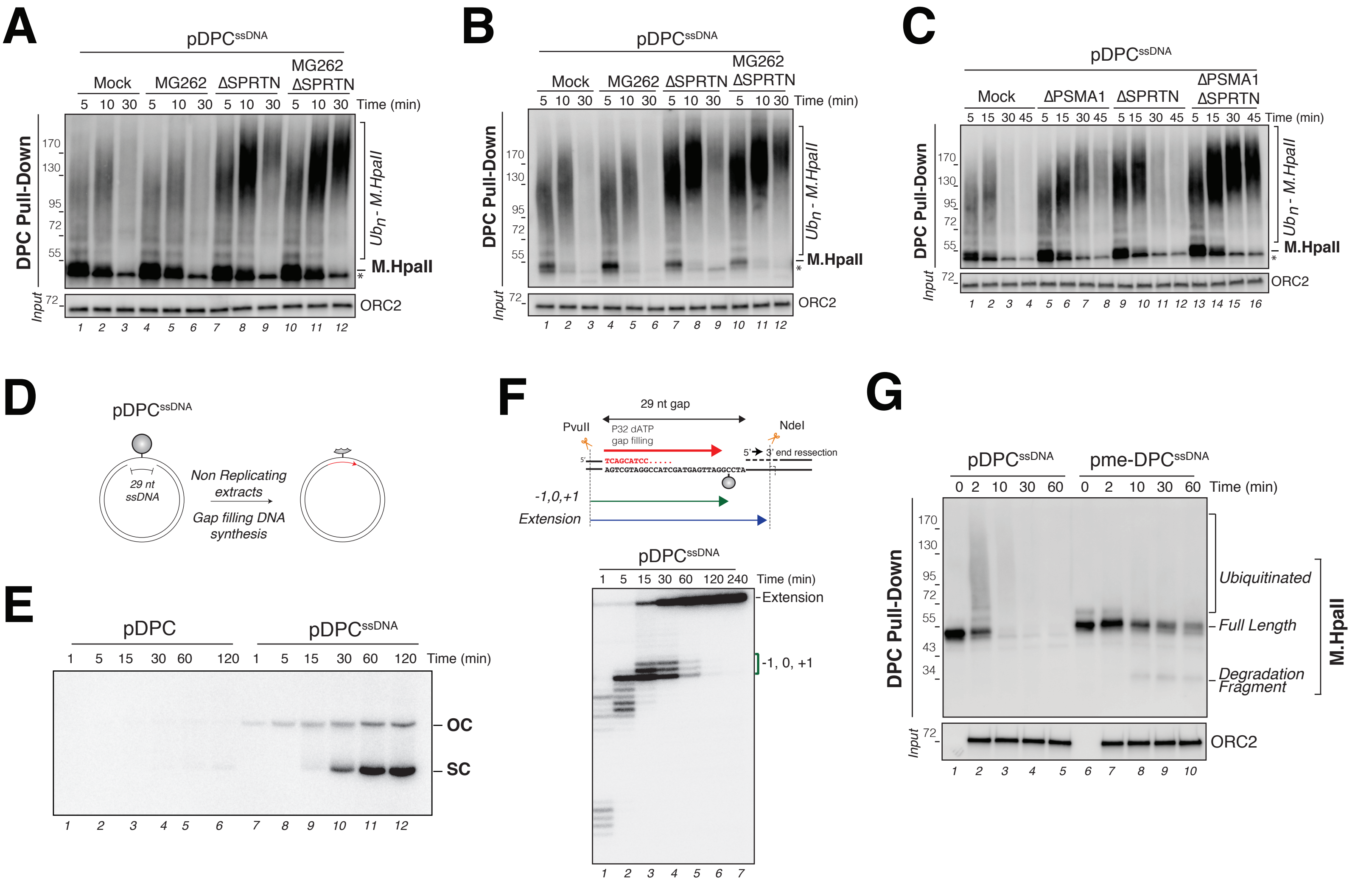
A
pDPCssDNA
Mock
MG262
ΔSPRTN
MG262 ΔSPRTN
Time (min)
DPC Pull-Down
Ubn - M.HpaII
M.HpaII
Input
ORC2
B
C
ΔPSMA1
ΔPSMA1 ΔSPRTN
D
29 nt ssDNA
Non Replicating extracts
Gap filling DNA synthesis
E
pDPC
OC
SC
F
29 nt gap
PvuII
NdeI
P32 dATP gap filling
5' 3' end ressection
TCAGCATCC.....
AGTCGTAGGCCATCGATGAGTTAGGCCTA
-1,0,+1
Extension
G
pme-DPCssDNA
Ubiquitinated
Full Length
Degradation Fragment

Figure S6

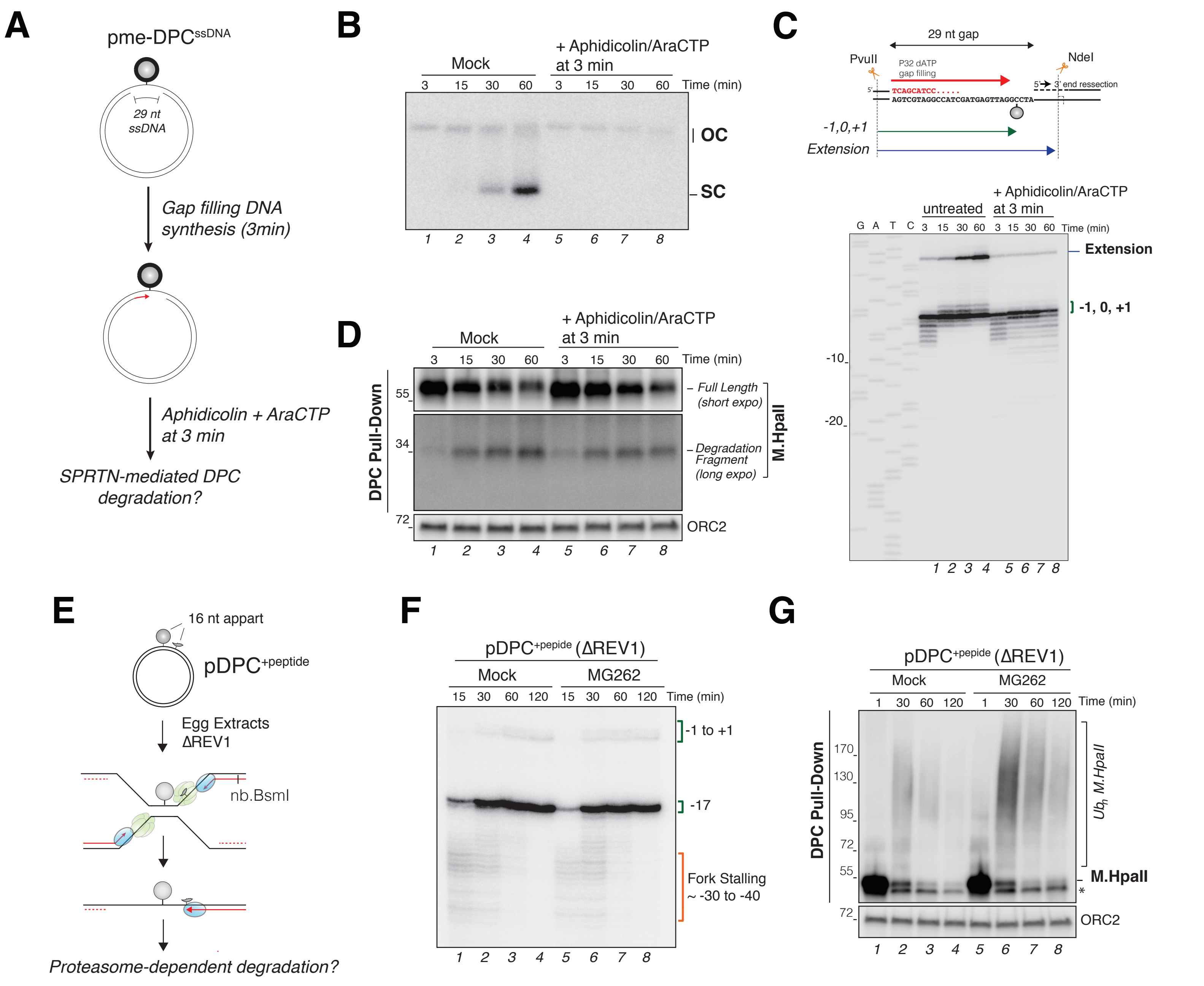

A
pme-DPC^ssDNA
29 nt ssDNA
Gap filling DNA synthesis (3min)
Aphidicolin + AraCTP at 3 min
SPRTN-mediated DPC degradation?
B
Mock
+ Aphidicolin/AraCTP at 3 min
3 15 30 60 3 15 30 60 Time (min)
OC
SC
1 2 3 4 5 6 7 8
C
29 nt gap
PvuII
P32 dATP gap filling
NdeI
5' 3' end ressection
TCAGCATCC.....
AGTCGTAGGCCATCGATGAGTTAGGCCTA
-1,0,+1
Extension
untreated
+ Aphidicolin/AraCTP at 3 min
G A T C 3 15 30 60 3 15 30 60 Time (min)
Extension
-1, 0, +1
-10
-20
1 2 3 4 5 6 7 8
D
Mock
+ Aphidicolin/AraCTP at 3 min
3 15 30 60 3 15 30 60 Time (min)
DPC Pull-Down
55
34
72
Full Length (short expo)
Degradation Fragment (long expo)
M.HpaII
ORC2
1 2 3 4 5 6 7 8
E
16 nt appart
pDPC^+peptide
Egg Extracts ΔREV1
nb.BsmI
Proteasome-dependent degradation?
F
pDPC^+pepide (ΔREV1)
Mock
MG262
15 30 60 120 15 30 60 120 Time (min)
-1 to +1
-17
Fork Stalling ~ -30 to -40
1 2 3 4 5 6 7 8
G
pDPC^+pepide (ΔREV1)
Mock
MG262
1 30 60 120 1 30 60 120 Time (min)
DPC Pull-Down
170
130
95
72
55
Ub_n M.HpaII
M.HpaII
*
72
ORC2
1 2 3 4 5 6 7 8

Figure S7

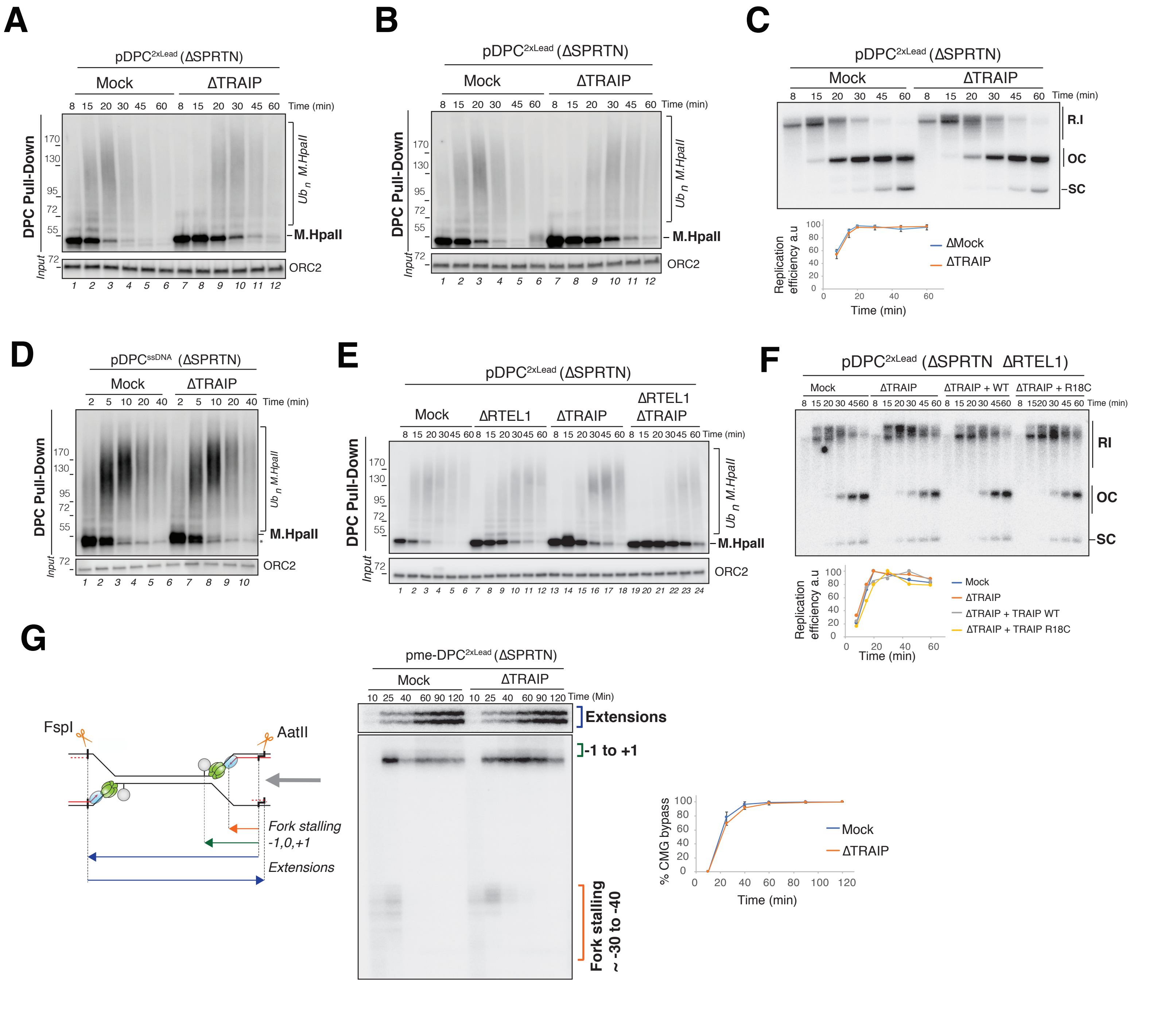
A
pDPC2xLead (ΔSPRTN)
Mock
ΔTRAIP
8 15 20 30 45 60 8 15 20 30 45 60 Time (min)
DPC Pull-Down
Ub n M.HpaII
M.HpaII
Input
ORC2
1 2 3 4 5 6 7 8 9 10 11 12
B
pDPC2xLead (ΔSPRTN)
Mock
ΔTRAIP
8 15 20 30 45 60 8 15 20 30 45 60 Time (min)
DPC Pull-Down
Ub n M.HpaII
M.HpaII
Input
ORC2
1 2 3 4 5 6 7 8 9 10 11 12
C
pDPC2xLead (ΔSPRTN)
Mock
ΔTRAIP
8 15 20 30 45 60 8 15 20 30 45 60 Time (min)
R.I
OC
SC
Replication efficiency a.u
Time (min)
ΔMock
ΔTRAIP
D
pDPCssDNA (ΔSPRTN)
Mock
ΔTRAIP
2 5 10 20 40 2 5 10 20 40 Time (min)
DPC Pull-Down
Ub n M.HpaII
M.HpaII
Input
ORC2
1 2 3 4 5 6 7 8 9 10
E
pDPC2xLead (ΔSPRTN)
Mock
ΔRTEL1
ΔTRAIP
ΔRTEL1 ΔTRAIP
Time (min)
DPC Pull-Down
Ub n M.HpaII
M.HpaII
Input
ORC2
1 2 3 4 5 6 7 8 9 10 11 12 13 14 15 16 17 18 19 20 21 22 23 24
F
pDPC2xLead (ΔSPRTN ΔRTEL1)
Mock
ΔTRAIP
ΔTRAIP + WT
ΔTRAIP + R18C
Time (min)
RI
OC
SC
Replication efficiency a.u
Time (min)
Mock
ΔTRAIP
ΔTRAIP + TRAIP WT
ΔTRAIP + TRAIP R18C
G
FspI
AatII
Fork stalling -1,0,+1
Extensions
pme-DPC2xLead (ΔSPRTN)
Mock
ΔTRAIP
10 25 40 60 90 120 10 25 40 60 90 120 Time (Min)
Extensions
-1 to +1
Fork stalling ~ -30 to -40
% CMG bypass
Time (min)
Mock
ΔTRAIP
