# Supplementary material for: Replication-Coupled DNA-Protein Crosslink Repair by SPRTN and the Proteasome in Xenopus Egg Extracts
Source: Mol Cell. 2019 Feb 7;73(3):574–588.e7. doi: 10.1016/j.molcel.2018.11.024 (PMC6375733; doi:10.1016/j.molcel.2018.11.024)
Supplement: Document S2. Article plus Supplemental Information [file mmc4.pdf]

Molecular Cell

Article

Replication-Coupled DNA-Protein Crosslink Repair by SPRTN and the Proteasome in *Xenopus* Egg Extracts

Graphical Abstract

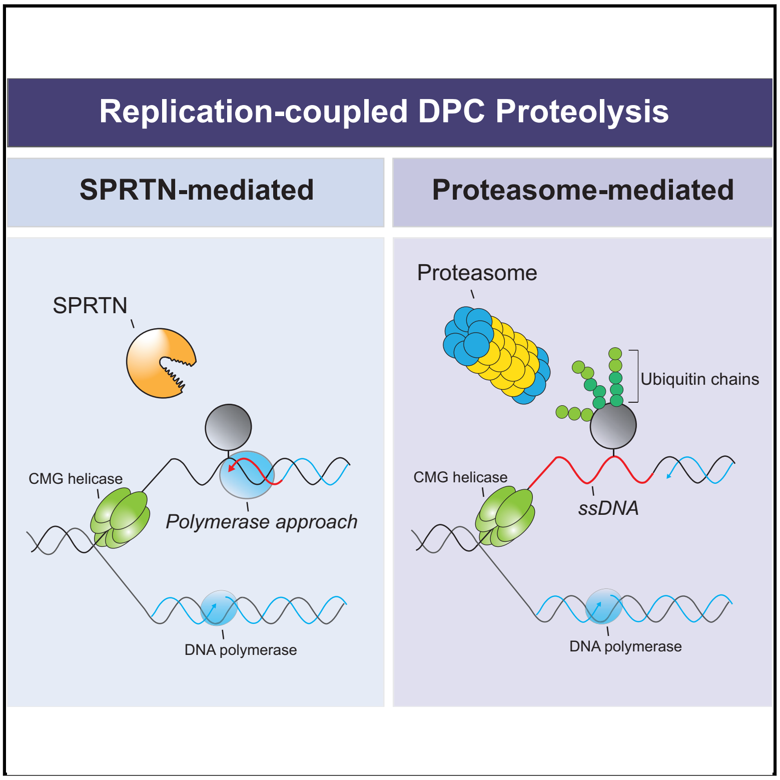

Authors

Nicolai B. Larsen, Alan O. Gao, Justin L. Sparks, ..., Markus Räschle, Johannes C. Walter, Julien P. Duxin

Correspondence

johannes_walter@hms.harvard.edu (J.C.W.), julien.duxin@cpr.ku.dk (J.P.D.)

In Brief

DNA-protein crosslinks (DPCs) pose major threats to genomic integrity. Using a cell-free system, Larsen et al. show that SPRTN and the proteasome operate as independent replication-coupled DPC proteases. The existence of two DPC proteases with distinct modes of action facilitates the degradation of chemically diverse DPCs.

Highlights

- The proteasome and SPRTN can each degrade DPCs during DNA replication
- Efficient DPC ubiquitylation and proteasome targeting requires TRAIP
- SPRTN can degrade DPCs that are not ubiquitylated
- SPRTN-mediated DPC degradation is a post-replicative process

Larsen et al., 2019, Molecular Cell *73*, 574–588
February 7, 2019 © 2018 The Authors. Published by Elsevier Inc.
https://doi.org/10.1016/j.molcel.2018.11.024

Molecular Cell
Article

Replication-Coupled DNA-Protein Crosslink Repair by SPRTN and the Proteasome in *Xenopus* Egg Extracts

Nicolai B. Larsen,[1,6] Alan O. Gao,[1,2,6] Justin L. Sparks,[2,6] Irene Gallina,[1] R. Alex Wu,[2] Matthias Mann,[1,3] Markus Räschle,[4] Johannes C. Walter,[2,5,*] and Julien P. Duxin[1,7,*]
[1]Faculty of Health and Medical Sciences, Novo Nordisk Foundation Center for Protein Research, University of Copenhagen, DK-2200 Copenhagen, Denmark
[2]Department of Biological Chemistry and Molecular Pharmacology, Harvard Medical School, Boston, MA 02115, USA
[3]Department of Proteomics and Signal Transduction, Max Planck Institute of Biochemistry, 82152 Martinsried, Germany
[4]Department of Molecular Biotechnology and Systems Biology, Technical University of Kaiserslautern, 67653 Kaiserslautern, Germany
[5]Howard Hughes Medical Institute, Department of Biological Chemistry and Molecular Pharmacology, Harvard Medical School, Boston, MA 02115, USA
[6]These authors contributed equally
[7]Lead Contact
*Correspondence: johannes_walter@hms.harvard.edu (J.C.W.), julien.duxin@cpr.ku.dk (J.P.D.)
https://doi.org/10.1016/j.molcel.2018.11.024

SUMMARY

DNA-protein crosslinks (DPCs) are bulky lesions that interfere with DNA metabolism and therefore threaten genomic integrity. Recent studies implicate the metalloprotease SPRTN in S phase removal of DPCs, but how SPRTN is targeted to DPCs during DNA replication is unknown. Using *Xenopus* egg extracts that recapitulate replication-coupled DPC proteolysis, we show that DPCs can be degraded by SPRTN or the proteasome, which act as independent DPC proteases. Proteasome recruitment requires DPC polyubiquitylation, which is partially dependent on the ubiquitin ligase activity of TRAIP. In contrast, SPRTN-mediated DPC degradation does not require DPC polyubiquitylation but instead depends on nascent strand extension to within a few nucleotides of the lesion, implying that polymerase stalling at the DPC activates SPRTN on both leading and lagging strand templates. Our results demonstrate that SPRTN and proteasome activities are coupled to DNA replication by distinct mechanisms that promote replication across immovable protein barriers.

INTRODUCTION

Vertebrate chromatin is composed of myriad proteins that perform a multitude of functions. Sometimes, these proteins are covalently trapped on DNA, yielding DNA-protein crosslinks (DPCs) (Barker et al., 2005; Ide et al., 2011; Tretyakova et al., 2015). While DPCs generated by most crosslinking agents (e.g., formaldehyde, cisplatin-based chemotherapeutics) link proteins to uninterrupted duplex DNA (type I DPCs), abortive reactions by DNA repair enzymes such as topoisomerase I and II form DPCs that are flanked on one side by a single-stranded (type II DPCs) or double-stranded DNA break (type III DPCs), respectively (Ide et al., 2011). Left unrepaired, DPCs stall or inhibit DNA replication and transcription and thereby threaten genomic integrity (Chválová et al., 2007; Duxin et al., 2014; Kuo et al., 2007; Nakano et al., 2012; Novakova et al., 2003).

Given the frequency and cytotoxicity of DPC lesions, cells have evolved pathways to promote their removal. While nucleotide excision repair and homologous recombination have been linked to DPC repair (Ide et al., 2011), recent experiments in yeast identified the metalloprotease Wss1 as a dedicated DPC-repair factor (Stingele et al., 2014). Wss1 removes DPCs from the genome by degrading crosslinked proteins (Balakirev et al., 2015; Stingele et al., 2014). In contemporaneous experiments, we recapitulated replication-coupled DPC proteolysis in *Xenopus* egg extracts (Duxin et al., 2014). In this mechanism, a type I DPC encountered by the replisome is degraded to a short peptide adduct. Degradation of the DPC facilitates replisome bypass and DNA synthesis across the lesion by the translesion synthesis (TLS) polymerase complex Rev1-Polζ (Duxin et al., 2014). In this manner, the replisome simultaneously overcomes DPCs and clears them from the genome. Collectively, the experiments in yeast and in *Xenopus* established the existence of a dedicated, S-phase proteolytic DPC-repair pathway, although the protease acting in vertebrates remained elusive at the time.

Studies in mammalian cells suggest that the proteasome also participates in DPC removal (Baker et al., 2007; Desai et al., 1997; Lin et al., 2008; Mao et al., 2001; Quiñones et al., 2015; Zecevic et al., 2010). Proteasome inhibition prevents the removal of different types of DPCs, including trapped topoisomerases and DNA Polβ (Desai et al., 1997; Lin et al., 2008; Mao et al., 2001; Quiñones et al., 2015), and sensitizes cells to formaldehyde treatment (Ortega-Atienza et al., 2015). In addition, DPC polyubiquitylation was reported in the case of covalent topoisomerase I (Desai et al., 1997). However, polyubiquitylation of the more abundant type I DPCs could not be observed (Nakano et al., 2009), and it is therefore unclear whether DPCs are generally targeted by the proteasome. In *Xenopus* egg extracts, inhibition of the

 © 2018 The Authors. Published by Elsevier Inc.
This is an open access article under the CC BY license (http://creativecommons.org/licenses/by/4.0/).

proteasome on its own does not significantly stabilize type I DPCs during DNA replication (Duxin et al., 2014). Therefore, whether the proteasome acts on different types of DPCs and whether this process operates during DNA replication remain open questions.

Recently, the metalloprotease SPARTAN (SPRTN) has been implicated in DPC degradation in higher eukaryotes. SPRTN shares homology with the yeast DPC protease Wss1 and is proposed to be functionally similar (Stingele et al., 2015; Vaz et al., 2017). In humans, mutations in SPRTN that compromise its protease activity cause Ruijs-Aalfs syndrome (RJALS), which is characterized by genomic instability, premature aging, and hepatocellular carcinoma (Lessel et al., 2014). In mice, loss of SPRTN is embryonically lethal, and conditional inactivation of SPRTN in murine embryonic fibroblasts (MEFs) blocks cell proliferation (Maskey et al., 2014). Although SPRTN was initially characterized as a regulator of TLS (Centore et al., 2012; Davis et al., 2012; Mosbech et al., 2012), several recent reports suggest that its essential role in genome maintenance involves DPC proteolysis (Lopez-Mosqueda et al., 2016; Maskey et al., 2017; Mórocz et al., 2017; Stingele et al., 2016; Vaz et al., 2016). SPRTN is predominantly expressed in S phase and associates with replisome components (Ghosal et al., 2012; Kim et al., 2013; Mosbech et al., 2012; Vaz et al., 2016). In the absence of SPRTN, cells accumulate DPCs and exhibit impaired replication fork progression (Lessel et al., 2014; Mórocz et al., 2017; Vaz et al., 2016). The data suggest that DPCs readily form *in vivo* and that cells rely on SPRTN-dependent DPC removal to suppress genome instability, cancer, and aging.

SPRTN proteolytic activity is regulated via different mechanisms. First, SPRTN undergoes monoubiquitylation (Mosbech et al., 2012), which prevents its recruitment to chromatin (Stingele et al., 2016). DPC induction triggers SPRTN deubiquitylation by an unknown ubiquitin protease, allowing SPRTN to localize to chromatin and initiate DPC degradation (Stingele et al., 2016). Once SPRTN is recruited to chromatin, DNA binding stimulates its protease activity (Lopez-Mosqueda et al., 2016; Mórocz et al., 2017; Stingele et al., 2016; Vaz et al., 2016), and evidence indicates that SPRTN is uniquely activated by single-stranded DNA (ssDNA) (Stingele et al., 2016). SPRTN also degrades itself, which may switch off its proteolytic function when repair is complete (Stingele et al., 2016; Vaz et al., 2016). Although these findings suggest that SPRTN activity is subject to elaborate regulation, they do not explain how SPRTN is specifically directed to DPCs during DNA replication or how non-specific replisome destruction is avoided.

We investigated the molecular mechanisms that link DPC degradation to DNA replication. Here, we report that SPRTN and the proteasome function as two replication-coupled DPC proteases. Proteasome recruitment to DPCs depends on replication-dependent DPC ubiquitylation. In contrast, SPRTN-mediated DPC degradation can occur in the absence of DPC ubiquitylation, but instead requires the extension of a nascent strand to the DPC. Our results reveal how SPRTN and proteasome activities are targeted to DPCs to facilitate replication across these covalent protein barriers.

RESULTS

DPCs Are Ubiquitylated and Degraded during DNA Replication

To investigate DPC repair, the 45-kDa DNA methyltransferase HpaII (M.HpaII) was trapped at a fluorinated sequence on a plasmid to generate a type I DPC (Chen et al., 1991). During replication of the resulting plasmid (pDPC) in *Xenopus* egg extracts, converging forks transiently stall at the DPC, after which daughter plasmid molecules are resolved (Figure S1A; Duxin et al., 2014). The daughter molecule containing the DPC initially migrates as an open circular (OC) species and is then gradually converted to a supercoiled (SC) repair product through proteolysis of the DPC and TLS across the resulting peptide adduct (Figures S1A and S1B). To monitor the integrity of the DPC, we pulled down the plasmid under stringent conditions, digested the DNA, and analyzed M.HpaII via immunoblotting (Figure 1A). At 15 min, when replication was under way (Figure S1B), covalently attached M.HpaII migrated as a ladder of slow mobility species that subsequently disappeared (Figure 1B, lanes 2–4). The addition of FLAG-ubiquitin to the extract shifted the mobility of the M.HpaII species (Figure 1C, lane 4), indicating that they correspond to ubiquitylated M.HpaII. These slow mobility M.HpaII species were also precipitated by FLAG resin during DNA replication (Figure 1D, lane 6). When DNA replication initiation was blocked with Geminin (Figure S1B) (Tada et al., 2001; Wohlschlegel et al., 2000), M.HpaII persisted in a largely unmodified form (Figure 1B, lanes 5–6), demonstrating that DPC ubiquitylation and degradation are dependent on DNA replication. In the absence of replication, a different set of modified M.HpaII species slowly appeared (Figure 1B, lane 6). These species were cleaved by the SUMO protease Ulp1 (data not shown), and their appearance was dependent on the activity of the SUMO ligase UBC9 (Figure S1C). In contrast, the replication-dependent species did not involve SUMOylation (Figure S1D). Therefore, DPCs undergo both replication-dependent ubiquitylation, which contributes to proteolysis (see below), and replication-independent SUMOylation, the function of which is still unknown.

Replication forks promote the destruction of DPCs encountered on the leading and lagging strand templates (Duxin et al., 2014). However, because the replicative Cdc45-Mcm-GINS (CMG) helicase translocates on the leading strand template (Fu et al., 2011), it is possible that DPCs encountered on the two parental strands undergo different processing. To address this, we replicated a plasmid containing a lac repressor array that is flanked on one side by a DPC on the top or bottom strand (Figure 1E). The rightward fork stalls at the array, whereas the leftward fork encounters the DPC on the leading or lagging strand templates, respectively (Dewar et al., 2015; Duxin et al., 2014). As shown in Figure 1E, DPCs encountered on either strand were ubiquitylated and degraded, suggesting that leading and lagging strand DPCs are recognized and processed similarly.

We next asked whether a specific ubiquitin linkage is formed on the DPC. The obutain1 and associated molecule with the SH3 domain of the signal transducing adaptor molecule (AMSH) deubiquitinases (DUBs), which are specific for Lys-48 and Lys-63 linkages, respectively, partially reduced the length

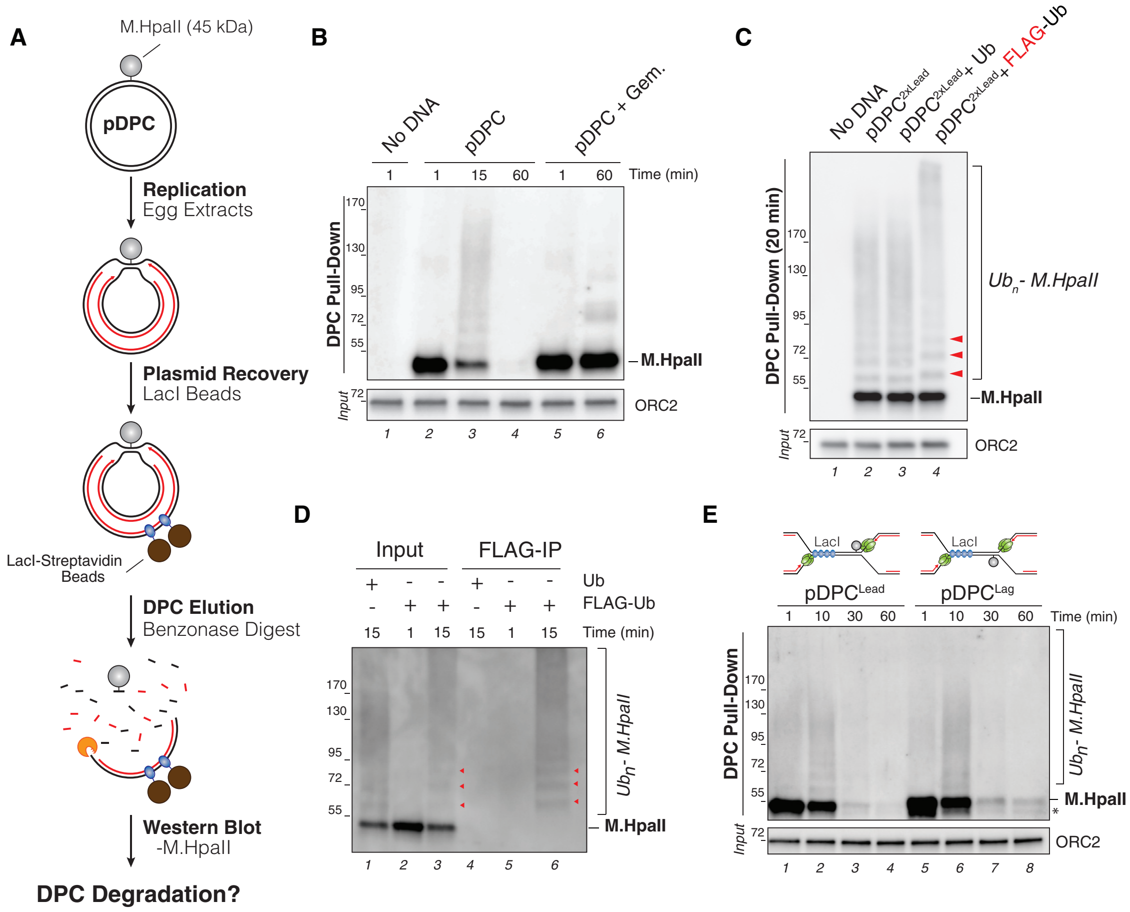

Figure 1. Replication-Coupled Ubiquitylation and Degradation of a DPC

(A) Schematic of the DPC recovery assay.

(B) pDPC was replicated in egg extracts. Geminin (+ Gem.) was added where indicated to block DNA replication. DPCs were recovered as illustrated in (A) at the indicated time points, and DPCs were blotted with a M.HpaII antibody. Input samples were blotted with an origin recognition complex subunit 2 (ORC2) antibody.

(C) pDPC^{2xLead}, a plasmid containing two DPCs (one on each leading strand; see Figure 2A), was replicated in egg extracts supplemented with free ubiquitin (Ub) or FLAG-ubiquitin. At 20 min, DPCs were recovered and blotted as in (B). Red arrowheads indicate the mobility shift induced by FLAG-ubiquitin.

(D) pDPC^{2xLead} was replicated in egg extracts supplemented with free ubiquitin (Ub) or FLAG-ubiquitin. At the indicated time point, DPCs were recovered as in (B) (Input) and immunoprecipitated with anti-FLAG-resin (FLAG-IP). Ubiquitylated DPCs were detected with M.HpaII antibody. Red arrowheads indicate the location of mono-, di-, and tri-ubiquitylated M.HpaII.

(E) pDPC^{Lead} or pDPC^{Lag} was replicated in egg extract in the presence of LacI to ensure that a single replication fork encounters the DPC (Duxin et al., 2014). Recovered DPCs were blotted as in (B). The asterisk indicates residual uncrosslinked M.HpaII.

of M.HpaII-linked ubiquitin chains (Figure S1E). YOD1, which hydrolyzes all of the other ubiquitin linkages, also partially cleaved DPC ubiquitin chains (Figure S1E). Polyubiquitylated M.HpaII persisted even after treatment with a DUB cocktail that targeted all linkages (Figure S1F), but treatment with ubiquitin carboxyl-terminal hydrolase 2 (USP2), which cleaves ubiquitin moieties, attached directly to target proteins (Hospenthal et al., 2015), collapsed M.HpaII into a single band (Figures S1E and S1F). These results suggest that ubiquitylated M.HpaII contains multiple ubiquitin chain types added by one or more ubiquitin ligases.

Previously, we demonstrated that DPC degradation is drastically inhibited by ubiquitin-vinyl-sulfone (UbVS), which inhibits DUBs and thereby depletes free ubiquitin in extracts (Dimova et al., 2012; Duxin et al., 2014). Consistent with this result, UbVS delayed the ubiquitylation of M.HpaII and strongly inhibited DPC proteolysis (Figure S1G, lanes 7–11), effects that were rescued with free ubiquitin (Figure S1G, lanes 12–16). We conclude that when a replication fork encounters a DPC, the DPC undergoes extensive polyubiquitylation before being degraded.

SPRTN and the Proteasome Accumulate on Replicating DPC Plasmids

To identify DPC protease(s), we combined plasmid pull-down with quantitative high-resolution mass spectrometry (PP-MS) (Figure 2A). In contrast to chromatin MS (CHROMASS), which detects proteins on randomly damaged sperm chromatin (Räschle et al., 2015), PP-MS identifies proteins associated with defined DNA lesions and discrete repair intermediates (Figure 2A). To

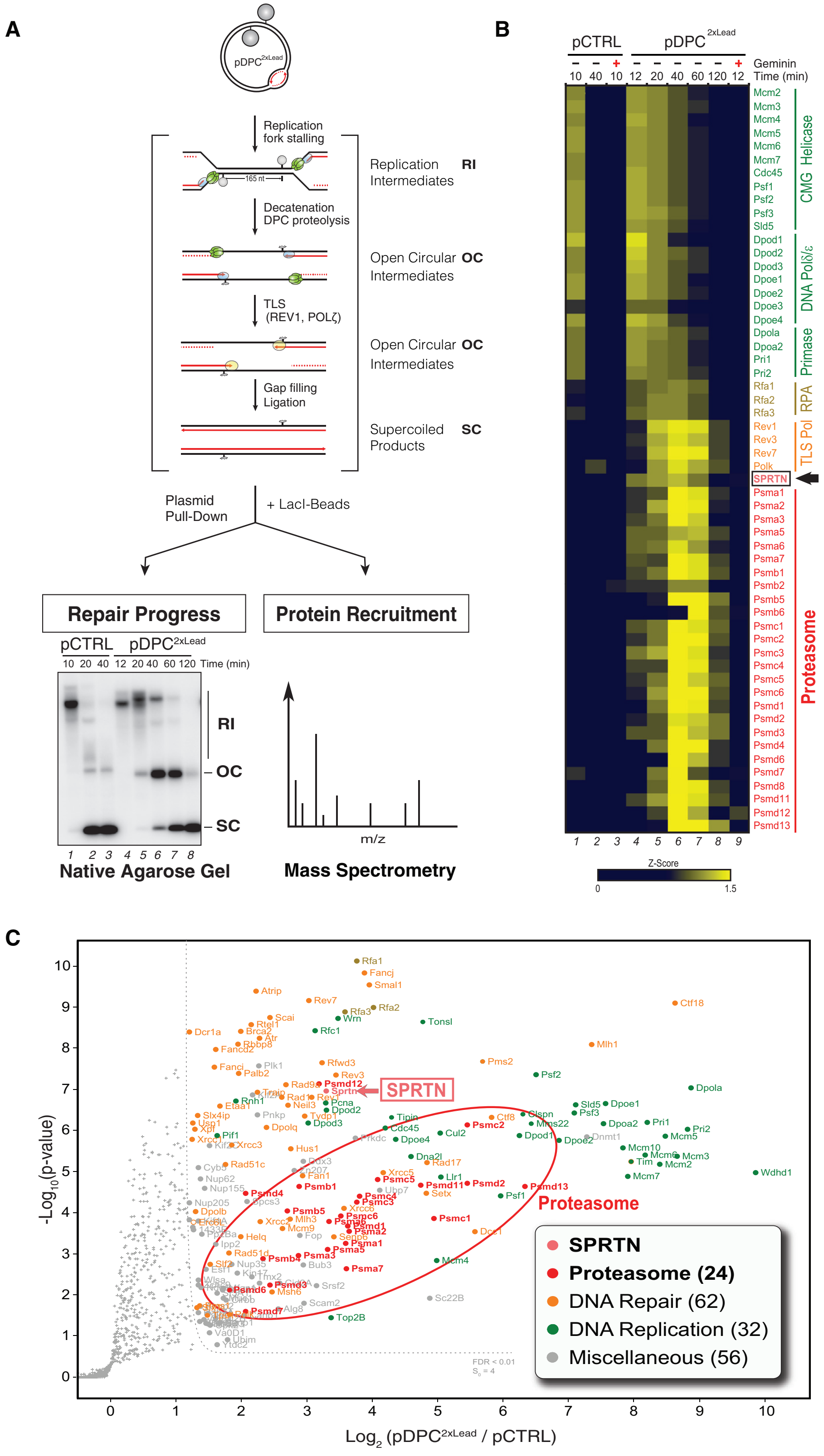

(legend on next page)

validate PP-MS, we first replicated an undamaged plasmid (pCTRL) in egg extracts and isolated it during (10 min) or after (40 min) replication (Figure 2A, lanes 1–3). As expected, CMG, all three replicative DNA polymerases, and most of the replisome components, were significantly enriched when replication was ongoing (Figures 2B, columns 1–2, and S2A), whereas the addition of Geminin abolished their recruitment (Figures 2B, column 3, S2A, and S2B). Thus, PP-MS is a robust method to detect the proteins associated with plasmids in egg extracts.

We next applied PP-MS to DPC repair. To maximize the yield of DPC repair factors, we replicated pDPC^{2xLead}, a plasmid containing two DPCs positioned 165 nt apart, such that both converging forks encountered a DPC on the leading strand template (Figure 2A). Following fork stalling at the DPC, the daughter molecules underwent decatenation, and the OC plasmids were repaired by TLS (Figures 2A, lanes 4–8, and S2C). Consistent with replication fork stalling at leading strand DPCs (Figure S2C) (Duxin et al., 2014; Fu et al., 2011), replisome components persisted for up to 40 min on pDPC^{2xLead} (Figures 2B, columns 4–8, and S2D, MCM6 panel). The TLS polymerases REV1, Polζ, and Polκ were recruited to pDPC^{2xLead} following replisome unloading (Figures 2B and S2D, REV1 panel), and their peak binding correlated with the transition from OC to SC plasmids (Figure 2A, lanes 6–7), which depends on the REV1-Polζ complex (Duxin et al., 2014). By 120 min, when all of the molecules had undergone replication-coupled repair (Figures 2A, lane 8, and 2C), repair factors were largely lost from DNA (Figure 2B, column 8).

Consistent with recent findings that SPRTN functions in S phase DPC repair, we observed a specific enrichment of SPRTN on replicating pDPC^{2xLead} (Figure 2B, columns 4–8). SPRTN recruitment occurred during the peak of proteolysis (20–60 min), depended on DNA replication, and was not observed on pCTRL (Figures 2B, 2C, S2D, and S2E). The 26S proteasome was also specifically recovered on DPC plasmids. A total of 26 proteasome subunits showed significant enrichment on pDPC^{2xLead} compared to pCTRL (Figure 2B), and their recruitment also peaked between 20 and 60 min and depended on DNA replication (Figures 2B, 2C, S2D, and S2E). Collectively, these experiments provide an unbiased resource of candidate DPC repair factors (Tables S1 ans S2) and single out SPRTN and the proteasome as two proteases that may mediate DPC destruction in egg extracts. They also illustrate the ability of PP-MS to identify proteins associated with different stages in the repair of a chemically defined DNA lesion.

Both SPRTN and the Proteasome Degrade DPCs during Replication

To explore the roles of SPRTN and the proteasome in DPC proteolysis, we replicated pDPC^{2xLead} in the presence of the proteasome inhibitor MG262 or in extracts depleted of SPRTN (Figure 3A). Proteasome inhibition did not significantly inhibit DPC repair and only caused a minimal delay in the conversion of OC intermediates to SC repair products (Figure 3B, lanes 6–10) (Duxin et al., 2014). SPRTN-depleted extracts exhibited a more pronounced but still transient persistence of OC molecules compared to mock-depleted extracts (Figure 3B, lanes 11–15). In combination, however, SPRTN depletion and MG262 treatment strongly delayed the conversion of OC intermediates to SC products (Figures 3B, lanes 16–20) without affecting DNA replication kinetics (Figure S3A), indicating that in the absence of both proteases, DPC repair is specifically inhibited. Accordingly, whereas SPRTN depletion or MG262 treatment alone resulted in a modest delay in DPC degradation (Figures 3C, lanes 6–13, S3B, and S3C for independent experiments), in combination these treatments stabilized ubiquitylated M.HpaII species for up to 2 hr (Figures 3C, lanes 14–17, S3B, and S3C). DPC degradation and generation of SC repair products were largely restored by the addition of recombinant wild-type (WT) SPRTN but not catalytically inactive (EQ) SPRTN (Figures 3D–3F). We also confirmed the role of the proteasome on DPC degradation via immunodepletion, which closely resembled MG262 treatment (Figures S3D and S3E).

Given that depletion of SPRTN or inhibition of the proteasome did not prevent DPC degradation, we hypothesized that these two proteases act independently. To test this idea, we pulled down pDPC^{2xLead} from extracts depleted of either SPRTN or the proteasome. As shown in Figure 3G, neither SPRTN nor proteasome depletion impaired the recruitment of the other protease to chromatin. We conclude that during DNA replication, both SPRTN and the proteasome can degrade DPCs independently of each other.

SPRTN Can Degrade Non-ubiquitylated DPCs

We next investigated the role of DPC ubiquitylation. To this end, we chemically methylated the lysines of M.HpaII (Walter et al., 2006), thereby generating a DPC that cannot be ubiquitylated (me-DPC) (Figure 4A). As shown in Figure 4B, methylated M.HpaII recovered from replication reactions migrated as a single, unmodified band, reflecting a block of DPC ubiquitylation (lanes 8–13). Notably, even in the absence of ubiquitylation, plasmid-associated M.HpaII slowly decreased, and an M.HpaII degradation product of ~34 kDa accumulated (Figure 4B, lanes 10–13; note that the M.HpaII antibody is detecting one of possibly multiple degradation products). In plasmid pull-downs, M.HpaII methylation abolished proteasome recruitment, while SPRTN recruitment was reduced but still detectable (Figure 4C, lanes 9–14), suggesting that SPRTN is the sole protease acting on the methylated DPC. Consistent with this idea, SPRTN depletion stabilized methylated M.HpaII and abolished the formation of the degradation fragment (Figure 4D,

Figure 2. SPRTN and the Proteasome Are Recruited to a DPC Plasmid during Replication

(A) Depiction of replication, recovery, and analysis of pDPC^{2xLead} by PP-MS. To monitor the progress of the repair reaction, pDPC^{2xLead} was replicated in the presence of [α-^{32}P]dATP, and replication intermediates were analyzed by agarose gel electrophoresis (lower autoradiograph). In parallel, plasmids were isolated together with the bound proteins by a LacI pull-down (Budzowska et al., 2015) and analyzed by label-free MS.

(B) Heatmap showing the mean of the Z scored $\log_2$ label-free quantitation LFQ intensity from four biochemical replicates of pCTRL and pDPC^{2xLead}. Geminin was added to block replication where indicated.

(C) Analysis of protein recruitment to pDPC^{2xLead} compared to pCTRL. Both plasmids were recovered at 40 min. The volcano plot shows the mean difference of the protein intensity plotted against the p value calculated by a modified, one-sided t test. Full results are reported in Tables S1 and S2.

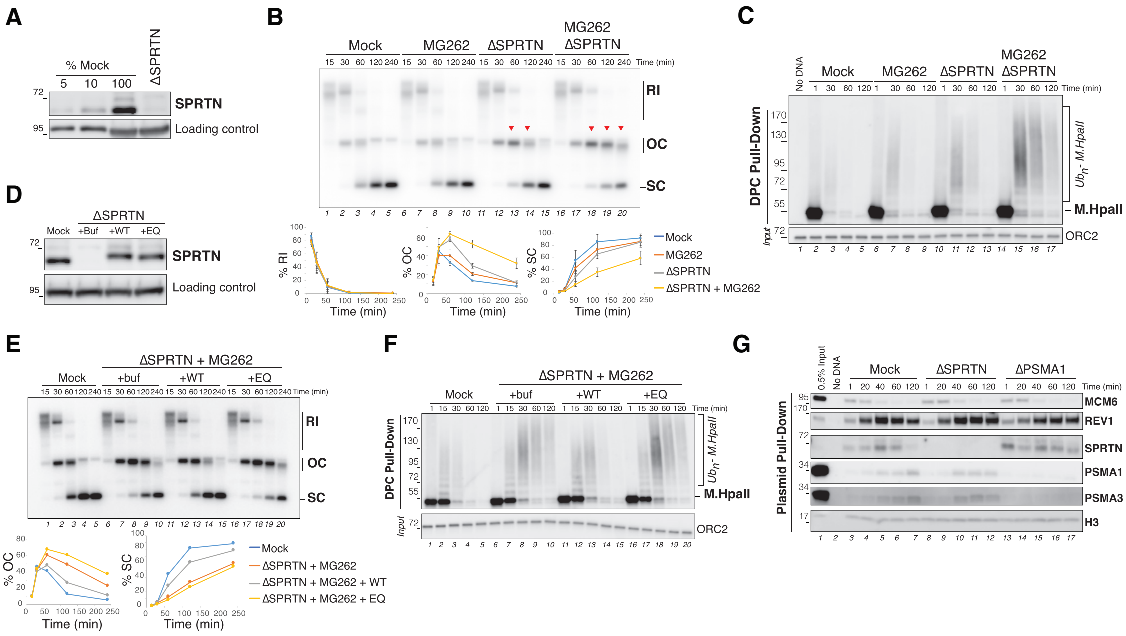

Figure 3. SPRTN and the Proteasome Degrade DPCs during Replication

(A) Mock-depleted and SPRTN-depleted egg extracts were blotted with SPRTN and MCM6 (loading control) antibodies.

(B) The extracts from (A) were used to replicate pDPC^{2xLead} in the presence of [α-^{32}P]dATP. MG262 at 200 μM was added where indicated. Samples were analyzed by native agarose gel electrophoresis. Red arrowheads indicate the accumulation of OC repair intermediates. Note that OC molecules that accumulate are subjected to 5′ to 3′ end resection and smear-down on the gel (lanes 14, 19, and 20;Duxin et al., 2014). Replication intermediates (RI), open circular (OC), and supercoiled species (SC) were quantified as a percentage of total lane signal (lower graphs). The mean percentages across three independent experiments are plotted, with error bars representing the SD.

(C) DPCs from (B) were recovered and monitored as in Figure 1B.

(D) Mock-depleted and SPRTN-depleted egg extracts were blotted with SPRTN and MCM6 (loading control) antibodies. SPRTN-depleted extracts were supplemented with either buffer (+Buf), recombinant FLAG-SPRTN (+WT), or recombinant catalytically inactive FLAG-SPRTN E89Q (+EQ).

(E) The extracts from (D) were used to replicate pDPC^{2xLead} in the presence of [α-^{32}P]dATP. MG262 at 200 μM was added where indicated. Samples were analyzed and quantified as in (B). The quantification of a representative biological replicate is shown.

(F) DPCs from (E) were monitored as in Figure 1B.

(G) Mock-depleted, SPRTN-depleted, or proteasome subunit α type-1 (PSMA1)-depleted extracts were used to replicate pDPC^{2xLead}. Plasmids were recovered, and protein-recruitment to the plasmid was monitored with the indicated antibodies (Budzowska et al., 2015).

lanes 6–10). This defect was reversed by SPRTN-WT but not SPRTN-EQ (Figure 4E, lanes 6–7 and 14–15). Conversely, MG262 did not prevent me-DPC proteolysis (Figures S4A and S4B). Consistent with the absence of DPC proteolysis, SPRTN depletion also caused a marked stabilization of OC intermediates during the replication of pme-DPC^{2xLead} and a corresponding delay in the generation of replicated SC molecules (Figure 4F, lanes 16–20). We conclude that SPRTN has a unique ability to degrade non-ubiquitylated DPCs. In contrast, DPC ubiquitylation is essential to target the proteasome.

SPRTN Requires Its Ubiquitin Binding Motifs to Degrade DPCs

We next explored the importance of the protein-interacting regions of SPRTN. In addition to its SprT metalloprotease domain, SPRTN contains C-terminal p97 (SHP), proliferating cell nuclear antigen (PCNA) (PIP), and ubiquitin (UBZ)-interacting regions (Figure S4C). We generated recombinant SPRTN with mutated SHP, PIP, or UBZ domains (Figures S4D and S4E; Davis et al., 2012; Mosbech et al., 2012) and tested their activity in pme-DPC^{2xLead} replication. Whereas SPRTN depletion blocked degradation of me-DPCs, re-addition of SPRTN-SHP* or SPRTN-PIP* reverted this effect (Figure 4E, lanes 8–9 and 10–11), suggesting that interactions with p97 and PCNA are not essential for the function of SPRTN as a DPC protease. In contrast, SPRTN-UBZ* failed to restore DPC proteolysis after SPRTN depletion (Figure 4E, lanes 12–13). Efficient generation of SC repair products was likewise supported by SPRTN-SHP* and SPRTN-PIP*, but not by SPRTN-UBZ* (Figure S4F). The role of SPRTN ubiquitin binding domains was confirmed by deleting both C-terminal UBZ domains (SPRTN 1–435), which also resulted in defective DPC degradation and replication (Figures S4G and S4H). In summary, our data demonstrate that SPRTN can act on DPCs in the absence of DPC ubiquitylation. However, SPRTN activity is still dependent on its ubiquitin-interacting motifs, suggesting that SPRTN is recruited by a ubiquitylated protein other than the DPC itself.

SPRTN-Mediated DPC Proteolysis Ensures Efficient TLS

As shown above, SPRTN depletion leads to a transient accumulation of OC intermediates during the replication of a DPC plasmid. To determine how SPRTN depletion affects replication across a

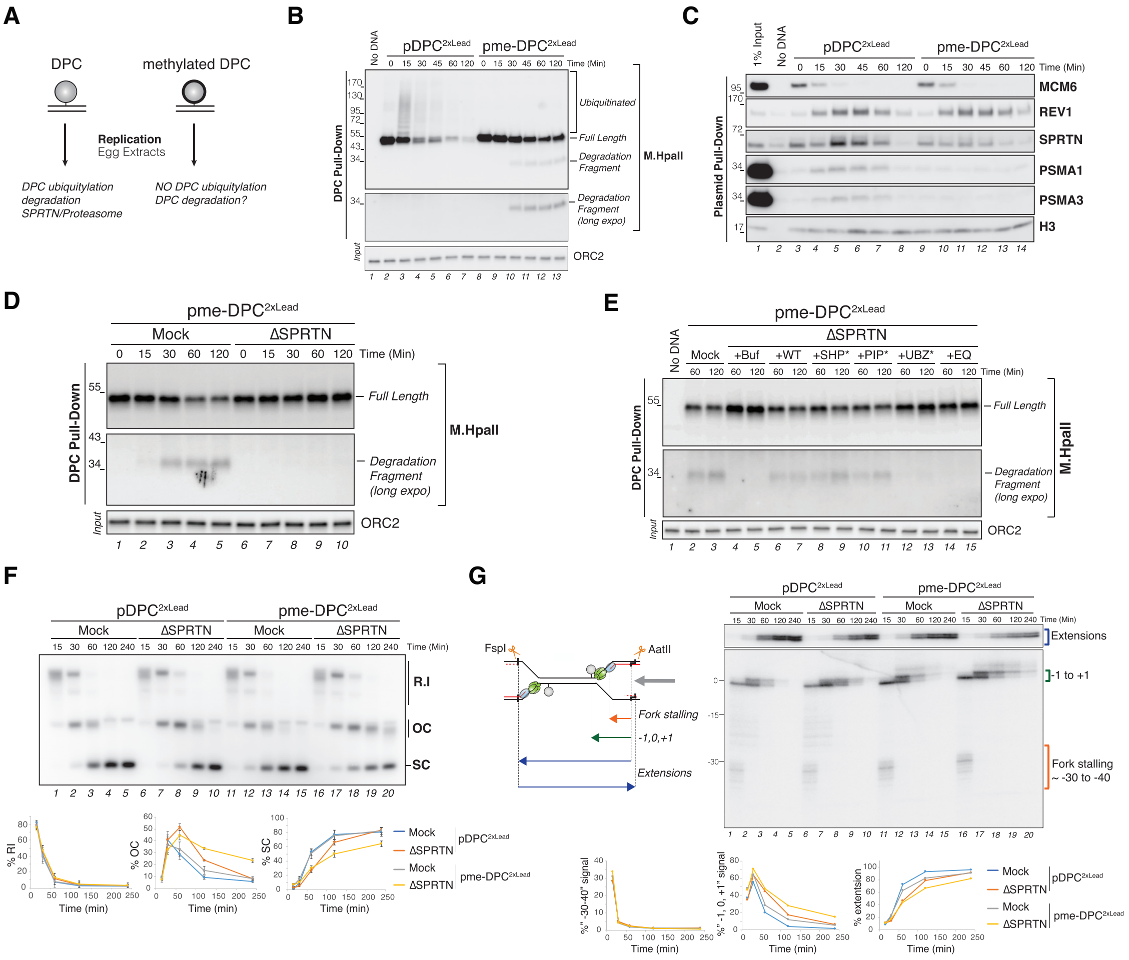

Figure 4. SPRTN, but Not the Proteasome, Can Degrade Non-ubiquitylated DPCs

(A) Strategy to address the role of DPC ubiquitylation via reductive methylation of the DPC.

(B) pDPC^{2xLead} and pme-DPC^{2xLead} were replicated in egg extracts, and DPCs were monitored as in Figure 1B. Note the concomitant disappearance of full-length M.HpaII and appearance of a degradation product during replication of pme-DPC^{2xLead}. Both a long and a short exposure of the M.HpaII blot are shown.

(C) pDPC^{2xLead} and pme-DPC^{2xLead} were replicated in egg extracts, and recruitment of the indicated proteins to the plasmid was monitored as in Figure 3G.

(D) pme-DPC^{2xLead} was replicated in mock-depleted or SPRTN-depleted egg extracts. DPCs were monitored as in Figure 1B.

(E) pme-DPC^{2xLead} was replicated in mock-depleted and SPRTN-depleted extracts. SPRTN-depleted extracts were supplemented with either buffer (+buf), or recombinant FLAG-SPRTN variants (see Figure S4D). DPCs were monitored as in Figure 1B.

(F) pDPC^{2xLead} and pme-DPC^{2xLead} were replicated in mock-depleted or SPRTN-depleted extracts. Samples were analyzed as in Figure 3B. The mean of three independent experiments is quantified. Error bars represent the SD.

(G) Samples from (F) were digested with FspI and AatII and separated on a denaturing polyacrylamide gel. The schematic depicts the nascent leading strands and extension products liberated by FspI and AatII digestion (green hexamer, CMG helicase; red lines, nascent DNA). The locations of the corresponding bands on the gel are indicated by brackets. The −30 to −40 species, the −1,0,1 species, and extension products were quantified and plotted below. Quantification of each species is plotted as a percentage of the entire signal of the lane. The quantification of a representative biological replicate is shown.

DPC, we first replicated pDPC^{2xLead} and analyzed nascent leading strands on a denaturing polyacrylamide gel (Figure 4G) (Duxin et al., 2014). In mock-depleted extracts, the nascent leading strand first paused 30 to 40 nt upstream of the DPC due to the footprint of CMG, which stalled at the DPC (fork stalling) (Figure 4G, lanes 1–5). Subsequently, the nascent leading strand advanced and stalled again at the lesion site (−1, 0, +1) before being extended past the lesion via TLS polymerases REV1-Polζ (extension) (Figures 4G and S4I for the annotation of −1, 0, and +1 products). SPRTN depletion did not inhibit leading strands from reaching the lesion, but it did prolong the stalling observed at −1, 0, and +1 positions by ~30 min, which correlated with a delay in the appearance of the extension products (Figures 4G, lanes 6–10, S4K, and S4M). This TLS defect was rescued by SPRTN-WT but not SPRTN-EQ, which further inhibited synthesis across the lesion (Figures S4J and S4K). The inhibitory effect of SPRTN depletion was neutralized by pre-treatment of the DPC with proteinase K (Figures S4L–S4N). We conclude that SPRTN-mediated DPC proteolysis facilitates TLS past the lesion. In the absence of SPRTN, the DPC is still degraded by the proteasome, but TLS across the resulting peptide adduct generated by the proteasome is likely not as efficient.

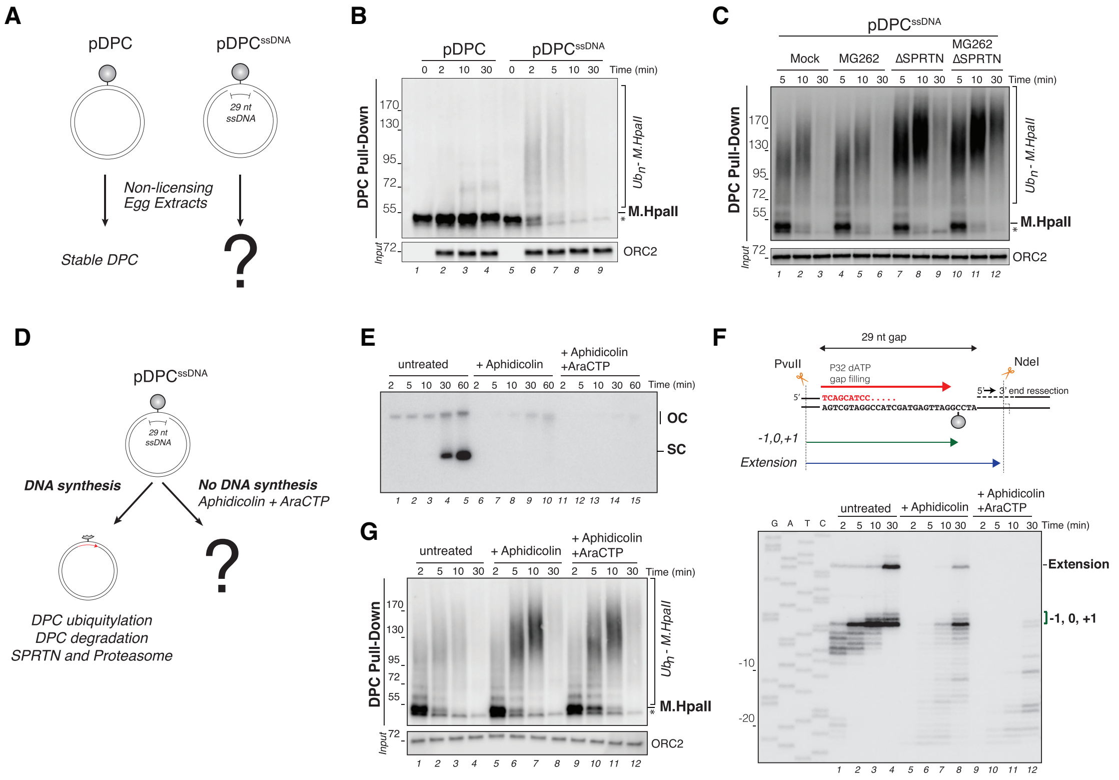

Figure 5. DPC Ubiquitylation and Degradation Can Occur in the Absence of the Replisome

(A) Schematic comparing pDPC and pDPC^{ssDNA} in non-licensing egg extracts.

(B) pDPC and pDPC^{ssDNA} were incubated in non-licensing egg extracts. DPCs were recovered and monitored as in Figure 1B. Note that time 0 was withdrawn before incubating plasmids in egg extracts, which explains the absence of ORC2 input in lanes 1 and 5.

(C) pDPC^{ssDNA} was incubated in mock-depleted and SPRTN-depleted non-licensing extracts in the presence of 200 μM MG262 where indicated. DPCs were monitored as in Figure 1B.

(D) Schematic comparing the fate of pDPC^{ssDNA} in the presence or absence of gap-filling synthesis.

(E) pDPC^{ssDNA} was incubated in non-licensing egg extracts in the presence of [α-^{32}P]dATP. Extracts were supplemented with 700 μM aphidicolin and 1 mM araCTP where indicated. Samples were analyzed as in Figure 3B.

(F) Samples from (E) were digested with PvuII and NdeI and separated on a denaturing polyacrylamide gel. The different extension products are depicted in the upper scheme.

(G) Samples from (E) were used to monitor DPC ubiquitylation and degradation as in Figure 1B.

We next investigated the role of SPRTN in facilitating replication across the methylated DPC, which cannot be acted on by the proteasome. In this context, SPRTN depletion also induced a marked TLS defect, as seen by the prolonged stalling of leading strands at the DPC (Figure 4G, lanes 16–20). Despite the absence of DPC proteolysis, the approach of leading strands to the lesion was unaffected (Figure 4G, see disappearance of −30 to −40 products), demonstrating that CMG disappears from the DPC on schedule. This loss of the CMG footprint is due to CMG bypass of the intact DPC (Sparks et al., 2018). Thus, in the presence and absence of the proteasome pathway, SPRTN is required to facilitate TLS across the lesion.

SPRTN and the Proteasome Can Degrade DPCs in the Absence of the Replisome

We next addressed how SPRTN and proteasome activities are coupled to DNA replication. In one scenario, the replisome recruits or activates these proteases. Alternatively, DNA replication generates a structure that targets the proteases to DPCs. To distinguish between the two models, we tested whether ssDNA could trigger DPC degradation in the absence of the replisome. To this end, we generated a plasmid in which M.HpaII is linked to one strand across from a 29-nt gap (pDPC^{ssDNA}; Figure 5A). We then monitored M.HpaII degradation on pDPC^{ssDNA} in extracts that do not support MCM2–7 loading or replication initiation (non-licensing extracts). In this setting, pDPC^{ssDNA} triggered rapid polyubiquitylation and degradation of M.HpaII, whereas pDPC did not (Figure 5B, lanes 1–4 and 6–9). In addition, on pDPC^{ssDNA}, ubiquitylated M.HpaII species were stabilized most by the combined inhibition of the proteasome and depletion of SPRTN (Figures 5C, lanes 10–12, and S5A–S5C). Therefore, both SPRTN and the proteasome can degrade DPCs in the absence of a full replisome when the lesion resides on ssDNA. Consistent with this conclusion, purified Wss1 and SPRTN are activated by ssDNA (Balakirev et al., 2015; Stingele et al., 2016).

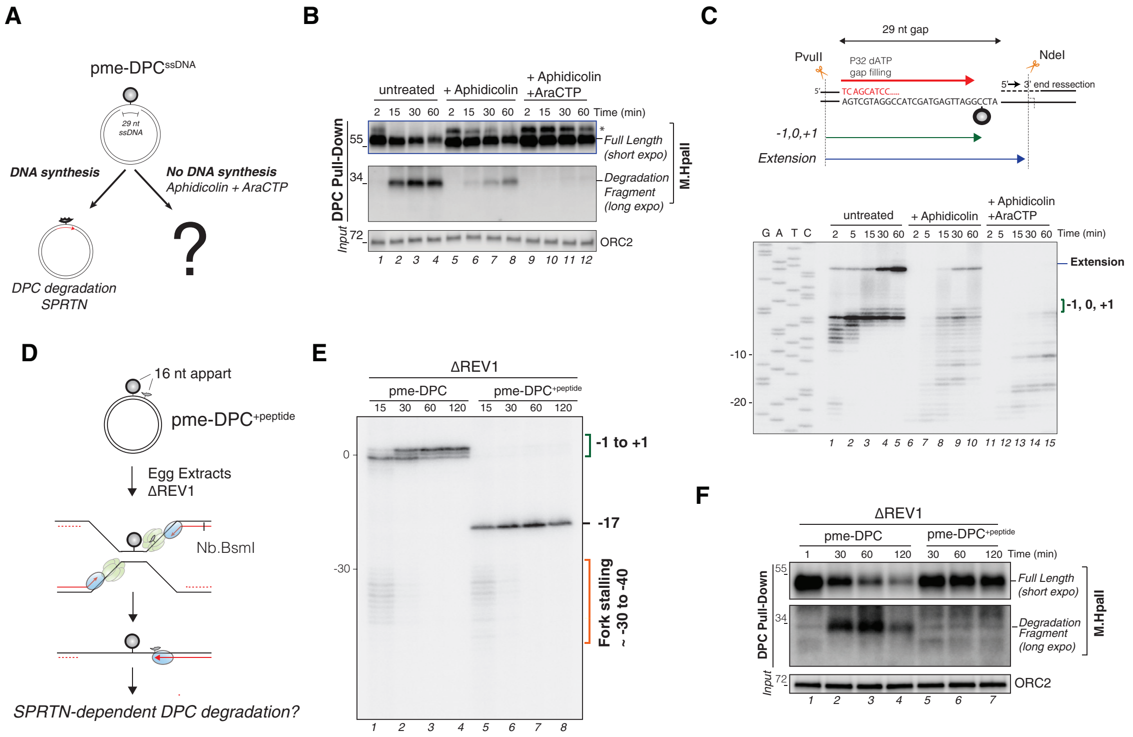

Figure 6. SPRTN-Dependent DPC Degradation Requires Nascent Strand Extension to the Lesion

(A) Schematic comparing the fate of pme-DPC^{ssDNA} in the presence and absence of gap-filling synthesis.

(B) pme-DPC^{ssDNA} was incubated in non-licensing egg extracts supplemented with 700 μM aphidicolin and 1 mM araCTP where indicated. DPC degradation was monitored as in Figure 1B. The asterisk denotes a crosslinked methyl-M.HpaII species generated on the GAP substrate, likely caused by the incomplete degradation of ssDNA by benzonase.

(C) Samples from (B) were analyzed as in Figure 5F.

(D) Depiction of pme-DPC$^{+peptide}$ replication.

(E) pme-DPC and pme-DPC$^{+peptide}$ were replicated in REV1-depleted extracts in the presence of [α-^{32}P]dATP. Samples were digested with Nb.BsmI, which cuts the leftward leading strand, as depicted in (D). Nascent leading strands were then separated on a polyacrylamide denaturing gel.

(F) Samples from (E) were used to monitor DPC degradation as in Figure 1B.

Polymerase Extension Controls SPRTN-Mediated DPC Degradation

When pDPC^{ssDNA} was incubated in non-licensing extracts, we detected a small amount of DNA synthesis that was absent on pDPC (Figures S5D and S5E). This synthesis reflected extension of the free 3′ end to the DPC, followed by TLS past the lesion (Figure S5F). We asked whether this gap-filling synthesis is required to trigger DPC ubiquitylation and degradation (Figure 5D). To this end, we treated egg extracts with aphidicolin, which greatly diminished gap filling, or with a combination of aphidicolin and the chain terminator *ara*-cytidine-5′-triphosphate (araCTP), which inhibited gap filling almost completely (Figures 5E and 5F). As shown in Figure 5G, aphidicolin or aphidicolin and araCTP impaired DPC proteolysis, although M.HpaII ubiquitylation occurred normally. Thus, efficient DPC proteolysis but not DPC ubiquitylation requires gap-filling synthesis.

Given that DPC degradation but not ubiquitylation was delayed, we reasoned that DPC proteolysis by SPRTN but not the proteasome requires gap-filling synthesis. To monitor SPRTN activity, we examined a gapped substrate containing methylated M.HpaII (Figure 6A). As seen during DNA replication, methylated M.HpaII underwent ubiquitylation-independent degradation, giving rise to the SPRTN-dependent proteolytic fragment (Figure S5G). The efficiency of proteolysis and appearance of the DPC fragment correlated with the amount of gap-filling synthesis, as these were partially inhibited by aphidicolin (Figures 6B, lanes 5–8, and 6C, lanes 6–10) and completely blocked by the combination of aphidicolin and araCTP (Figures 6B, lanes 9–12, and 6C, lanes 11–15). In contrast, if aphidicolin and araCTP were added at 3 min, when the majority of 3′ ends had reached the crosslink (Figures S6A–S6C) but before the marked accumulation of the DPC fragment, M.HpaII degradation occurred normally (Figure S6D, lanes 5–8). We conclude that nascent strand extension to the immediate vicinity of the DPC is a prerequisite to trigger SPRTN-mediated DPC degradation in the context of pDPC^{ssDNA}.

To test whether strand extension triggers SPRTN activity at a replication fork, a short peptide adduct was placed 16 nt upstream of the methylated DPC, yielding pme-DPC$^{+peptide}$ (Figure 6D). The peptide should inhibit polymerase extension while having no impact on CMG progression. To ensure that no leading strands reached the DPC, pme-DPC$^{+peptide}$ was replicated in REV1-depleted extracts. A matched pme-DPC substrate lacking the peptide served as a control. As seen in Figure 6E, after first

pausing at the −30 to −40 positions due to CMG collision with the DPC, leading strands on pme-DPC$^{+peptide}$ were extended but then permanently stalled at the upstream peptide adduct (lanes 5–8, −17 position). Under these conditions, methylated M.HpaII persisted, and the SPRTN-mediated proteolytic fragment never appeared (Figure 6F, lanes 5–7). In contrast, M.HpaII proteolysis proceeded normally on pme-DPC (Figure 6F, lanes 1–4), where leading strands were allowed to reach the DPC (Figure 6E, lanes 1–4). These results demonstrate that in the context of replication, CMG-DPC collision is insufficient to activate SPRTN. Instead, SPRTN activity is strictly dependent on the subsequent extension of a nascent strand to the lesion, which can only occur once the CMG helicase has dissociated or moved past the protein adduct.

Having defined the requirements for SPRTN proteolysis, we repeated the experiment with unmethylated M.HpaII, which should be ubiquitylated and therefore degraded by the proteasome in the absence of polymerase extension. To this end, we replicated pDPC$^{+peptide}$ in REV1-depleted extracts in the presence or absence of MG262 (Figure S6E). Unmethylated M.HpaII underwent rapid polyubiquitylation and degradation, although leading strands never reached the lesion (Figures S6F and S6G). In this context, MG262 stabilized polyubiquitylated M.HpaII (Figure S6G). Thus, DPC ubiquitylation and degradation by the proteasome do not require polymerase advancement to the lesion site.

TRAIP Stimulates DPC Ubiquitylation and Proteasome Targeting

Finally, we addressed which E3 ligase ubiquitylates the DPC. Our PP-MS analysis identified several ubiquitin ligases that were enriched on replicating pDPC^{2xLead} (Figure 7A). Among these, TRAIP was a good candidate because it was strongly enriched early in the reaction at the onset of DPC ubiquitylation (Figure 7A, 12 and 20 min), and its recruitment to chromatin depended on replication (Figure 7A, ±Geminin). Moreover, TRAIP-deficient cells exhibit impaired replication fork progression upon stress and sensitivity to crosslinking agents (Feng et al., 2016; Harley et al., 2016; Hoffmann et al., 2016). To clearly monitor DPC ubiquitylation and proteasome-mediated DPC degradation, we performed experiments in the absence of SPRTN. In this setting, TRAIP depletion delayed DPC ubiquitylation and degradation by 10–15 min compared to the mock reaction (Figures 7B, 7C, lanes 1–12, S7A, and S7B) without affecting DNA replication kinetics (Figure S7C). Both DPC ubiquitylation and proteolysis were largely restored by recombinant wild-type (WT) TRAIP (Figures 7B and 7C, lanes 13–18), but not a TRAIP mutant harboring an amino acid substitution in the RING domain (R18C) (Figures 7B and 7C, lanes 19–24) that causes primordial dwarfism (Harley et al., 2016). In contrast, when pDPC^{ssDNA} was incubated in non-licensing extracts depleted of TRAIP, M.HpaII ubiquitylation was unaffected (Figure S7D), suggesting the existence of a second E3 ligase that operates on the DPC in the context of ssDNA. These results indicate that TRAIP promotes DPC ubiquitylation and proteolysis by the proteasome during replication.

We recently demonstrated that in the absence of DPC proteolysis, CMG bypasses an intact DPC, and this bypass is required for efficient DPC proteolysis (J.L.S., unpublished data). Moreover, we showed that bypass requires the DNA helicase regulator of telomere elongation helicase 1 (RTEL1). In the absence of RTEL1, ubiquitin chains form on the DPC with normal kinetics, but they are shorter, indicating that the DPC can be ubiquitylated even before CMG has bypassed the lesion (Figure S7E, compare lanes 2–3 and 8–9 and J.L.S., unpublished data). Based on these findings, we postulated that TRAIP promotes DPC ubiquitylation upon replisome collision with the DPC and that subsequent CMG bypass generates ssDNA surrounding the DPC, stimulating DPC ubiquitylation by the second, ssDNA-activated E3 ligase. If this is the case, then the dependence of DPC ubiquitylation on TRAIP should be more pronounced in the absence of RTEL1. In RTEL1- and SPRTN-depleted extracts, TRAIP depletion delayed M.HpaII ubiquitylation by up to 30 min (Figures 7D, lanes 7–12, and S7E), and this effect was rescued by TRAIP WT but not TRAIP R18C (Figure 7D, lanes 13–24). Consistent with the inhibition in DPC ubiquitylation, TRAIP depletion in the absence of RTEL1 delayed proteasome recruitment to chromatin (Figure 7E, compare lanes 3–8 and lanes 9–14), which was partially rescued by TRAIP WT (Figure 7E, lanes 15–20). While DNA replication kinetics were unaffected by TRAIP depletion (Figures S7C and S7F), both in the presence and absence of RTEL1, TRAIP depletion delayed the disappearance of the CMG footprint at the DPC, which is consistent with a defect in CMG bypass (Figures 7F, lanes 1–12 and 13–24, and 7G). This effect on CMG bypass was not observed in methylated DPC (Figure S7G), strongly suggesting that the relevant target of TRAIP ubiquitylation is the DPC. We conclude that DPC ubiquitylation and proteasome targeting occurs in two stages: first, when forks collide with a DPC, TRAIP-dependent DPC ubiquitylation promotes CMG bypass, and second, CMG bypass creates ssDNA surrounding the DPC that enables further DPC ubiquitylation by a second, unknown E3 ubiquitin ligase.

DISCUSSION

We previously demonstrated that DPCs are degraded in a replication-dependent process, but how this occurs was unclear (Duxin et al., 2014). Using a newly developed PP-MS proteomic workflow, we identified SPRTN and the proteasome as two proteases that are recruited to a DPC lesion during DNA replication. We further demonstrate that SPRTN and the proteasome operate in different pathways that are differentially activated by DNA replication (Figure 7H). The implications of these findings are discussed below.

Polymerase Approach Targets SPRTN

Our results raise the possibility that the stalling of a DNA polymerase at a DPC targets and activates SPRTN. Previous studies reported direct interactions between SPRTN and DNA polymerase delta subunit 3 (POLD3), one of the accessory subunits of DNA Polδ (Ghosal et al., 2012; Kim et al., 2013), suggesting that Polδ may direct SPRTN to the DPC. To date, we have not been able to achieve sufficient depletion of Polδ from egg extracts to prevent gap-filling synthesis (data not shown), precluding a direct test of this model. Purified SPRTN is activated

CellPress

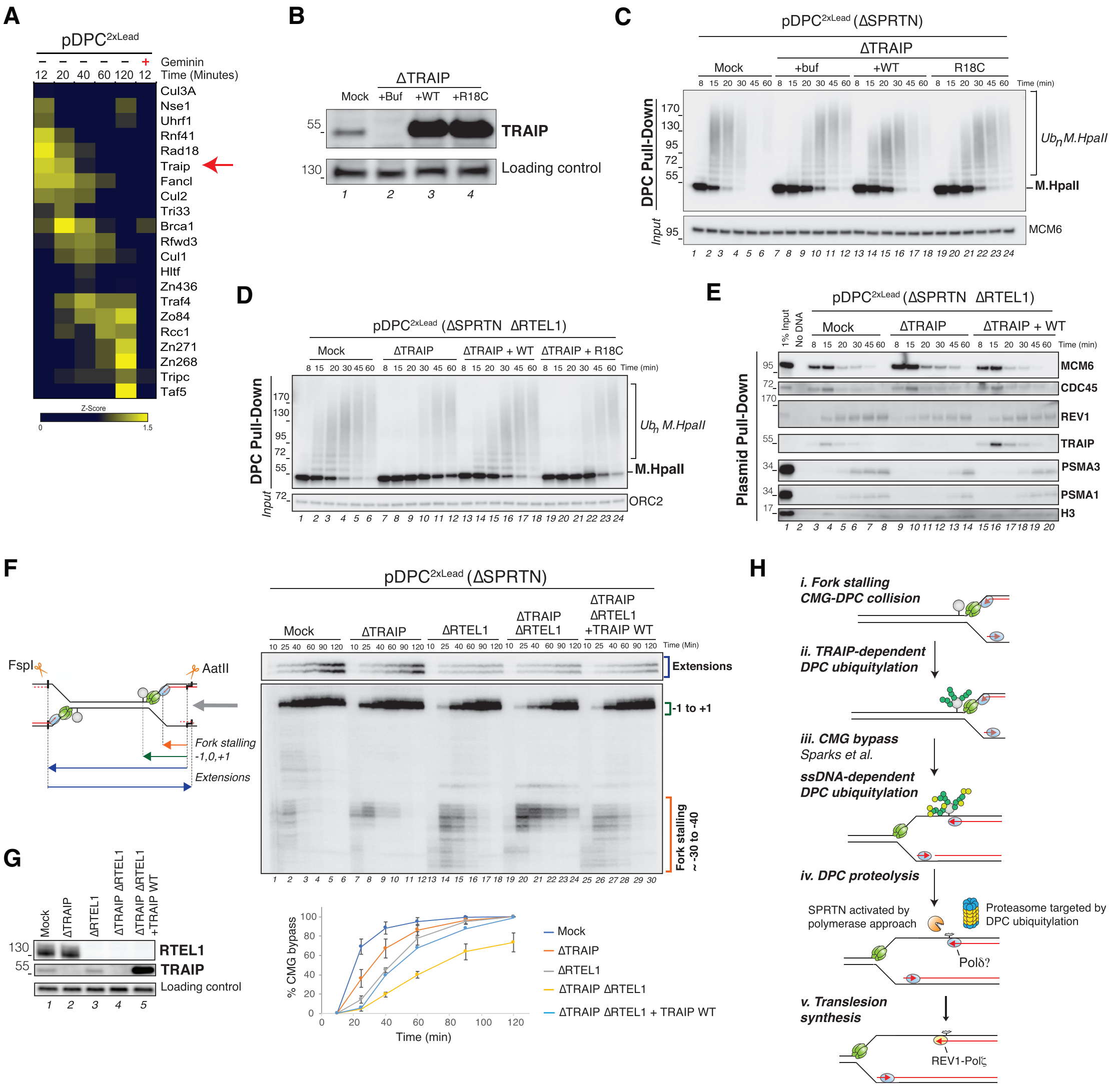

Figure 7. TRAIP Ubiquitin Ligase Stimulates DPC Ubiquitylation and Proteasome Targeting

(A) Heatmap showing the mean of the *Z* scored log2 LFQ intensity of potential E3 ubiquitin ligases. Proteins with similar intensities in the geminin or mock control lacking the DNA substrate were excluded.

(B) Extracts were depleted with SPRTN and either control immunoglobulin G (IgG) or TRAIP antibodies and blotted for TRAIP and RTEL1 (loading control). TRAIP-depleted extracts were supplemented with buffer (+Buf), recombinant TRAIP(WT), or TRAIP(R18C).

(C) Extracts from (B) were used to replicate pDPC^{2xLead}. DPCs were monitored as in Figure 1B.

(D) SPRTN- and RTEL1-depleted extracts were either mock depleted or TRAIP depleted. TRAIP-depleted extracts were supplemented with buffer (+Buf), recombinant TRAIP(WT), or TRAIP(R18C). These extracts were used to replicate pDPC^{2xLead}, and DPCs were monitored as in Figure 1B.

(E) The indicated extracts were used to replicate pDPC^{2xLead}. Recruitment of the indicated proteins to the plasmid was monitored as in Figure 3G.

(F) Extracts described in (D) were used to replicate pDPC^{2xLead} in the presence of [α-^{32}P]dATP, and nascent strand intermediates were analyzed as in Figure 4G. CMG bypass was measured based on the disappearance of the −30 to −40 CMG footprint (Sparks et al., 2018). The mean of three independent experiments is graphed for mock-, TRAIP-, and RTEL1-TRAIP-depleted samples. Error bars represent the SD. The TRAIP-RTEL1-depleted samples supplemented with TRAIP(WT) represents the mean of two experiments and plotted without error bars.

(G) Samples from (F) were blotted with TRAIP, RTEL1, or SLD5 (loading control) antibodies.

(H) Model for replication-coupled DPC proteolysis in *Xenopus* egg extracts. Black lines, parental DNA; red lines, nascent DNA; green hexamers, CMG helicase; blue spheres, replicative polymerases; yellow spheres, TLS polymerase; gray sphere, DPC; orange, SPRTN; yellow and blue, the proteasome; dark green, TRAIP-dependent ubiquitin chains; light green, ubiquitin chains deposited by a second E3 ligase activated by ssDNA.

by DNA *in vitro* (Lopez-Mosqueda et al., 2016; Mórocz et al., 2017; Stingele et al., 2016; Vaz et al., 2016), with ssDNA being particularly potent (Stingele et al., 2016). We therefore speculate that SPRTN activation requires the presence of a DNA polymerase on one side of a DPC and a short tract of ssDNA on the other side. This dual requirement would specifically target SPRTN to

DPCs during replication and avoid indiscriminate destruction of replisome components or other chromatin proteins.

A model of SPRTN activation by polymerase-DPC collision has numerous implications. First, because CMG blocks the ability of leading strands to reach a DPC on the leading strand template, our data imply that proteolysis by SPRTN can only occur if CMG is no longer present in front of the DPC (Figure 7F). Accordingly, we show that the CMG helicase readily bypasses leading strand DPCs and that this process requires the helicase activity of RTEL1 (Sparks et al., 2018). Consistent with a requirement for leading strand extension in SPRTN activity, in the absence of RTEL1-mediated DPC bypass, DPC proteolysis by SPRTN is impaired. Second, SPRTN-dependent DPC proteolysis can likely be uncoupled from the replication fork. Supporting this idea, we show that SPRTN efficiently degrades a DPC linked to ssDNA in the absence of the replisome via a process that mimics post-replicative repair (Figures 6A–6C). By restricting SPRTN to act behind the replication fork, cells ensure that irreplaceable replisome factors such as CMG are not accidentally degraded during replication. Finally, SPRTN activation by polymerase-DPC collision suggests a common mechanism of DPC degradation on the leading and lagging strands; if Polε remains associated with CMG during DPC bypass, this would liberate the leading strand for Polδ recruitment. In this way, both leading- and lagging-strand DPC proteolysis would be triggered by the collision of Polδ with the adduct.

SPRTN contains C-terminal domains that interact with p97, PCNA, and ubiquitin, but their importance in SPRTN activity is unclear. While some reports suggested that these domains are not essential (Maskey et al., 2014; Stingele et al., 2016), more recent evidence indicates that both the ubiquitin and PCNA binding interactions of SPRTN are important for its role as a DPC protease in human cells (Mórocz et al., 2017). In *Xenopus* egg extracts, the ubiquitin binding domain of SPRTN is important for efficient DPC proteolysis, even in the absence of DPC ubiquitylation, suggesting that SPRTN interacts with another ubiquitylated protein near the lesion. Given our model that polymerase-DPC collision triggers SPRTN activity, a possible candidate is PCNA, which is ubiquitylated during post-replicative repair (Hoege et al., 2002). Consistent with this idea, RAD18 is epistatic to SPRTN for DPC repair (Mórocz et al., 2017). Although the PIP motif was not required for SPRTN activity in egg extracts, this may reflect the presence of tandem UBZ domains in *Xenopus* SPRTN that could compensate for reduced PCNA binding.

DPC Ubiquitylation Promotes Proteasome-Mediated DPC Degradation

It was previously proposed that the proteasome can degrade DPCs, but evidence that this process occurs during DNA replication was lacking. Our work in *Xenopus* egg extracts shows that replication triggers rapid polyubiquitylation of a type I DPC. When DPC ubiquitylation is prevented via lysine methylation of the DPC, proteasome recruitment is abolished, strongly supporting a role for DPC ubiquitylation in proteasome targeting. Although the interplay between TRAIP and the second E3 ubiquitin ligase and their specific functions in proteasome targeting are unclear at present, a comprehensive model for replication-coupled DPC ubiquitylation is starting to emerge (Figure 7H). Our data suggest that one of the earliest events following the replisome-DPC encounter is TRAIP-mediated ubiquitylation of the DPC (Figure 7H, i–ii). Three observations support this notion. First, TRAIP is enriched on the DPC plasmid early in the reaction before CMG bypass (already present at 12 min, before CMG bypass is observed [Figure S2C]). Second, TRAIP-dependent DPC ubiquitylation is independent of RTEL1 and therefore does not require CMG bypass (Figure 7D). Third, TRAIP depletion delayed CMG bypass, and this effect was abrogated by the methylation of the DPC (Figures 7F and S7G). Following CMG bypass, ssDNA surrounding the DPC likely stimulates the activity of the second E3 (Figure 7H, iii). Supporting this idea, we show here that when the DPC is linked to ssDNA, DPC ubiquitylation and proteasome-dependent DPC degradation occurs independently of the replisome or TRAIP (Figures 5 and S7D). Whether TRAIP-dependent DPC ubiquitylation promotes proteasome targeting independently of CMG bypass is an interesting question. For example, TRAIP ubiquitylation of impassable DPCs may gradually stimulate their processing by the proteasome to facilitate CMG bypass. The role of TRAIP in promoting efficient CMG bypass predicts that TRAIP should also be required for optimal SPRTN activity. TRAIP mutations in humans induce microcephalic primordial dwarfism (Harley et al., 2016). Future work will be required to address whether this phenotype is attributable to defective replication bypass and degradation of DPCs or other functions of TRAIP.

SPRTN and the Proteasome Are Not Redundant DPC Proteases

How do our findings relate to the defective replication fork progression and formaldehyde sensitivity observed in SPRTN-deficient cells (Lessel et al., 2014; Vaz et al., 2016)? We showed that SPRTN depletion delays TLS, indicating that SPRTN and the proteasome are not redundant. We speculate that SPRTN is able to degrade DPCs to peptide adducts that are sufficiently small for efficient TLS. The protease active site of Wss1 is highly solvent exposed, suggesting that it should be able to cleave DPCs close to the DNA attachment site (Stingele et al., 2016). In contrast, the active sites of the proteasome are buried inside the 20S core particle. Threading of the unfolded DPC through the cylindrical 20S particle would likely be interrupted upon its encounter with the attached DNA, resulting in a larger peptide adduct. Thus, when DPCs are channeled into the proteasomal pathway, SPRTN may still be required in a second proteolytic step to reduce the peptide adduct to a few amino acids. Our findings predict that in SPRTN-deficient cells, CMG becomes uncoupled from the leading strand due to defective TLS. In bacteria, helicase uncoupling greatly slows the rate of DNA unwinding (Kim et al., 1996), and this is also true in vertebrates (J.L.S., unpublished data). Therefore, we speculate that defective fork progression in SPRTN-deficient cells reflects slow unwinding by uncoupled CMG. Defective TLS may also contribute to the formaldehyde sensitivity of SPRTN-deficient cells, as REV1- and REV3-deficient cells are also sensitive to formaldehyde treatment (Ridpath et al., 2007).

DPCs are expected to exhibit great variability in size, structure, and attachment chemistry. While agents such as

formaldehyde crosslink proteins to duplex DNA, abortive reactions by topoisomerase form DPCs that are flanked by a DNA break. Hence, while both SPRTN and the proteasome readily degrade M.HpaII, it is conceivable that other crosslinked proteins are preferentially processed by one or the other protease. For example, SPRTN is expected to be particularly critical for DPCs that lack available lysines and therefore cannot be ubiquitylated, which may account for the DPCs that accumulate in SPRTN-deficient cells (Stingele et al., 2016; Vaz et al., 2016). In contrast, the proteasome may be essential for very large DPCs that cannot be bypassed by the replication fork and require "pre-trimming" by the proteasome. Alternatively, the proteasome may be critical in removing DPCs flanked by DNA breaks outside of replication or when DPCs are encountered by the transcription machinery. The use of at least two DPC proteases with orthogonal mechanisms and triggers represents a versatile system to degrade a wide variety of DPCs.

STAR★METHODS

Detailed methods are provided in the online version of this paper and include the following:

- KEY RESOURCES TABLE
- CONTACT FOR REAGENT AND RESOURCE SHARING
- EXPERIMENTAL MODEL AND SUBJECT DETAILS
- METHOD DETAILS
  - *Xenopus* Egg Extracts and DNA Replication
  - Preparation of DNA constructs
  - Antibodies and Immunodepletion
  - Nascent-Strand Analysis
  - Protein Expression and Purification
  - Plasmid Pull-Downs
  - DPC Pull-Downs
  - Plasmid Pull-down Mass Spectrometry (PP-MS)
- QUANTIFICATION AND STATISTICAL ANALYSIS
- DATA AND SOFTWARE AVAILABILITY

SUPPLEMENTAL INFORMATION

Supplemental Information includes seven figures and two tables and can be found with this article online at https://doi.org/10.1016/j.molcel.2018.11.024.

ACKNOWLEDGMENTS

We thank Peter Burgers, Jiri Lukas, Niels Mailand, and members of the Walter and Duxin laboratories for feedback on the manuscript. We thank Yoshiaki Azuma for the dnUBC9 protein and expression construct, and Igor Paron and Jürgen Cox for mass spectrometry support. The Novo Nordisk Foundation Center for Protein Research is supported financially by the Novo Nordisk Foundation (grant agreement NNF14CC0001). This project has received funding from the European Research Council (ERC) under the European Union's Horizon 2020 research and innovation program (grant agreement 715975). M.R. was supported by the Center for Integrated Protein Research Munich (CIPSM) and is currently supported by a grant from the German Research Foundation (Research Group FOR2800); R.A.W. was supported by postdoctoral fellowship 131415-PF-17-168-01-DMC from the American Cancer Society; J.L.S. was supported by a Damon Runyon Fellowship; and J.C.W. was supported by NIH grant HL98316. J.C.W. is an investigator of the Howard Hughes Medical Institute.

AUTHOR CONTRIBUTIONS

N.B.L. and A.O.G. performed most of the experiments; J.L.S. performed the experiments presented in Figures 7 and S7E–S7G; I.G. performed the experiments in Figures 1D, 1E, and S1F. The MS samples were prepared by J.P.D. and J.L.S. and run and analyzed by M.R. and M.M. R.A.W. cloned and purified TRAIP proteins. J.C.W. helped design and analyze experiments at early stages of the project. N.B.L., A.O.G., and J.P.D. designed and analyzed the experiments and prepared the manuscript. This project was initiated by J.P.D. in J.C.W.'s group and completed in J.P.D.'s laboratory.

DECLARATION OF INTERESTS

The authors declare no competing interests.

Received: March 9, 2018
Revised: September 20, 2018
Accepted: November 15, 2018
Published: December 27, 2018

REFERENCES

Azuma, Y., Arnaoutov, A., and Dasso, M. (2003). SUMO-2/3 regulates topoisomerase II in mitosis. J. Cell Biol. *163*, 477–487.

Baker, D.J., Wuenschell, G., Xia, L., Termini, J., Bates, S.E., Riggs, A.D., and O'Connor, T.R. (2007). Nucleotide excision repair eliminates unique DNA-protein cross-links from mammalian cells. J. Biol. Chem. *282*, 22592–22604.

Balakirev, M.Y., Mullally, J.E., Favier, A., Assard, N., Sulpice, E., Lindsey, D.F., Rulina, A.V., Gidrol, X., and Wilkinson, K.D. (2015). Wss1 metalloprotease partners with Cdc48/Doa1 in processing genotoxic SUMO conjugates. eLife *4*, e06763.

Barker, S., Weinfeld, M., and Murray, D. (2005). DNA-protein crosslinks: their induction, repair, and biological consequences. Mutat. Res. *589*, 111–135.

Budzowska, M., Graham, T.G.W., Sobeck, A., Waga, S., and Walter, J.C. (2015). Regulation of the Rev1-pol ζ complex during bypass of a DNA interstrand cross-link. EMBO J. *34*, 1971–1985.

Centore, R.C., Yazinski, S.A., Tse, A., and Zou, L. (2012). Spartan/C1orf124, a reader of PCNA ubiquitylation and a regulator of UV-induced DNA damage response. Mol. Cell *46*, 625–635.

Chen, L., MacMillan, A.M., Chang, W., Ezaz-Nikpay, K., Lane, W.S., and Verdine, G.L. (1991). Direct identification of the active-site nucleophile in a DNA (cytosine-5)-methyltransferase. Biochemistry *30*, 11018–11025.

Chválová, K., Brabec, V., and Kaspárková, J. (2007). Mechanism of the formation of DNA-protein cross-links by antitumor cisplatin. Nucleic Acids Res. *35*, 1812–1821.

Cox, J., and Mann, M. (2008). MaxQuant enables high peptide identification rates, individualized p.p.b.-range mass accuracies and proteome-wide protein quantification. Nat. Biotechnol. *26*, 1367–1372.

Davis, E.J., Lachaud, C., Appleton, P., Macartney, T.J., Näthke, I., and Rouse, J. (2012). DVC1 (C1orf124) recruits the p97 protein segregase to sites of DNA damage. Nat. Struct. Mol. Biol. *19*, 1093–1100.

Desai, S.D., Liu, L.F., Vazquez-Abad, D., and D'Arpa, P. (1997). Ubiquitin-dependent destruction of topoisomerase I is stimulated by the antitumor drug camptothecin. J. Biol. Chem. *272*, 24159–24164.

Dewar, J.M., Budzowska, M., and Walter, J.C. (2015). The mechanism of DNA replication termination in vertebrates. Nature *525*, 345–350.

Dewar, J.M., Low, E., Mann, M., Räschle, M., and Walter, J.C. (2017). $CRL2^{Lrr1}$ promotes unloading of the vertebrate replisome from chromatin during replication termination. Genes Dev. *31*, 275–290.

Dimova, N.V., Hathaway, N.A., Lee, B.-H., Kirkpatrick, D.S., Berkowitz, M.L., Gygi, S.P., Finley, D., and King, R.W. (2012). APC/C-mediated multiple monoubiquitylation provides an alternative degradation signal for cyclin B1. Nat. Cell Biol. *14*, 168–176.

Duxin, J.P., Dewar, J.M., Yardimci, H., and Walter, J.C. (2014). Repair of a DNA-protein crosslink by replication-coupled proteolysis. Cell *159*, 346–357.

Fang, F., and Newport, J.W. (1993). Distinct roles of cdk2 and cdc2 in RP-A phosphorylation during the cell cycle. J. Cell Sci. *106*, 983–994.

Feng, W., Guo, Y., Huang, J., Deng, Y., Zang, J., and Huen, M.S.-Y. (2016). TRAIP regulates replication fork recovery and progression via PCNA. Cell Discov. *2*, 16016.

Fu, Y.V., Yardimci, H., Long, D.T., Ho, T.V., Guainazzi, A., Bermudez, V.P., Hurwitz, J., van Oijen, A., Schärer, O.D., and Walter, J.C. (2011). Selective bypass of a lagging strand roadblock by the eukaryotic replicative DNA helicase. Cell *146*, 931–941.

Ghosal, G., Leung, J.W.-C., Nair, B.C., Fong, K.-W., and Chen, J. (2012). Proliferating cell nuclear antigen (PCNA)-binding protein C1orf124 is a regulator of translesion synthesis. J. Biol. Chem. *287*, 34225–34233.

Harley, M.E., Murina, O., Leitch, A., Higgs, M.R., Bicknell, L.S., Yigit, G., Blackford, A.N., Zlatanou, A., Mackenzie, K.J., Reddy, K., et al. (2016). TRAIP promotes DNA damage response during genome replication and is mutated in primordial dwarfism. Nat. Genet. *48*, 36–43.

Hoege, C., Pfander, B., Moldovan, G.L., Pyrowolakis, G., and Jentsch, S. (2002). RAD6-dependent DNA repair is linked to modification of PCNA by ubiquitin and SUMO. Nature *419*, 135–141.

Hoffmann, S., Smedegaard, S., Nakamura, K., Mortuza, G.B., Räschle, M., Ibañez de Opakua, A., Oka, Y., Feng, Y., Blanco, F.J., Mann, M., et al. (2016). TRAIP is a PCNA-binding ubiquitin ligase that protects genome stability after replication stress. J. Cell Biol. *212*, 63–75.

Hospenthal, M.K., Mevissen, T.E.T., and Komander, D. (2015). Deubiquitinase-based analysis of ubiquitin chain architecture using Ubiquitin Chain Restriction (UbiCRest). Nat. Protoc. *10*, 349–361.

Ide, H., Shoulkamy, M.I., Nakano, T., Miyamoto-Matsubara, M., and Salem, A.M.H. (2011). Repair and biochemical effects of DNA-protein crosslinks. Mutat. Res. *711*, 113–122.

Kim, S., Dallmann, H.G., McHenry, C.S., and Marians, K.J. (1996). Coupling of a replicative polymerase and helicase: a tau-DnaB interaction mediates rapid replication fork movement. Cell *84*, 643–650.

Kim, M.S., Machida, Y., Vashisht, A.A., Wohlschlegel, J.A., Pang, Y.-P., and Machida, Y.J. (2013). Regulation of error-prone translesion synthesis by Spartan/C1orf124. Nucleic Acids Res. *41*, 1661–1668.

Kuo, H.K., Griffith, J.D., and Kreuzer, K.N. (2007). 5-Azacytidine induced methyltransferase-DNA adducts block DNA replication in vivo. Cancer Res. *67*, 8248–8254.

Lebofsky, R., Takahashi, T., and Walter, J.C. (2009). DNA replication in nucleus-free Xenopus egg extracts. Methods Mol. Biol. *521*, 229–252.

Lessel, D., Vaz, B., Halder, S., Lockhart, P.J., Marinovic-Terzic, I., Lopez-Mosqueda, J., Philipp, M., Sim, J.C.H., Smith, K.R., Oehler, J., et al. (2014). Mutations in SPRTN cause early onset hepatocellular carcinoma, genomic instability and progeroid features. Nat. Genet. *46*, 1239–1244.

Lin, C.-P., Ban, Y., Lyu, Y.L., Desai, S.D., and Liu, L.F. (2008). A ubiquitin-proteasome pathway for the repair of topoisomerase I-DNA covalent complexes. J. Biol. Chem. *283*, 21074–21083.

Lopez-Mosqueda, J., Maddi, K., Prgomet, S., Kalayil, S., Marinovic-Terzic, I., Terzic, J., and Dikic, I. (2016). SPRTN is a mammalian DNA-binding metalloprotease that resolves DNA-protein crosslinks. eLife *5*, e21491.

Mao, Y., Desai, S.D., Ting, C.Y., Hwang, J., and Liu, L.F. (2001). 26 S proteasome-mediated degradation of topoisomerase II cleavable complexes. J. Biol. Chem. *276*, 40652–40658.

Maskey, R.S., Kim, M.S., Baker, D.J., Childs, B., Malureanu, L.A., Jeganathan, K.B., Machida, Y., van Deursen, J.M., and Machida, Y.J. (2014). Spartan deficiency causes genomic instability and progeroid phenotypes. Nat. Commun. *5*, 5744.

Maskey, R.S., Flatten, K.S., Sieben, C.J., Peterson, K.L., Baker, D.J., Nam, H.-J., Kim, M.S., Smyrk, T.C., Kojima, Y., Machida, Y., et al. (2017). Spartan deficiency causes accumulation of Topoisomerase 1 cleavage complexes and tumorigenesis. Nucleic Acids Res. *45*, 4564–4576.

McGarry, T.J., and Kirschner, M.W. (1998). Geminin, an inhibitor of DNA replication, is degraded during mitosis. Cell *93*, 1043–1053.

Mimura, S., and Takisawa, H. (1998). Xenopus Cdc45-dependent loading of DNA polymerase alpha onto chromatin under the control of S-phase Cdk. EMBO J. *17*, 5699–5707.

Mórocz, M., Zsigmond, E., Tóth, R., Enyedi, M.Z., Pintér, L., and Haracska, L. (2017). DNA-dependent protease activity of human Spartan facilitates replication of DNA-protein crosslink-containing DNA. Nucleic Acids Res. *45*, 3172–3188.

Mosbech, A., Gibbs-Seymour, I., Kagias, K., Thorslund, T., Beli, P., Povlsen, L., Nielsen, S.V., Smedegaard, S., Sedgwick, G., Lukas, C., et al. (2012). DVC1 (C1orf124) is a DNA damage-targeting p97 adaptor that promotes ubiquitin-dependent responses to replication blocks. Nat. Struct. Mol. Biol. *19*, 1084–1092.

Nakano, T., Katafuchi, A., Matsubara, M., Terato, H., Tsuboi, T., Masuda, T., Tatsumoto, T., Pack, S.P., Makino, K., Croteau, D.L., et al. (2009). Homologous recombination but not nucleotide excision repair plays a pivotal role in tolerance of DNA-protein cross-links in mammalian cells. J. Biol. Chem. *284*, 27065–27076.

Nakano, T., Ouchi, R., Kawazoe, J., Pack, S.P., Makino, K., and Ide, H. (2012). T7 RNA polymerases backed up by covalently trapped proteins catalyze highly error prone transcription. J. Biol. Chem. *287*, 6562–6572.

Novakova, O., Kasparkova, J., Malina, J., Natile, G., and Brabec, V. (2003). DNA-protein cross-linking by trans-[PtCl(2)(E-iminoether)(2)]. A concept for activation of the trans geometry in platinum antitumor complexes. Nucleic Acids Res. *31*, 6450–6460.

Ortega-Atienza, S., Green, S.E., and Zhitkovich, A. (2015). Proteasome activity is important for replication recovery, CHK1 phosphorylation and prevention of G2 arrest after low-dose formaldehyde. Toxicol. Appl. Pharmacol. *286*, 135–141.

Quiñones, J.L., Thapar, U., Yu, K., Fang, Q., Sobol, R.W., and Demple, B. (2015). Enzyme mechanism-based, oxidative DNA-protein cross-links formed with DNA polymerase β in vivo. Proc. Natl. Acad. Sci. USA *112*, 8602–8607.

Räschle, M., Knipscheer, P., Enoiu, M., Angelov, T., Sun, J., Griffith, J.D., Ellenberger, T.E., Schärer, O.D., and Walter, J.C. (2008). Mechanism of replication-coupled DNA interstrand crosslink repair. Cell *134*, 969–980.

Räschle, M., Smeenk, G., Hansen, R.K., Temu, T., Oka, Y., Hein, M.Y., Nagaraj, N., Long, D.T., Walter, J.C., Hofmann, K., et al. (2015). DNA repair. Proteomics reveals dynamic assembly of repair complexes during bypass of DNA cross-links. Science *348*, 1253671.

Ridpath, J.R., Nakamura, A., Tano, K., Luke, A.M., Sonoda, E., Arakawa, H., Buerstedde, J.M., Gillespie, D.A.F., Sale, J.E., Yamazoe, M., et al. (2007). Cells deficient in the FANC/BRCA pathway are hypersensitive to plasma levels of formaldehyde. Cancer Res. *67*, 11117–11122.

Sparks, J.L., Chistol, G., Gao, A.O., Räschle, M., Larsen, N.B., Mann, M., Duxin, J.P., and Walter, J.C. (2018). The CMG helicase bypasses DNA-protein cross-Links to facilitate their repair. Cell *176*, https://doi.org/10.1016/j.cell.2018.10.053.

Stingele, J., Schwarz, M.S., Bloemeke, N., Wolf, P.G., and Jentsch, S. (2014). A DNA-dependent protease involved in DNA-protein crosslink repair. Cell *158*, 327–338.

Stingele, J., Habermann, B., and Jentsch, S. (2015). DNA-protein crosslink repair: proteases as DNA repair enzymes. Trends Biochem. Sci. *40*, 67–71.

Stingele, J., Bellelli, R., Alte, F., Hewitt, G., Sarek, G., Maslen, S.L., Tsutakawa, S.E., Borg, A., Kjær, S., Tainer, J.A., et al. (2016). Mechanism and Regulation of DNA-Protein Crosslink Repair by the DNA-Dependent Metalloprotease SPRTN. Mol. Cell *64*, 688–703.

Tada, S., Li, A., Maiorano, D., Méchali, M., and Blow, J.J. (2001). Repression of origin assembly in metaphase depends on inhibition of RLF-B/Cdt1 by geminin. Nat. Cell Biol. *3*, 107–113.

Tretyakova, N.Y., Groehler, A., 4th, and Ji, S. (2015). DNA-Protein Cross-Links: Formation, Structural Identities, and Biological Outcomes. Acc. Chem. Res. *48*, 1631–1644.

Tyanova, S., Temu, T., Sinitcyn, P., Carlson, A., Hein, M.Y., Geiger, T., Mann, M., and Cox, J. (2016). The Perseus computational platform for comprehensive analysis of (prote)omics data. Nat. Methods *13*, 731–740.

Vaz, B., Popovic, M., Newman, J.A., Fielden, J., Aitkenhead, H., Halder, S., Singh, A.N., Vendrell, I., Fischer, R., Torrecilla, I., et al. (2016). Metalloprotease SPRTN/DVC1 Orchestrates Replication-Coupled DNA-Protein Crosslink Repair. Mol. Cell *64*, 704–719.

Vaz, B., Popovic, M., and Ramadan, K. (2017). DNA-Protein Crosslink Proteolysis Repair. Trends Biochem. Sci. *42*, 483–495.

Walter, J., Sun, L., and Newport, J. (1998). Regulated chromosomal DNA replication in the absence of a nucleus. Mol. Cell *1*, 519–529.

Walter, T.S., Meier, C., Assenberg, R., Au, K.-F., Ren, J., Verma, A., Nettleship, J.E., Owens, R.J., Stuart, D.I., and Grimes, J.M. (2006). Lysine methylation as a routine rescue strategy for protein crystallization. Structure *14*, 1617–1622.

Wohlschlegel, J.A., Dwyer, B.T., Dhar, S.K., Cvetic, C., Walter, J.C., and Dutta, A. (2000). Inhibition of eukaryotic DNA replication by geminin binding to Cdt1. Science *290*, 2309–2312.

Zecevic, A., Hagan, E., Reynolds, M., Poage, G., Johnston, T., and Zhitkovich, A. (2010). XPA impacts formation but not proteasome-sensitive repair of DNA-protein cross-links induced by chromate. Mutagenesis *25*, 381–388.

STAR★METHODS

KEY RESOURCES TABLE

REAGENT or RESOURCE	SOURCE	IDENTIFIER
Antibodies		
Rabbit polyclonal anti-Rev1-N	Budzowska et al., 2015	N/A
Rabbit polyclonal anti-Rev1-C	Budzowska et al., 2015	N/A
Rabbit polyclonal anti-Orc2	Fang and Newport, 1993	N/A
Rabbit polyclonal anti-Cdc45	Mimura and Takisawa, 1998	N/A
Rtel1	Sparks et al., 2018	N/A
Sld5	Dewar et al., 2017	N/A
Rabbit polyclonal anti-Psma1	New England Peptides	3514
Rabbit polyclonal anti-Psma3	New England Peptides	3516
Rabbit polyclonal anti-Sprtn	New England Peptides	3703
Rabbit polyclonal anti-Traip	New England Peptides	3472
Rabbit polyclonal anti-MCM6	New England Peptides	2926
Rabbit polyclonal anti-Sprtn-N	Pocono Rabbit Farm and Laboratory	Rabbit # 31053
Rabbit polyclonal anti-M.HpaII	Pocono Rabbit Farm and Laboratory	Rabbit # 31495 and 31496
Rabbit polyclonal anti-H3	Cell Signaling	Cat# 9715S; RRID:AB_331563
Bacterial and Virus Strains		
E. coli T7 express	New England Biolabs	C2566H
E. coli Rosetta 2 (DE3) pLysS	Novagen	71-401-3
Chemicals, Peptides, and Recombinant Proteins		
Geminin	McGarry and Kirschner, 1998	N/A
LacI-biotin	Duxin et al., 2014	N/A
M.HpaII	Duxin et al., 2014	N/A
*xl*SPRTN-WT	This study	N/A
*xl*SPRTN-EQ	This study	N/A
*xl*SPRTN-SHP^{x}	This study	N/A
*xl*SPRTN-PIP^{x}	This study	N/A
*xl*SPRTN-UBZ^{x}	This study	N/A
*xl*SPRTN-1-435	This study	N/A
*xl*TRAIP-WT	This study	N/A
*xl*TRAIP-R18C	This study	N/A
UbVS	Boston Biochem	U-202
Human Recombinant Ubiquitin	Boston Biochem	U-100H
Human Recombinant FLAG-Ubiquitin	Boston Biochem	U-120
dnUBC9	Azuma et al., 2003	N/A
Human chorionic ganotropin	Sigma	CG10-10VL
MG262	Boston Biochem	I-120
Alpha-32P-deoxyadenosinetriphosphate	Perkin Elmer	BLU512H250UC
Gamma-32P-adenosinetriphosphate	Perkin Elmer	BLU502A100UC
Proteinase K, recombinant	Roche	3115879001
Aphidicolin	Sigma	A0781-1MG
Ara-cytidine-5′triphosphate	Jena Bioscience	NU-1170S
Exonuclease I	New England Bioscience	M0293S
Protein A Sepharose Fast Flow	GE Health Care	17-1279-01

(*Continued on next page*)

Continued

REAGENT or RESOURCE	SOURCE	IDENTIFIER
RNase A	Thermo Fisher	EN0531
Gel Loading Dye II	Invitrogen	AM8547
EDTA-free Complete protease inhibitor cocktail	Roche	11873580001
Anti-FLAG M2 affinity resin	Sigma	A2220-5ML
3xFLAG peptide	Sigma	F4766-4MG
Streaptavidin-coupled magnetic beads M-280	Invitrogen	11205D
Benzonase	Novagen	70746-3
FLAG M2 magnetic beads	Sigma	M8823-1ML
His-tag Dynabeads	Thermo Fisher Scientific	10103D
LysC	Life Technologies	90051
Trypsin	Life Technologies	90305
AluI Methyltransferase	New England BioLabs	M0220S
Critical Commercial Assays		
UbiCREST	Boston Biochem	K-400
Reductive Alkylation Kit	Hampton Research	HR2-434
Quickchange II mutagenesis kit	Aglient	200523
Thermo Sequenase Cycle Sequencing kit	USB	785001KT
Bac to Bac Expression System	Thermo Fisher Scientific	10359016
Deposited Data		
ProteomeXchange	This study	PXD008831
Experimental Models: Cell Lines		
Sf9 Insect cells	Thermo Fosher Scientific	B82501
Experimental Models: Organisms/Strains		
Xenopus laevis (females)	Nasco	LM0053MX
Xenopus laevis (males)	Nasco	LM00715MX
Oligonucleotides		
JLS2: GGGAGCTGAATGCCGCGCGAATAATGGTTTCTTAGACGT	This study	N/A
JLS3: CATCCACTAGCCAATTTATGCTGAGGTACCGGATTGAGTA GCTACCGGATGCTGAGGGGAT CCACTAGCCAATTTATCATGG	This study	N/A
NBL104: AATTCCTCAGCATCCGGTTCGAACTCAATAGCTTACCT CAGCCA	This study	N/A
Gap_capture: GGTACCGGATTGAGTAGCTACCGGATGCTGA	This study	Tag Copenhagen
dFdC_Lead: TCAGCATCCGGTAGCTACTCAATC[C5-Fluro dC]GGTACC	This study	BioSynthesis
AluI-HpaII_dFdC: TCAGCATC[C5-FlurodC]GGTTCGAACTCAATAG[C5-FlurodC]TTACC-3	This study	BioSynthesis
dFdC_Lag: TGAGGTAC[C5-FlurodC]GGATTGAGTAGCTACCGGATGC	This study	BioSynthesis
Reference ladder: 5′-CATTCAGCTCCCGGAGACGGTCACAGCTTG TCTGTAAGCGGATGCCGGGAGCAGACAAGCCCGTCAGGGCGCGT CAGCGGGTGTGGCGGG TGTCGGGGCTGGCTTAACTATGCGGCA TCAGAGCAGATTGTACTGAGAGTGCACCATATGGC TGAGGTACCG	This study	Tag Copenhagen
Sequencing ladder: CATTCAGCTCCCGGAGACGGTC	This study	Tag Copenhagen
Primer A: 5′ – GAT CGG ATC CAT GGA CTA CAA AGA CGA TGA CGA CAA GGG TGA TAT GCA GAT GTC GGT AG – 3′	This study	IDT
Primer B: 5′- GAT CCT CGA GTT ATT ATG TAT TGC AGT TTT GTA AGC AGG TGT CTA AAT G –3′	This study	IDT
Recombinant DNA		
pJLS2	This study	N/A
pJLS3	This study	N/A
pNBL104	This study	N/A

(*Continued on next page*)

Continued		
REAGENT or RESOURCE	SOURCE	IDENTIFIER
pFastBac1-*xl*SPRTN-WT	This study	N/A
pFastBac1- *xl*SPRTN-EQ	This study	N/A
pFastBac1-*xl*SPRTN-SHP^x	This study	N/A
pFastBac1-*xl*SPRTN-PIP^x	This study	N/A
pFastBac1-*xl*SPRTN-UBZ^x	This study	N/A
pFastBac1-*xl*SPRTN-1-435	This study	N/A
pH6-SUMO-*xl*TRAIP-WT	This study	N/A
pH6-SUMO-*xl*TRAIP-R18C	This study	N/A
Software and Algorithms		
ImageJ 1.51	NIH	https://imagej.nih.gov/ij
MaxQuant	Cox and Mann, 2008	http://www.coxdocs.org
Perseus 1.5.6.0	Tyanova et al., 2016	http://www.coxdocs.org

CONTACT FOR REAGENT AND RESOURCE SHARING

Further information and requests for resources and reagents should be directed to and will be fulfilled by the Lead Contact, Julien P. Duxin (julien.duxin@cpr.ku.dk).

EXPERIMENTAL MODEL AND SUBJECT DETAILS

Egg extracts were prepared using *Xenopus laevis* (Nasco Cat #LM0053MX, LM00715MX). All experiments involving animals were approved by the Harvard Medical Area Institutional Animal Care and Used Committee and by the Danish Animal Experiments Inspectorate, and are conform to relevant regulatory standards and European guidelines.

METHOD DETAILS

Xenopus Egg Extracts and DNA Replication

Preparation of *Xenopus* egg extracts was performed as described previously (Lebofsky et al., 2009; Walter et al., 1998). For high-speed supernatant (HSS) preparation, 6 female frogs (Nasco) were primed by injection with 80 IU of human chorionic gonadotropin (hCG, Sigma). 2-7 days after priming, frogs were injected with 625 IU of hCG and placed in individual tanks containing 100 mM NaCl. 18-20 hours post injection, eggs were collected and used for extract preparation. Eggs were first dejellied in cysteine buffer for 7 min (2.2% cysteine-HCl, pH 7.7), washed 3 times in in 0.5X MMR buffer (final concentration: 50 mM NaCl, 1 mM KCl, 0.25 mM $MgSO_4$, 1.25 mM $CaCl_2$, 2.5 mM HEPES, 0.05 mM EDTA, pH 7.8) and washed 3 times in ELB sucrose buffer (2.5 mM $MgCl_2$, 50 mM KCl, 10 mM HEPES, 250 mM sucrose, 1 mM DTT, 50 μg/mL cyclohexamide, pH 7.8). Eggs were packed for 1 min at 176 x g for 1 min and crushed for 20 min at 20, 000 x g in a swing bucket rotor at 4°C in the presence of cytochalasin B (final concentration: 2.5 μg/mL), aprotinin (final concentration: 5 μg/mL) and leupeptin (final concentration: 5 μg/mL). Crude interphase extract was recovered post-centrifugation and spun in ultracentrifuge for 90 min at 260,000 x g at 2°C following addition of cyclohexamide (final concentration: 50 μg/mL), DTT (final concentration: 1 mM), aprotinin (final concentration: 10 μg/mL), leupeptin (final concentration: 10 μg/mL) and cytochalasin B (final concentration: 5 μg/mL). Following centrifugation, the small lipid layer on top was removed. The soluble HSS was harvested, snap frozen in 33 uL aliquots and stored at −80°C. For nucleoplasmic egg extract (NPE), 20 female frogs were injected and the crude interphase extract was prepared in the same manner than for HSS. Once collected the crude interphase extract was supplemented with cyclohexamide (final concentration: 50 μg/mL), DTT (final concentration: 1 mM), aprotinin (final concentration: 10 μg/mL), leupeptin (final concentration: 10 μg/mL), cytochalasin B (final concentration: 5 μg/mL) and nocadazole (final concentration: 3.3 ug/mL). The extract was spun at 20 000 x g at 4°C for 10 min. The lipid layer on top was removed and the interphase extract decanted to a new tube. The interphase extract was supplemented with ATP (final concentration: 2 mM), phosphocreatine (final concentration: 20 mM) and creatine phosphokinase (final concentration: 5 μg/mL) and nuclear assembly reactions were initiated by adding demembranated sperm chromatin to a final concentration of 4,400/μL. The nuclear assembly reaction was incubated at room temperature for 60-85 min, and then spun for 2 min at 20 000 x g in a swing-bucket rotor. The nuclear layer on top was recovered and then spun in a swinging bucket rotor at 260,000 x g at 2°C for 30 min. Lipids on top were removed and the clear soluble NPE was harvested. 10 μL NPE aliquots were snap-frozen and kept at −80°C.

For DNA replication, plasmids were first incubated in HSS (final concentration: 7.5 ng DNA/μL HSS), supplemented with nocadazole (final concentration: 3 μg/mL) and ATP regeneration mix (final concentration: 20 mM phosphocreatine, 2 mM ATP, 5 μg/mL creatine phosphokinase, for 20-30 min at room temperature to license the DNA. Two volumes of NPE supplemented with 4 mM DTT,

20 mM phosphocreatine, 2 mM ATP, 5 μg/mL creatine phosphokinase were then added to 1 volume of licensing reaction to initiate replication. Where indicated, HSS was supplemented with Geminin at a final concentration of 10 μM and incubated for 10 min at room temperature prior to addition of plasmid DNA. For replication in the presence of LacI, plasmid DNA (75 ng/uL) was incubated with an equal volume of 12 μM LacI for 1 hr prior to HSS addition (Duxin et al., 2014). For UbVS treatment, NPE was supplemented with 22.5 μM ubiquitin vinyl sulfone (UbVS) (Boston Biochem) and incubated for 15 min prior to mixing with HSS (15 μM final concentration). Where indicated, recombinant ubiquitin or FLAG-ubiquitin (Boston Biochem) were added to NPE at a concentration of 120 μM (80 μM final concentration). For SPRTN depletion-rescue experiments, NPE was supplemented with 30 nM recombinant wild-type or mutant *Xenopus* SPRTN. For TRAIP depletion-rescue experiments, NPE was supplemented with 100 nM of recombinant wild-type or R18C *Xenopus* TRAIP. To block *de novo* SUMOylation, dnUBC9 was added to extracts to a final concentration of 10 μM (Azuma et al., 2003). Where indicated, proteasome activity was inhibited via the addition of 200 μM MG262 (Boston Biochem) to extracts (final concentration). For DNA labeling, reactions were supplemented with [α-^{32}P]dATP. To analyze plasmid replication intermediates, 1 μL of each reaction was added to 5 μL of replication stop solution A (5% SDS, 80 mM Tris pH 8.0, 0.13% phosphoric acid, 10% Ficoll) supplemented with 1 μL of Proteinase K (20 mg/ml) (Roche). Samples were incubated for 1 hr at 37°C prior to separation by 0.9% native agarose gel electrophoresis and visualization using a phosphorimager (Lebofsky et al., 2009). For analysis of nascent leading strand products, 3-4 μL of each replication reaction was added to 10 volumes of 50 mM Tris pH 7.5, 0.5% SDS, 25 mM EDTA, and replication intermediates were purified by phenol chloroform extraction. For incubation in non-licensing extracts, one volume of HSS and two volumes of NPE were premixed prior to the addition of plasmid DNA (final concentration of 10 ng/μL). Where indicated, aphidicolin (Sigma) and *ara*-cytidine-5′-triphosphate (araCTP) (Jena Bioscience), were added to a final concentration of 700 μM and 1 mM, respectively. All experiments were performed at least in duplicate and a representative experiment is shown. Radioactive signal was quantified using ImageJ (NIH, USA).

Preparation of DNA constructs

To generate pDPC we first created pJLS2 by replacing the AatII-BsmBI fragment from pJD2 (Duxin et al., 2014) with the following sequence:

5′-GGGAGCTGAATGCCGCGCGAATAATGGTTTCTTAGACGT-3′ which contains a Nb.BsmI site.

To generate pDPC^{2xLead}, the SacI-BssHII fragment from pJLS2 was replaced with the following sequence:

5′CATCCACTAGCCAATTTATGCTGAGGTACCGGATTGAGTAGCTACCGGATGCTGAGGGGATCCACTAGCCAATTTATCATGG-3′.

pJLS2 or pJLS3 were nicked with Nt.BbvcI and ligated with the following oligo containing a fluorinated cytosine: 5′-TCAG CATCCGGTAGCTACTCAATC[C5-Fluro dC]GGTACC-3′ and subsequently crosslinked to M.HpaII-His$_6$ to generate pDPC or pDPC^{2xLead}, respectively, as previously described (Duxin et al., 2014). To this end, the modified fluorinated DNA was gel purified and mixed with M.HpaII-His$_6$ in reaction buffer (50 mM Tris-HCl pH 7.5, 5 mM 2-mercaptoethanol, 10 mM EDTA) supplemented with 100 μM of S-adenosylmethionine (NEB) for 12 hr at 37°C. To generate pDPC^{PK}, pDPC was treated with Proteinase K (37°C overnight in presence of 0.5% SDS) to reduce the DPC to a 4 amino acids peptide adduct. The plasmid was subsequently recovered by phenol/chloroform extraction. To generate pDPC$^{+peptide}$ the ApoI-NdeI fragment of pJLS2 was replaced with the following sequence: 5′- AATTCCTCAGCATCCGGTTCGAACTCAATAGCTTACCTCAGCCA-3′, generating pNBL104. pNBL104 was nicked with Nt.BbvCI and ligated with the following oligo containing both a fluorinated AluI site and a fluorinated M.HpaII site: 5′- TCAGCATC[C5-FlurodC] GGTTCGAACTCAATAG[C5-FlurodC]TTACC-3′. AluI Methyltransferase (New England BioLabs) was first crosslinked to the plasmid, degraded with Proteinase K (37°C overnight in presence of 0.5% SDS) and the plasmid was recovered by phenol/chloroform extraction. The peptide-containing plasmid was then crosslinked to M.HpaII-His$_6$ as described above. To generate pDPC^{ssDNA}, pJLS2 was nicked with Nb.BbvCI and ligated with the following fluorinated oligo: 5′-TGAGGTAC[C5-FlurodC]GGATTGAGTAGCTACCG GATGC-3′. The dFdC-containing plasmid was cut with Nt.BbvCI and the resulting 31bp fragment was melted off and captured by annealing to an excess complimentary oligo 5′-GGTACCGGATTGAGTAGCTACCGGATGCTGA-3′. Excess oligos were then degraded by Exonuclease I (New England BioLabs) treatment. The gapped plasmid was then recovered by phenol/chloroform extraction and crosslinked to M.HpaII-His$_6$ as described above.

Antibodies and Immunodepletion

The following antibodies used were described previously: REV1 (Budzowska et al., 2015), ORC2 (Fang and Newport, 1993). M.HpaII antibody was raised against full length M.HpaII-His$_6$ expressed and purified from bacteria under denaturing conditions (Pocono Rabbit Farm & Laboratory). PSMA1, PSMA3, SPRTN, TRAIP and MCM6 antibodies were raised by New England Peptide by immunizing rabbits with Ac-CAEEPVEKQEEPMEH-OH, Ac-CKYAKESLEEEDDSDDDNM-OH, Aoa-DVLQDKINDHLDTCLQNCNT-OH, Ac-CTSSLANQPRLEDFLK-OH and Ac-CLVVNPNYMLED-OH, respectively. SPRTN-N antibody was raised against a fragment of *Xenopus laevis* SPRTN encompassing amino acids 67-287 which was tagged on N terminus with His$_6$. The protein fragment was purified from bacteria under denaturing conditions and the antibody was raised by Pocono Rabbit Farm & Laboratory. Western blotting analysis for H3 was carried out with commercial antibody from Cell Signaling (Cat #9715S).

To immunodeplete SPRTN from *Xenopus* egg extracts, one volume of Protein A Sepharose Fast Flow (PAS) (GE Health Care) was mixed with 4 volumes of affinity purified SPRTN peptide antibody (1 mg/mL) and incubated overnight at 4°C. The beads were then washed twice with 500 μL PBS, once with ELB (10 mM HEPES pH 7.7, 50 mM KCl, 2.5 mM $MgCl_2$, and 250 mM sucrose), three times with ELB supplemented with 0.5 M NaCl, and twice with ELB. One volume of precleared HSS or NPE was then depleted by mixing with 0.2 volumes of antibody-bound beads then incubating at room temperature for 20 min. The depletion procedure was repeated once. To immunodeplete PSMA1, one volume of PAS beads was mixed with 10 volumes of affinity purified PSMA1 peptide antibody (1 mg/mL). The beads were washed as described above, and one volume of precleared HSS or NPE was then depleted by mixing with 0.2 volumes of antibody-bound beads and then incubating at room temperature for 20 min. The depletion procedure was repeated three times for HSS and twice for NPE. For SPRTN and PSMA1 combined depletion, one volume of PAS beads was mixed with 4 volumes of affinity purified SPRTN peptide antibody and 10 volumes of affinity purified PSMA1 peptide antibody. The beads were washed and depletion was performed as described for PSMA1 immunodepletion. The immunodepletion of REV1 was performed as previously described (Budzowska et al., 2015). To immunodeplete TRAIP, one volume of Protein A Sepharose Fast Flow (PAS) (GE Health Care) was mixed with 2.5 volumes of affinity purified TRAIP antibody (1 mg/mL) and incubated overnight at 4°C. The beads were washed as described above, and one volume of precleared HSS or NPE was then depleted by mixing with 0.2 volumes of antibody-bound beads and then incubating at room temperature for 20 min. The depletion procedure was repeated twice for HSS and twice for NPE. Experiments in Figures 7 and S7E–S7G were performed using the SPRTN-N antibody where one volume of PAS was mixed with 3 volumes of SPRTN-N serum and incubated overnight at 4°C. The beads were washed as described above, and one volume of precleared HSS or NPE was then depleted by mixing with 0.2 volumes of antibody-bound beads and then incubating at room temperature for 20 min. The depletion procedure was repeated once.

Nascent-Strand Analysis

Nascent strand analysis was performed as previously described (Räschle et al., 2008). Briefly, purified DNA was digested with the indicated restriction enzymes followed by addition of 0.5 volumes of Gel Loading Dye II (Denaturing PAGE) (Life Technologies). DNA fragments were subsequently separated on 5% or 7% denaturing polyacrylamide gels, transferred to filter paper, dried, and visualized using a phosphorimager. Radioactive signal was quantified using ImageJ (NIH, USA).

Reference oligo used in Figure S4I: 5′-CATTCAGCTCCCGGAGACGGTCACAGCTTG TCTGTAAGCGGATGCCGGGAGCAGA CAAGCCCGTCAGGGCGCGTCAGCGGGTGTGGCGGGTGTCGGGGCTGGCTTAACTATGCGGCATCAGAGCAGATTGTACTGAGA GTGCACCATATGGCTGAGGTACCG-3′.

Primer used for dideoxy-sequencing ladder in Figure S4I: 5′- CAT TCA GCT CCC GGA GAC GGT C – 3′.

Protein Expression and Purification

M.HpaII-His_6 was expressed and purified as previously described (Duxin et al., 2014). Briefly, pHpaII-Avitag-His_6 was transformed in T7 Express Competent E.coli cells (NEB), cells cultured in the presence of 100 μg/mL ampicillin until the OD_{600} reached 0.7. The culture was supplemented with 0.5 mM IPTG for 3 hours, collected by centrifugation and resuspended in 15 mL Lysis Buffer (20 mM Tris pH 8.5, 500 mM KCl, 10% glycerol, 10 mM imidazole and protease inhibitors (Roche)). Cells were lysed by sonication and cleared by centrifugation at 20, 000 x g for 30 min. Cleared lysate was applied onto Ni-NTA resin (QAGEN). The resin was washed with 25 mL of Lysis Buffer containing 30 mM imidazole and the protein eluted with Elution Buffer (20 mM Tris pH 8.5, 100 mM KCl, 10% glycerol and 250 mM imidazole). Eluate was dialyzed overnight in Storage Buffer (20 mM Tris pH 8.5, 100 mM KCl, 1 mM DTT, 30% glycerol) and protein aliquots snap frozen and kept at −80°C. To generate lysine-methylated M.HpaII, purified M.HpaII-His_6 was first denatured by dialyzing against 20 mM HEPES pH 7.5, 100 mM KCl, 6M Guanidine HCl, 10% glycerol. Denatured M.HpaII protein was then methylated using Reductive Alkylation Kit (Hampton Research) via the addition of dimethylamine borane and formaldehyde according to the manufacturer's protocols. The methylation reaction was stopped by addition of 100 mM Tris pH 7.5 and 5 mM DTT (final concentrations). Methylated M.HpaII was then renatured by sequentially dialyzing against Renaturing Buffer (20 mM Tris pH 8.5, 100mM KCl, 1mM DTT, 10% glycerol) supplemented with 4, 2, and 0 M Guanidine HCl for 1 hr each at 4°C. The renatured protein was then dialyzed against storage buffer (20 mM Tris pH 8.5, 100 mM KCl, 1 mM DTT, 30% glycerol) and stored at −80°C.

LacI-biotin protein was purified from T7 Express Competent cells (NEP) (Duxin et al., 2014). Briefly, pET11a-LacI and pBirAcm (Avidity) were co-transformed and cells cultured in the presence of 100 μg/mL ampicillin and 34 μg/mL chloramphenicol at 37°C until OD_{600} reached 0.6. The culture was supplemented with 1 mM IPTG and 50 μM biotin for 2 hours. Cells were collected by centrifugation and resuspended in Buffer 1 (50 mM Tris pH 7.5, 5 mM EDTA, 100 mM NaCl, 10% sucrose, 1 mM DTT, protease inhibitors (Roche), 0.2 mg/mL lysozyme (Sigma), 0.1% Brij 58) and rotated for 30 min at room temperature. The cell lysate was pelleted by centrifugation for 60 min at 20, 000 x g and the insoluble pellet was resuspended in 10 mL of Extraction Buffer (50 mM Tris pH 7.5, 5 mM EDTA, 1M NaCl, 30 mM IPTG, 1 mM DTT and protease inhibitors). The resuspended pellet was homogenized by sonication and pelleted again for 60 min at 20, 000 x g. The supernatant was collected and 1% polymin P was added to 0.045%. Lysate was rotated for 30 min at 4°C and pelleted at 20, 000 x g for 20 min. The supernatant was transferred to a new tube and ammonium sulfate was added to a final saturation of 37% followed by rotation for 30 min at 4°C. The pellet was recovered and resuspended in 2 mL of Wash Buffer (50 mM Tris pH 7.5, 1 mM EDTA, 100 mM NaCl, 1 mM DTT and protease inhibitors). The resuspension was applied to a column containin 1 mL of softlink avidin resin and inbutated for 1 hour at 4°C. The column was washed with 15 mL of Wash Buffer, and the protein eluted with Elution buffer (50 mM Tris pH 7.5, 1 mM EDTA, 100 mM NaCl, 1 mM DTT and 5 mM biotin). Protein was

dialyzed overnight with Dialysis Buffer (50 mM Tris pH 7.5, 1 mM EDTA, 150 mM NaCl, 1 mM DTT and 30% glycerol) and stored at −80°C.

Xenopus SPRTN with an N-terminal FLAG tag was cloned into pFastBac1 (Thermo Fisher Scientific) using primers A and B. SPRTN mutations were introduced via Quikchange mutagenesis and confirmed by Sanger sequencing. SPRTN Baculoviruses were prepared using the Bac-to-Bac system (Thermo Fisher Scientific) according to the manufacturer's protocols. SPRTN was expressed in 250 mL suspension cultures of Sf9 insect cells (Thermo Fisher Scientific) by infection with SPRTN baculovirus for 48 hr. Sf9 cells were subsequently collected via centrifugation and resuspended in Lysis Buffer (50 mM Tris pH 7.5, 500 mM NaCl, 10% Glycerol, 1X Roche EDTA-free Complete protease inhibitor cocktail, 0.5 mM PMSF, 0.2% Triton X-100). To lyse cells, the suspension was subjected to three freeze/thaw cycles, passed through a 21 g needle, and then sonicated. The cell lysate was spun at 25000 rpm in a Beckman SW41 rotor for 1hr. The soluble fraction was collected and then incubated with 200 μL anti-FLAG M2 affinity resin (Sigma) for 90 min at 4°C. The resin was then washed once with 10 mL Lysis Buffer, twice with Wash Buffer (50 mM Tris pH 7.5, 500 mM NaCl, 10% Glycerol, 0.2% Triton X-100), and three times with Buffer A (50 mM Tris pH 7.5, 500 mM NaCl, 10% Glycerol). FLAG-SPRTN was eluted with Buffer A supplemented with 100 μg/mL 3xFLAG peptide (Sigma). Elution fractions containing FLAG-SPRTN protein were pooled and dialyzed against 20 mM Tris pH 7.5, 300 mM NaCl, 10% Glycerol, 1mM DTT at 4°C for 12 hr and then dialyzed against Storage Buffer (20 mM Tris pH 7.5, 150 mM NaCl, 10% Glycerol, 1mM DTT) at 4°C for 3 hr. Aliquots of FLAG-SPRTN were then stored at −80°C.

Xenopus recombinant TRAIP wild-type (WT) and TRAIP R18C were expressed and purified with a 6xHis-SUMO tag in bacteria. Briefly, Rosetta 2 (DE3) pLysS competent cells (Novagen) were transformed with pH_6-SUMO-TRAIP WT or pH_6-SUMO TRAIP R18C and cells grown in the presence of 100 μg/mL ampicillin and 27 μg/mL chloramphenicol at 37°C until OD_{600} reached 0.6. Cells were then transferred to 16°C for 30 min and supplemented with 0.1 mM IPTG and 50 μM $ZnSO_4$ overnight. The culture was collected by centrifugation and resuspended in Lysis Buffer (20 mM HEPES pH 7.5, 400 mM sodium acetate, 10% glycerol, 20 mM imidazole, 10 μM $ZnSO_4$, 0.1% NP-40, 1 mM DTT and protease inhibitors). The lysate was sonicated, and ammonium sulfate and polyethyleneimine were added to final concentrations of 300 mM and 0.45%, respectively, and incubated for 15 min at 4°C. The lysate was centrifuged at 40, 000 x g for 45 min and the soluble fraction recovered and precipitated with ammonium sulfate. The precipitated fraction was collected by centrifugation at 40,000 x g for 45 min and resuspended in Lysis Buffer and rotated for 30 min with NiNTA resin at room temperature. The resin was washed three times with Wash Buffer (20 mM HEPES pH 7.5, 400 mM sodium acetate, 10% glycerol, 20 mM imidazole, 10 μM $ZnSO_4$, 0.01% NP-40, 1 mM DTT and protease inhibitors) and the protein was eluted from resin with Elution Buffer (20 mM HEPES pH 7.5, 400 mM sodium acetate, 10% glycerol, 120 mM imidazole, 10 uM $ZnSO_4$, 0.01% NP-40, 1 mM DTT). The eluate was then dialized with Dialysis Buffer (20 mM HEPES pH 7.5, 400 mM sodium acetate, 120 mM imidazole, 10% glycerol) overnight at 4°C in the presence of 0.03 mg/mL Ulp1. Aliquots were flash frozen and stored at −80°C.

Xenopus recombinant 6xHis-Geminin was expressed and purified as previously described (McGarry and Kirschner, 1998). Briefly, BL21 cells were transformed with pET28a-His-Geminin and cultured until the OD_{600} reached 0.6. The culture was supplemented with 0.5 mM IPTG for 3 hours, collected by centrifugation and resuspended in 10 mL Buffer S (50 mM NaPi pH 7.6, 5 mM BME, 1 mM PMSF and 2 mM benzamidine) containing 10 mg lysozyme and 1% Triton X-100. Cells were incubated for 10 min at room temperature and supplemented with 1 mL of 5M NaCl. Cells were then sonicated and pelleted at at 20, 000 x g for 30 min. Cleared lysate was supplemented with 20 mM imidazole and applied onto Ni-NTA resin (QAGEN) for 1 hour at 4C. The resin was washed with Buffer W (50 mM NaPi pH 7.6, 0.5 M NaCl, 0.1% Triton X-100, 5 mM BME, 20 mM imidazole), and the protein eluted with Elution Buffer (50 mM NaPi pH 7.6, 0.5M NaCl, 5 mM BME). The protein was dialyzed overnight with (10 mM Tris pH 8, 0.5M NaCl and 5% glycerol). Aliquots were flash frozen and stored at −80°C.

Primer A: 5′ – GAT CGG ATC CAT GGA CTA CAA AGA CGA TGA CGA CAA GGG TGA TAT GCA GAT GTC GGT AG – 3′

Primer B: 5′- GAT CCT CGA GTT ATT ATG TAT TGC AGT TTT GTA AGC AGG TGT CTA AAT G −3′

Plasmid Pull-Downs

Plasmid pull-down assays were performed as previously described (Budzowska et al., 2015). 6 μL/pull down of streptavidin-coupled beads (Dynabeads M-280, Invitrogen) were washed three times with wash buffer 1 (50 mM Tris pH7.5, 150 mM NaCl, 1 mM EDTA pH 8, 0.02% Tween-20). Biotinylated LacI was added to the beads at 12 pmol/6 μL of beads, and incubated at RT for 1 hour. The beads were washed four times with pull-down buffer (10 mM HEPES pH 7.7, 50 mM KCl, 2.5 mM $MgCl_2$, 250 mM sucrose, 0.25 mg/mL BSA, 0.02% Tween-20) and resuspended in 40 μL of the same buffer and stored on ice. At the indicated time point, 6 to 8 μL of reaction samples were withdrawn and gently mixed with the beads. The suspension was rotated for 30 min at 4°C. The beads were then washed 2 times with wash buffer 2 (10 mM HEPES pH 7.7, 50 mM KCl, 2.5 mM MgCl2, 0.25 mg/mL BSA and 0.03% Tween-20). After washing, beads were resuspended in 40 μL of 2x Laemmli sample buffer and equal volume of protein samples were resolved on SDS-PAGE gels. Proteins associated with the chromatin fraction were visualized by western blotting with the indicated antibodies and developed using the chemiluminescence function on Amersham Imager 600 (GE Healthcare).

DPC Pull-Downs

We developed a modified plasmid pull-down protocol to specifically isolate M.HpaII DPCs from extracts (Figure 1A). Streptavidin-coupled magnetic beads (Dynabeads M-280, Invitrogen; 5 μL per pull-down) were washed twice with 50 mM Tris pH 7.5, 150mM NaCl, 1mM EDTA pH 8, 0.02% Tween-20. Biotinylated LacI was added to the beads (1 pmol per 5 μL of beads) and incubated

at room temperature for 40 min. The beads were then washed four times with DPC pull-down buffer (20 mM Tris pH 7.5, 150 mM NaCl, 2 mM EDTA pH 8, 0.5% IPEGAL-CA630) and then stored in the same buffer on ice until needed. At the indicated times during DNA replication or gap filling, equal volumes (2-10 μL) of reaction were withdrawn and stopped in 300 μL of DPC pull-down buffer on ice. After all of the time points were taken, 5 μL of LacI-coated streptavidin Dynabeads were added to each sample and allowed to bind for 30-60 min at 4°C rotating. 20 μL of pull-down supernatant was mixed with 20 μL of 2X Laemmli sample buffer for input. The beads were subsequently washed four times with DPC pull-down buffer and then twice with Benzonase buffer (20 mM Tris pH 7.5, 150 mM NaCl, 2 mM $MgCl_2$, 0.02% Tween-20) before being resuspended in 15 μL Benzonase buffer containing 1 μL Benzonase (Novagen). Samples were incubated for 1hr at 37°C to allow for DNA digestion and DPC elution, after which the beads were pelleted and the supernatant M.HpaII eluate was mixed with 2X Laemmli sample buffer for subsequent western blotting analysis.

For FLAG immunoprecipitation analysis of isolated DPCs (Figure 1D), the M.HpaII eluate resulting from Benzonase treatment was instead diluted to 300 μL in Benzonase buffer. FLAG M2 magnetic beads (Invitrogen; 5 μL per pull-down) were added to each sample and allowed to bind for 60 min at 4°C rotating. The beads were subsequently washed four times with Benzonase buffer. To elute precipitated proteins, the beads were then resuspended in 0.1 M Glycine pH 3 and incubated with gentle shaking for 10 min at room temperature. After pelleting the beads, the supernatant was neutralized with 10mM Tris pH 11 and mixed with 2X Laemmli buffer.

For UbiCREST analysis of isolated DPCs (Figures S1E and S1F), pull down samples were washed with DPC pull-down buffer as described initially but were instead then washed and resuspended in 1X DUB reaction buffer and treated with the indicated deubiquitinase(s) (Boston Biochem; Hospenthal et al., 2015) at 37°C for 30 min. The samples were subsequently washed twice with Benzonase buffer and eluted with Benzonase treatment as previously described.

To monitor M.HpaII degradation $pDPC^{Lead}$ or $pDPC^{Lag}$ plasmids were pre-bound with purified LacI (untagged) for 60 min at RT as previously described (Duxin et al., 2014). Pre-bound plasmids were replicated at 5 ng/μL final concentration in HSS/NPE, and reactions stopped in DPC pull-down buffer. DPC plasmids were pulled down washed and benzonase treated as described above. After elution with benzonase, the eluates were incubated with His-tag dynabeads to recover M.HpaII-His_6 DPCs (Life Technologies) in HIS wash buffer (50 mM sodium phosphate buffer, pH8, 150 mM NaCl, 0.02% Tween-20) for 10 minutes at 4°C. This step was added to avoid cross reactivity between free LacI and the M.HpaII antibody. Beads were washed three times in HIS wash buffer and eluted in HIS elution buffer (300 mM imidazole, 50 mM sodium phosphate buffer, pH8, 300 mM NaCl, 0.01% Tween20) shaking at RT for 5 min. The supernatant M.HpaII-His_6 eluate was mixed with 2X Laemmli sample buffer for subsequent western blotting analysis.

Plasmid Pull-down Mass Spectrometry (PP-MS)

Plasmid DNA was replicated in egg extracts at 5 ng/μL (final concentration). At the indicated time points, 8 μL of the reaction were withdrawn and plasmids and associated proteins were recovered by plasmid pull down using LacI coated beads (Budzowska et al., 2015). After 30 min incubation at 4°C, samples were washed twice in 10 mM HEPES pH 7.7, 50 mM KCl, 2.5 mM $MgCl_2$, 0.03% Tween 20, and once in 10 mM HEPES pH 7.7, 50 mM KCl, 2.5 mM $MgCl_2$. Samples were washed one additional time in 50 μL of 10 mM HEPES pH 7.7, 50 mM KCl, 2.5 mM $MgCl_2$ and transferred to a new tube to remove residual detergent. Beads were dried out and resuspended in 50 μL denaturation buffer (8 M Urea, 100 mM Tris pH 8.0). Cysteines were reduced (1 mM DTT, 15 minutes at RT) and alkylated (5 mM iodoacetamide, 45 min at RT). Proteins were digested and eluted from beads with 1.5 μg LysC (Sigma) for 2.5 hr at RT. Eluted samples were transferred to a new tube and diluted 1:4 with ABC (50 mM ammonium bicarbonate). 2.5 μg trypsin was added and incubated for 16 hours at 30°C. NaCl was added to 400 mM final concentration, and peptides were acidified and purified by stage tipping on C18 material. Samples were analyzed on a Q Exactive HF Orbitrap mass spectrometer (Thermo Scientific) and quantified by the label free algorithm implemented in the MaxQuant software, as previously described (Räschle et al., 2015). MS experiments were carried out in quadruplicates. A fifth replicate was used to isolate the DNA repair intermediates shown in Figure 2A. The mass spectrometry data have been deposited to the ProteomeXchange repository with the dataset identifier PXD008831.

QUANTIFICATION AND STATISTICAL ANALYSIS

All bioinformatics analysis was carried out with the Perseus software Version 1.5.6.0. For each comparison, the processed data was filtered to contain at least 3 valid values in at least one of the replicate group. A modified, one-sided T-Test implemented in Perseus (Tyanova et al., 2016) was carried out using a False Discovery Rate (FDR) cut-off of 0.01 and S0 = 4. Autoradiographs were quantified using ImageJ. Error bars represent standard deviations.

DATA AND SOFTWARE AVAILABILITY

Perseus is provided by the group of Jürgen Cox at the MPI of Biochemistry and can be freely downloaded at: http://www.coxdocs.org.

Molecular Cell, Volume *73*

Supplemental Information

Replication-Coupled DNA-Protein Crosslink Repair by SPRTN and the Proteasome in *Xenopus* Egg Extracts

Nicolai B. Larsen, Alan O. Gao, Justin L. Sparks, Irene Gallina, R. Alex Wu, Matthias Mann, Markus Räschle, Johannes C. Walter, and Julien P. Duxin

Supplemental Figure Legends

Figure S1. Replication-coupled DPC ubiquitylation and replication-independent DPC SUMOylation. Related to Figure 1. (**A**) Schematic illustrating replication intermediates generated during replication of pDPC (Duxin et al., 2014). (**B**) pDPC was replicated in egg extracts in the presence of [α-^{32}P]dATP. Geminin (+Gem.) was supplemented where indicated to block DNA replication (Tada et al., 2001; Wohlschlegel et al., 2000). Samples were analyzed by agarose gel electrophoresis. RI, replication intermediates; OC, open circular; SC, supercoiled. (**C**) pDPC^{2xLead} was incubated in egg extracts that do not support CMG licensing and replication initiation. Recombinant UBC9 dominant-negative (+dnUBC9) was added where indicated to block SUMOylation (Azuma et al., 2003). DPCs were recovered and monitored as in Figure 1B. * indicates residual uncrosslinked M.HpaII. (**D**) pDPC^{2xLead} was replicated in egg extracts supplemented with buffer (+buf) or UBC9 dominant-negative (+dnUBC9). DPCs were recovered and monitored as in Figure 1B. (**E**) pDPC^{2xLead} was replicated in egg extracts. At 15 minutes, when most DPCs are polyubiquitylated, DPCs were recovered and subsequently treated with the indicated deubiquitinating enzyme (DUB) (Hospenthal et al. 2015). DPCs were monitored as in Figure 1B with a M.HpaII antibody. A red dashed line was added to compare the length of ubiquitin chains between the different treatments. (**F**) pDPC^{2xLead} was replicated and DPCs monitored as in (E). DPCs were treated with the indicated combinations of DUBs prior to electrophoresis. (**G**) pDPC^{2xLead} was replicated in egg extracts in the presence of buffer (+buf), 15 μM of ubiquitin-vinyl-sulfone (UbVS), or 15 μM of UbVS and 80 μM of recombinant ubiquitin. DPCs were recovered and monitored as in Figure 1B.

Figure S2. Validation of PP-MS. (**A**) Analysis of protein recruitment to pCTRL at 10 min compared to pCTRL at 40 min (x axis) and pCTRL at 10 min compared to pCTRL + Geminin at 10 min (y axis). The plot shows the mean difference of the protein intensity of 4 biochemical replicates for each of the conditions indicated. (**B**) Analysis of protein recruitment to pCTRL compared to pCTRL + Geminin at 10 min. The volcano plot shows the mean difference of the protein intensity plotted against the *P* value of 4 biochemical replicates. (**C**) Samples from Figure 2A (autoradiograph) were also digested with FspI and AatII and nascent strand separated on a denaturing polyacrylamide gel. The schematic on top depicts the expected species of nascent leading strands and extension products liberated by FspI and AatII digestion of pDPC^{2xLead} replication intermediates (green hexamer, CMG helicase; red lines, nascent DNA). The locations of the corresponding bands on the gel are indicated by brackets. (**D**) pDPC^{2xLead} and pCTRL were replicated in egg extracts and

recovered at the indicated time point as depicted in Figure 2A (Budzowska et al., 2015). Samples were blotted with the indicated antibodies. Note that this experiment was performed with a different extract exhibiting faster replication kinetics than the one used for MS analysis. (**E**) Analysis of protein recruitment to pDPC^{2xLead} at 40 min compared to pCTRL at 10 min (x axis) and pDPC^{2xLead} at 40 min compared to pDPC^{2xLead} + Geminin at 12 min (y axis). The plot shows the mean difference of the protein intensity of 4 biochemical replicates for each of the conditions indicated.

Figure S3. SPRTN and the proteasome degrade DPCs. Related to Figure 3. (**A**) Mock-depleted and SPRTN-depleted egg extracts were used to replicate pCTRL in the presence of [α-^{32}P]dATP. 200 *μ*M MG262 was added where indicated. Samples were analyzed by native agarose gel electrophoresis as in Figure 3B. The quantification of a representative biological replicate is shown. (**B**) and (**C**) Biological replicates of Figure 3C. (**D**) Mock-depleted, PSMA1-depleted, SPRTN-depleted or PSMA1-SPRTN-depleted extracts were blotted against SPRTN and two proteasome subunits (PSMA1 and PSMA3). ORC2 was blotted as loading control. (**E**) Extracts from (D) were used to replicate pDPC^{2xLead}. DPCs were recovered and monitored as in Figure 1B.

Figure S4. SPRTN-mediated DPC proteolysis facilitates TLS. Related to Figure 4. (**A**) pme-DPC^{2xLead} was replicated in egg extracts supplemented with either DMSO (Mock) or 200 *μ*M of MG262 and analyzed as in Figure 3B. The quantification of a representative biological replicate is shown. (**B**) Samples from (A) were used to monitor DPC degradation as in Figure 1B using anti-M.HpaII antibody. Two different exposures are shown as indicated. (**C**) Schematic of human and *Xenopus laevis* SPRTN protein. Note that *Xenopus laevis* SPRTN contains a duplication of the C-terminal UBZ domain. (**D**) Alignment of the different functional motifs of human and *Xenopus laevis* SPRTN. Residues mutated to generate EQ, SHP*, PIP* and UBZ* are indicated. (**E**) Mock-depleted and SPRTN-depleted extracts were blotted against SPRTN or MCM6 (loading control). SPRTN-depleted extracts were supplemented with either buffer (+buf), or the indicated recombinant FLAG-SPRTN variants. (**F**) Extracts from (D) were used to replicate pme-DPC^{2xLead} in the presence of [α-^{32}P]dATP. Samples were analyzed by agarose gel electrophoresis as in Figure 3B and quantified for open circular (OC) and supercoiled (SC) products. The quantification of a representative biological replicate is shown. (**G**) Mock-depleted and SPRTN-depleted extracts were used to replicate pme-DPC^{2xLead}. SPRTN-depleted extracts were supplemented with either buffer (+buf), or recombinant FLAG-SPRTN variants, as indicated. Samples were analyzed by agarose gel electrophoresis as in Figure 3B and quantified for open circular and supercoiled products. The quantification of a representative biological replicate is shown. (**H**) Samples from (G) were used to monitor SPRTN-

mediated DPC degradation as in Figure 4E. (**I**) Nascent strand intermediates stall at -1, 0 and +1 and not at 0, +1 and +2 as previously reported (Duxin et al., 2014). 30 min samples of pDPC were analyzed by denaturing polyacrylamide gel electrophoresis following nb.BsmI digest alongside a reference oligo and a sequencing ladder generated with ddCTP and ddGTP. In lane 3, the 30 min sample of pDPC was premixed with the reference oligo before electrophoresis. In lanes 5 and 7, the reference oligo was premixed with the cytosine (ddCTP) and guanine (ddGTP) samples of the sequencing ladder, respectively. Note that the middle band of the three discrete bands generated during replication of pDPC aligns with the reference oligo (0 position). Note also that the sequencing ladder alignment with the reference oligo is shifted 1 nucleotide (lanes 4-8). This is likely caused by a faster migration of the sequencing ladder induced by the terminal dideoxynucleotide. In Duxin et al. 2014, the nascent strand stalling positions were determined by comparing pDPC to a sequencing ladder generated by dideoxy sequencing causing the incorrect assignment of the stalling intermediates. (**J**) Mock-depleted and SPRTN-depleted egg extracts were used to replicate pDPC in the presence of [α-^{32}P]dATP. SPRTN-depleted extracts were supplemented with either buffer (+buf), recombinant FLAG-SPRTN (+WT), or recombinant catalytically inactive FLAG-SPRTN E89Q (+EQ). Samples were analyzed by native agarose gel electrophoresis as in Figure 3B. Red arrowheads indicate accumulation of OC repair intermediates. Error bars represent standard deviation of 3 independent experiments. (**K**) Samples from (I) were digested with FspI and AatII and separated on a denaturing polyacrylamide gel and quantified as in Figure 4G. The quantification of a representative biological replicate is shown. (**L**) Depiction of pDPC^{PK} generation. (**M**) Mock-depleted and SPRTN-depleted egg extracts were used to replicate pDPC and pDPC^{PK} in the presence of [α-^{32}P]dATP. Samples were digested with FspI and AatII and separated on a denaturing polyacrylamide gel and quantified as in Figure 4G. The quantification of a representative biological replicate is shown. (**N**) Mock-depleted and REV1-depleted egg extracts were used to replicate pDPC and pDPC^{PK}. Samples were digested with NcoI and AatII and separated on a polyacrylamide denaturing gel as in Figure 4G. The quantification of a representative biological replicate is shown. Note that the plasmid used in this experiment contains the DPC on the bottom strand (pDPC^{Bot} in (Duxin et al., 2014)).

Figure S5. DPC ubiquitylation and degradation can occur in the absence of the replisome. Related to Figure 5. (**A**) and (**B**) Biological replicates of Figure 5C. (**C**) pDPC^{ssDNA} was incubated in mock-depleted, PSMA1-depleted, SPRTN-depleted or PSMA1-SPRTN-depleted non-licensing extracts. DPCs were recovered and monitored with a M.HpaII antibody as in Figure 1B. * indicates residual uncrosslinked M.HpaII. (**D**) Schematic representation of gap filling synthesis when

pDPC^{ssDNA} is incubated in non-licensing extracts (non-replicating). (**E**) pDPC or pDPC^{ssDNA} were incubated in non-licensing egg extracts in the presence of [α-^{32}P]dATP. Samples were analyzed by agarose gel electrophoresis as in Figure 3B. (**F**) Samples from (E) were digested with PvuII and NdeI and separated on a denaturing polyacrylamide gel (bottom autoradiograph). The different extension products are depicted in the upper scheme. (**G**) pDPC^{ssDNA} or pme-DPC^{ssDNA} were incubated in non-licensing egg extracts. DPCs were recovered and monitored as in Figure 1B. Samples at time 0 were withdrawn prior to incubating plasmids in egg extracts and thus lack input signal.

Figure S6. SPRTN-, but not proteasome-dependent DPC degradation requires nascent strand extension to the lesion. Related to Figure 6. (**A**) Depiction of aphidicolin and AraCTP addition after 3 min of incubation of pme-DPC^{ssDNA} in non-licensing extracts. (**B**) pme-DPC^{ssDNA} was incubated in non-licensing extracts. At 3 min extracts were supplemented with 700 μM aphidicolin and 1 mM araCTP where indicated. Samples were analyzed by agarose gel electrophoresis as in Figure 3B. (**C**) Samples from (E) were digested with PvuII and NdeI and separated on a denaturing polyacrylamide gel (bottom autoradiograph). The different extension products are depicted in the upper scheme. (**D**) DPCs from (B) were recovered and monitored as in Figure 1B. (**E**) Depiction of pDPC$^{+peptide}$ replication in REV1-depleted extracts. (**F**) pDPC$^{+peptide}$ was replicated in REV1 depleted extracts supplemented with either DMSO or 200 μM MG262 where indicated. DNA was digested with nb.BsmI and nascent leading strands were then separated on a polyacrylamide denaturing gel as in Figure 6F. (**G**) Samples from (F) were used to monitor DPC degradation as in Figure 1B. * indicates residual uncrosslinked M.HpaII.

Figure S7. TRAIP ubiquitin ligase stimulates replication-coupled DPC ubiquitylation. Related to Figure 7. (**A**) pDPC^{2xLead} was replicated in SPRTN-depleted extracts that were also either Mock-depleted or TRAIP-depleted. DPCs were recovered and monitored as in Figure 1B. (**B**) Biological replicate of experiment in (A). (**C**) Extracts described in (A) were used to replicate pDPC^{2xLead} in the presence of [α-^{32}P]dATP. Samples were analyzed by native agarose gel electrophoresis as in Figure 3B. Error bars represent the standard deviation of 3 independent experiments. (**D**) pDPC^{ssDNA} was incubated in non-licensing extracts that were SPRTN-depleted and either Mock-depleted or TRAIP-depleted. DPCs were recovered and monitored as in Figure 1B. * indicates residual uncrosslinked M.HpaII. (**E**) pDPC^{2xLead} was replicated in SPRTN-depleted extracts that were either Mock-depleted,

RTEL1-depleted, TRAIP-depleted, or RTEL1 and TRAIP co-depleted. DPCs were recovered and monitored as in Figure 1B. (**F**) Samples from Figure 7D were replicated in the presence of [α-^{32}P]dATP. Samples were analyzed by native agarose gel electrophoresis as in Figure 3B. The quantification of a representative biological replicate is shown. (**G**) pme-DPC^{2xLead} was replicated in Mock or TRAIP-depleted extracts in the presence of [α-^{32}P]dATP and nascent strand intermediates were analyzed as in Figure 4G. Error bars represent the standard deviation of 3 independent experiments.

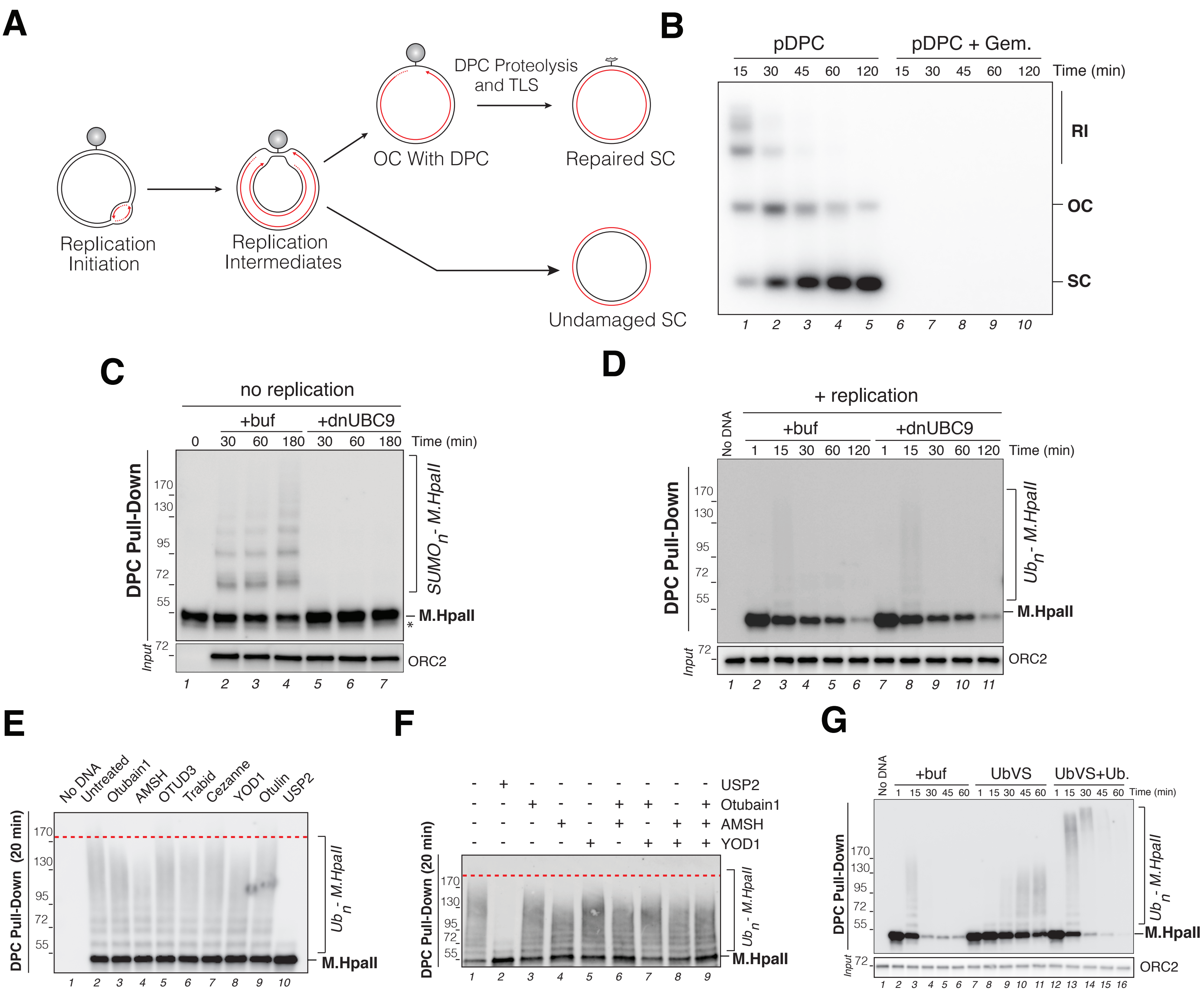
A
Replication Initiation
Replication Intermediates
OC With DPC
DPC Proteolysis and TLS
Repaired SC
Undamaged SC
B
pDPC
pDPC + Gem.
15 30 45 60 120 15 30 45 60 120 Time (min)
RI
OC
SC
1 2 3 4 5 6 7 8 9 10
C
no replication
+buf
+dnUBC9
0 30 60 180 30 60 180 Time (min)
DPC Pull-Down
170
130
95
72
55
SUMOn- M.HpaII
M.HpaII
*
Input
72
ORC2
1 2 3 4 5 6 7
D
+ replication
No DNA
+buf
+dnUBC9
1 15 30 60 120 1 15 30 60 120 Time (min)
DPC Pull-Down
170
130
95
72
55
Ubn- M.HpaII
M.HpaII
Input
72
ORC2
1 2 3 4 5 6 7 8 9 10 11
E
No DNA
Untreated
Otubain1
AMSH
OTUD3
Trabid
Cezanne
YOD1
Otulin
USP2
DPC Pull-Down (20 min)
170
130
95
72
55
Ubn - M.HpaII
M.HpaII
1 2 3 4 5 6 7 8 9 10
F
- + - - - - - - - USP2
- - + - - + + - + Otubain1
- - - + - + - + + AMSH
- - - - + - + + + YOD1
DPC Pull-Down (20 min)
170
130
95
72
55
Ubn - M.HpaII
M.HpaII
1 2 3 4 5 6 7 8 9
G
No DNA
+buf
UbVS
UbVS+Ub.
1 15 30 45 60 1 15 30 45 60 1 15 30 45 60 Time (min)
DPC Pull-Down
170
130
95
72
55
Ubn - M.HpaII
M.HpaII
Input
72
ORC2
1 2 3 4 5 6 7 8 9 10 11 12 13 14 15 16

Figure S2

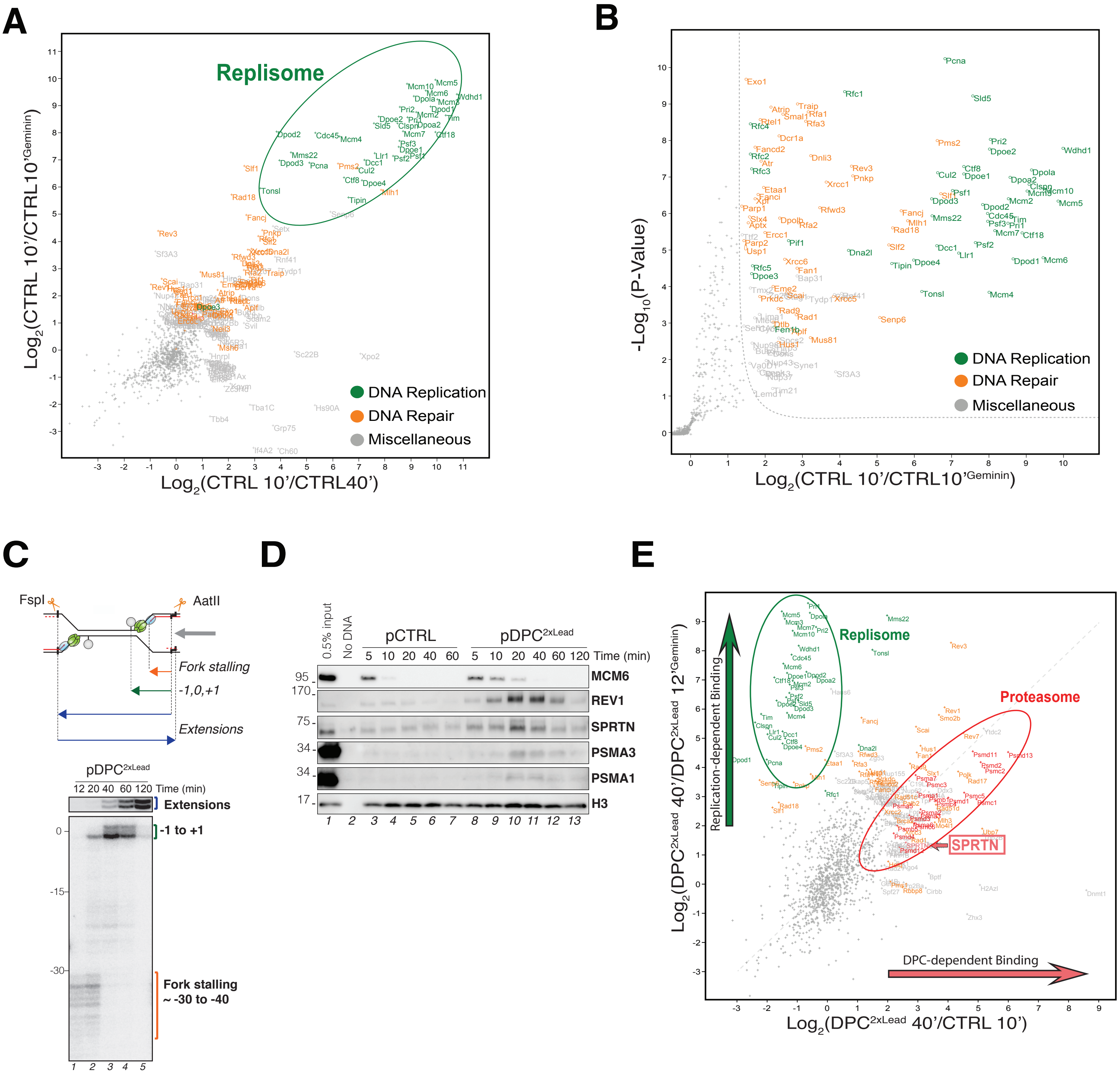

A
Replisome
Log2(CTRL 10'/CTRL10'Geminin)
Log2(CTRL 10'/CTRL40')
DNA Replication
DNA Repair
Miscellaneous
B
-Log10(P-Value)
Log2(CTRL 10'/CTRL10'Geminin)
DNA Replication
DNA Repair
Miscellaneous
C
FspI
AatII
Fork stalling
-1,0,+1
Extensions
pDPC2xLead
12 20 40 60 120 Time (min)
Extensions
-1 to +1
Fork stalling
~ -30 to -40
0
-15
-30
1 2 3 4 5
D
0.5% input
No DNA
pCTRL
pDPC2xLead
5 10 20 40 60 5 10 20 40 60 120 Time (min)
95
170
75
34
34
17
MCM6
REV1
SPRTN
PSMA3
PSMA1
H3
1 2 3 4 5 6 7 8 9 10 11 12 13
E
Replisome
Proteasome
SPRTN
Replication-dependent Binding
DPC-dependent Binding
Log2(DPC2xLead 40'/DPC2xLead 12'Geminin)
Log2(DPC2xLead 40'/CTRL 10')

Figure S3

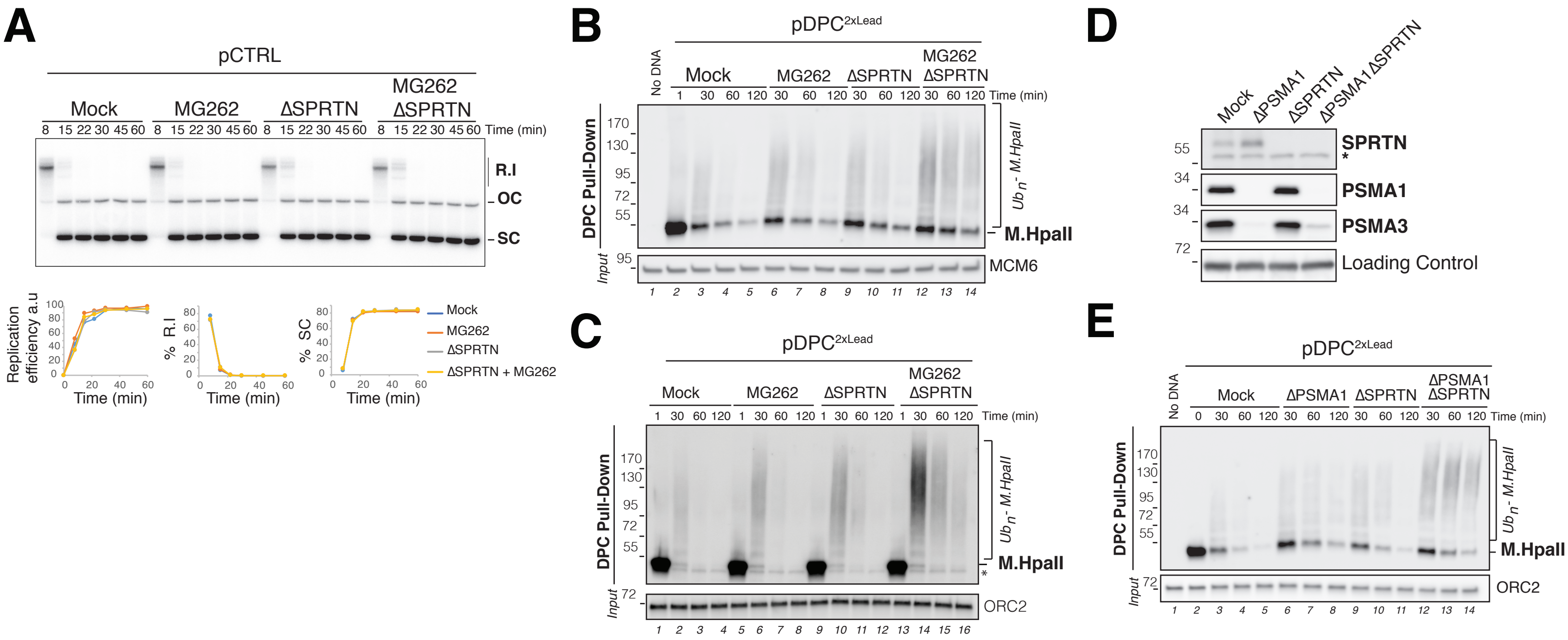

A
pCTRL
Mock
MG262
ΔSPRTN
MG262 ΔSPRTN
8 15 22 30 45 60
Time (min)
R.I
OC
SC
Replication efficiency a.u
% R.I
% SC
Time (min)
Mock
MG262
ΔSPRTN
ΔSPRTN + MG262
B
pDPC2xLead
No DNA
Time (min)
DPC Pull-Down
Input
Ubn- M.HpaII
M.HpaII
MCM6
C
pDPC2xLead
ORC2
D
Mock
ΔPSMA1
ΔSPRTN
ΔPSMA1ΔSPRTN
SPRTN
PSMA1
PSMA3
Loading Control
E
pDPC2xLead
ΔPSMA1 ΔSPRTN

Figure S4

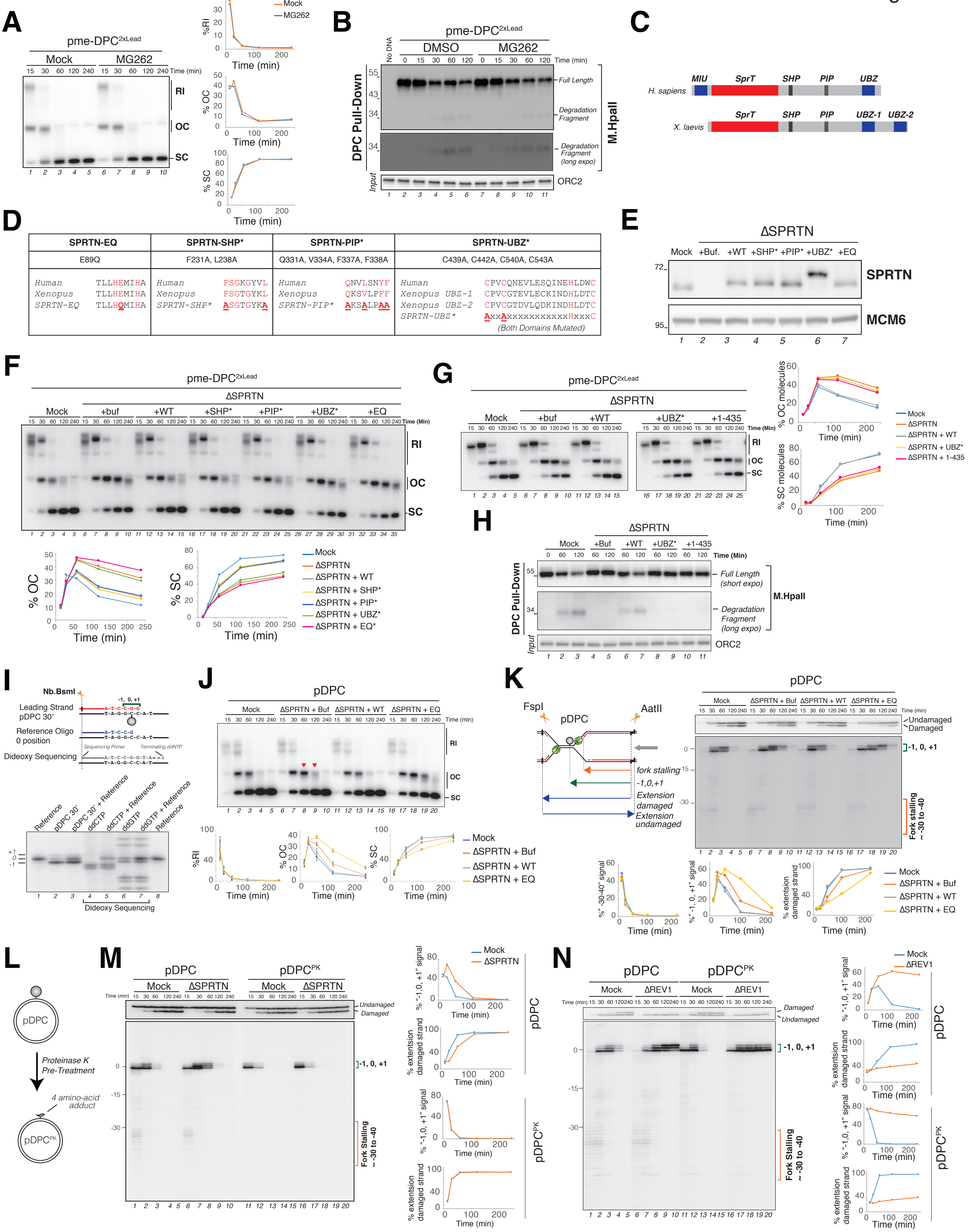
A
pme-DPC2xLead
Mock
MG262
Time (min)
RI
OC
SC
%RI
% OC
% SC
Time (min)
B
pme-DPC2xLead
No DNA
DMSO
MG262
DPC Pull-Down
Full Length
Degradation Fragment
Degradation Fragment (long expo)
M.HpaII
Input
ORC2
C
H. sapiens
X. laevis
MIU
SprT
SHP
PIP
UBZ
UBZ-1
UBZ-2
D
SPRTN-EQ
SPRTN-SHP*
SPRTN-PIP*
SPRTN-UBZ*
E89Q
F231A, L238A
Q331A, V334A, F337A, F338A
C439A, C442A, C540A, C543A
Human TLLHEMIHA
Xenopus TLLHEMIHA
SPRTN-EQ TLLHQMIHA
Human FSGKGYVL
Xenopus FSGTGYKL
SPRTN-SHP* ASGTGYKA
Human QNVLSNYF
Xenopus QKSVLPFF
SPRTN-PIP* AKSALPAA
Human CPVCQNEVLESQINEHLDWC
Xenopus UBZ-1 CPVCGTEVLECKINDHLDTC
Xenopus UBZ-2 CPVCGTDVLQDKINDHLDTC
SPRTN-UBZ* AxxAxxxxxxxxxxxHxxxC
(Both Domains Mutated)
E
ΔSPRTN
Mock +Buf. +WT +SHP* +PIP* +UBZ* +EQ
SPRTN
MCM6
F
pme-DPC2xLead
ΔSPRTN
Mock +buf +WT +SHP* +PIP* +UBZ* +EQ
Time (Min)
RI
OC
SC
% OC
% SC
Time (min)
Mock
ΔSPRTN
ΔSPRTN + WT
ΔSPRTN + SHP*
ΔSPRTN + PIP*
ΔSPRTN + UBZ*
ΔSPRTN + EQ*
G
pme-DPC2xLead
ΔSPRTN
Mock +buf +WT +UBZ* +1-435
Time (Min)
RI
OC
SC
% OC molecules
% SC molecules
Time (min)
Mock
ΔSPRTN
ΔSPRTN + WT
ΔSPRTN + UBZ*
ΔSPRTN + 1-435
H
ΔSPRTN
Mock +Buf +WT +UBZ* +1-435
Time (Min)
DPC Pull-Down
Full Length (short expo)
Degradation Fragment (long expo)
M.HpaII
Input
ORC2
I
Nb.BsmI
Leading Strand pDPC 30'
Reference Oligo 0 position
Dideoxy Sequencing
Sequencing Primer
Terminating ddNTP
Reference
pDPC 30'
pDPC 30' + Reference
ddCTP
ddCTP + Reference
ddGTP
ddGTP + Reference
Reference
Dideoxy Sequencing
J
pDPC
Mock ΔSPRTN + Buf ΔSPRTN + WT ΔSPRTN + EQ
Time (min)
RI
OC
SC
%RI
% OC
% SC
Time (min)
Mock
ΔSPRTN + Buf
ΔSPRTN + WT
ΔSPRTN + EQ
K
FspI
pDPC
AatII
fork stalling
-1,0,+1
Extension damaged
Extension undamaged
pDPC
Mock ΔSPRTN + Buf ΔSPRTN + WT ΔSPRTN + EQ
Time (min)
Undamaged
Damaged
-1, 0, +1
Fork stalling ~ -30 to -40
% "-30-40" signal
% "-1, 0, +1" signal
% extension damaged strand
Time (min)
Mock
ΔSPRTN + Buf
ΔSPRTN + WT
ΔSPRTN + EQ
L
pDPC
Proteinase K Pre-Treatment
4 amino-acid adduct
pDPCPK
M
pDPC
pDPCPK
Mock ΔSPRTN Mock ΔSPRTN
Time (min)
Undamaged
Damaged
-1, 0, +1
Fork Stalling ~ -30 to -40
% "-1, 0, +1" signal
% extension damaged strand
Time (min)
Mock
ΔSPRTN
pDPC
pDPCPK
N
pDPC
pDPCPK
Mock ΔREV1 Mock ΔREV1
Time (min)
Damaged
Undamaged
-1, 0, +1
Fork Stalling ~ -30 to -40
% "-1, 0, +1" signal
% extension damaged strand
Time (min)
Mock
ΔREV1
pDPC
pDPCPK

Figure S5

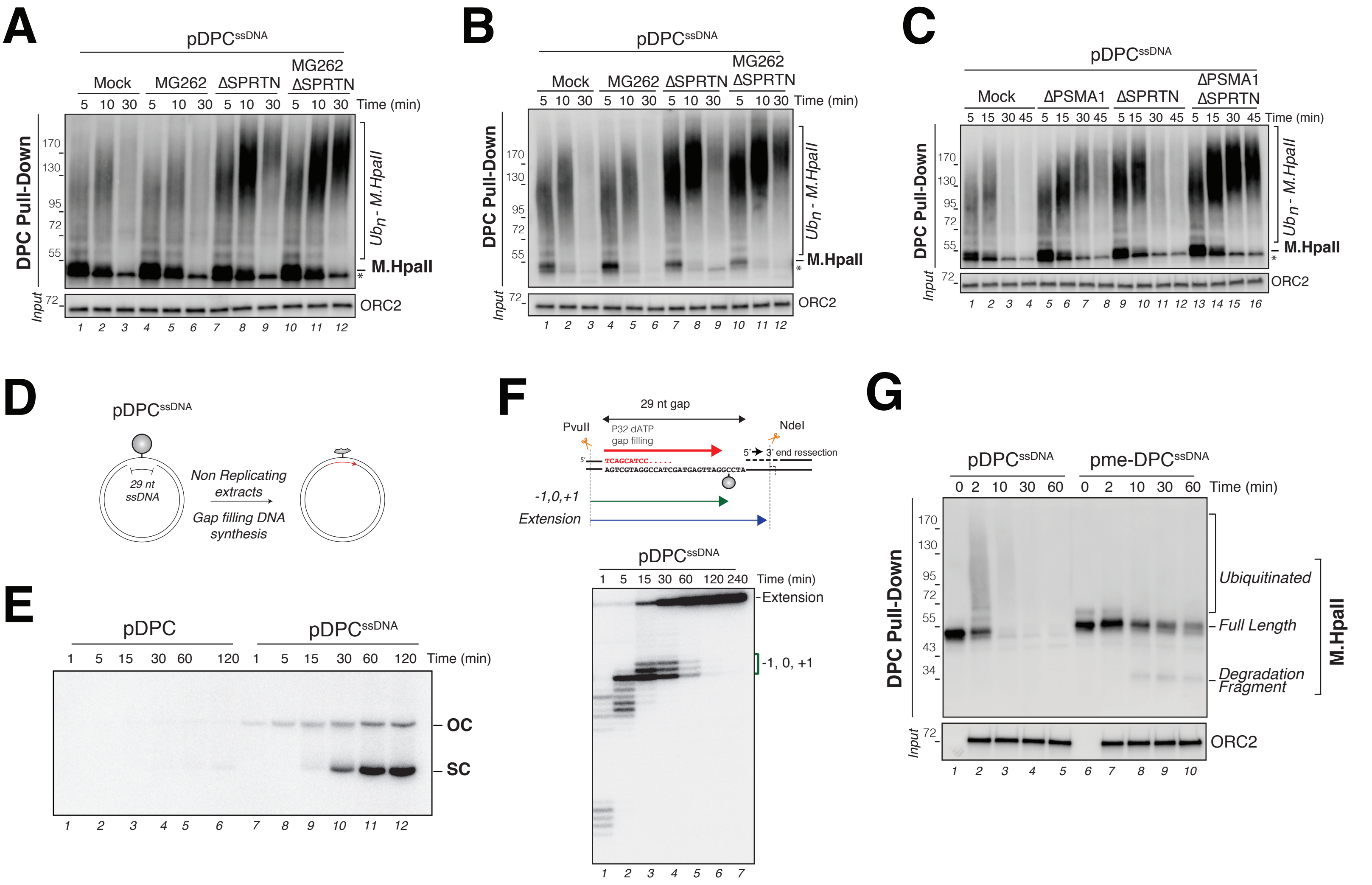

A
pDPC^ssDNA
Mock
MG262
ΔSPRTN
MG262 ΔSPRTN
5 10 30 5 10 30 5 10 30 5 10 30 Time (min)
DPC Pull-Down
Input
170
130
95
72
55
72
Ubn - M.HpaII
M.HpaII
*
ORC2
1 2 3 4 5 6 7 8 9 10 11 12
B
pDPC^ssDNA
Mock
MG262
ΔSPRTN
MG262 ΔSPRTN
5 10 30 5 10 30 5 10 30 5 10 30 Time (min)
DPC Pull-Down
Input
Ubn - M.HpaII
M.HpaII
ORC2
1 2 3 4 5 6 7 8 9 10 11 12
C
pDPC^ssDNA
Mock
ΔPSMA1
ΔSPRTN
ΔPSMA1 ΔSPRTN
5 15 30 45 5 15 30 45 5 15 30 45 5 15 30 45 Time (min)
DPC Pull-Down
Input
Ubn - M.HpaII
M.HpaII
ORC2
1 2 3 4 5 6 7 8 9 10 11 12 13 14 15 16
D
pDPC^ssDNA
29 nt ssDNA
Non Replicating extracts
Gap filling DNA synthesis
E
pDPC
pDPC^ssDNA
1 5 15 30 60 120 1 5 15 30 60 120 Time (min)
OC
SC
1 2 3 4 5 6 7 8 9 10 11 12
F
29 nt gap
PvuII
NdeI
P32 dATP gap filling
5' 3' end ressection
5'
TCAGCATCC.....
AGTCGTAGGCCATCGATGAGTTAGGCCTA
-1,0,+1
Extension
pDPC^ssDNA
1 5 15 30 60 120 240 Time (min)
Extension
-1, 0, +1
1 2 3 4 5 6 7
G
pDPC^ssDNA
pme-DPC^ssDNA
0 2 10 30 60 0 2 10 30 60 Time (min)
DPC Pull-Down
Input
170
130
95
72
55
43
34
72
Ubiquitinated
Full Length
Degradation Fragment
M.HpaII
ORC2
1 2 3 4 5 6 7 8 9 10

Figure S6

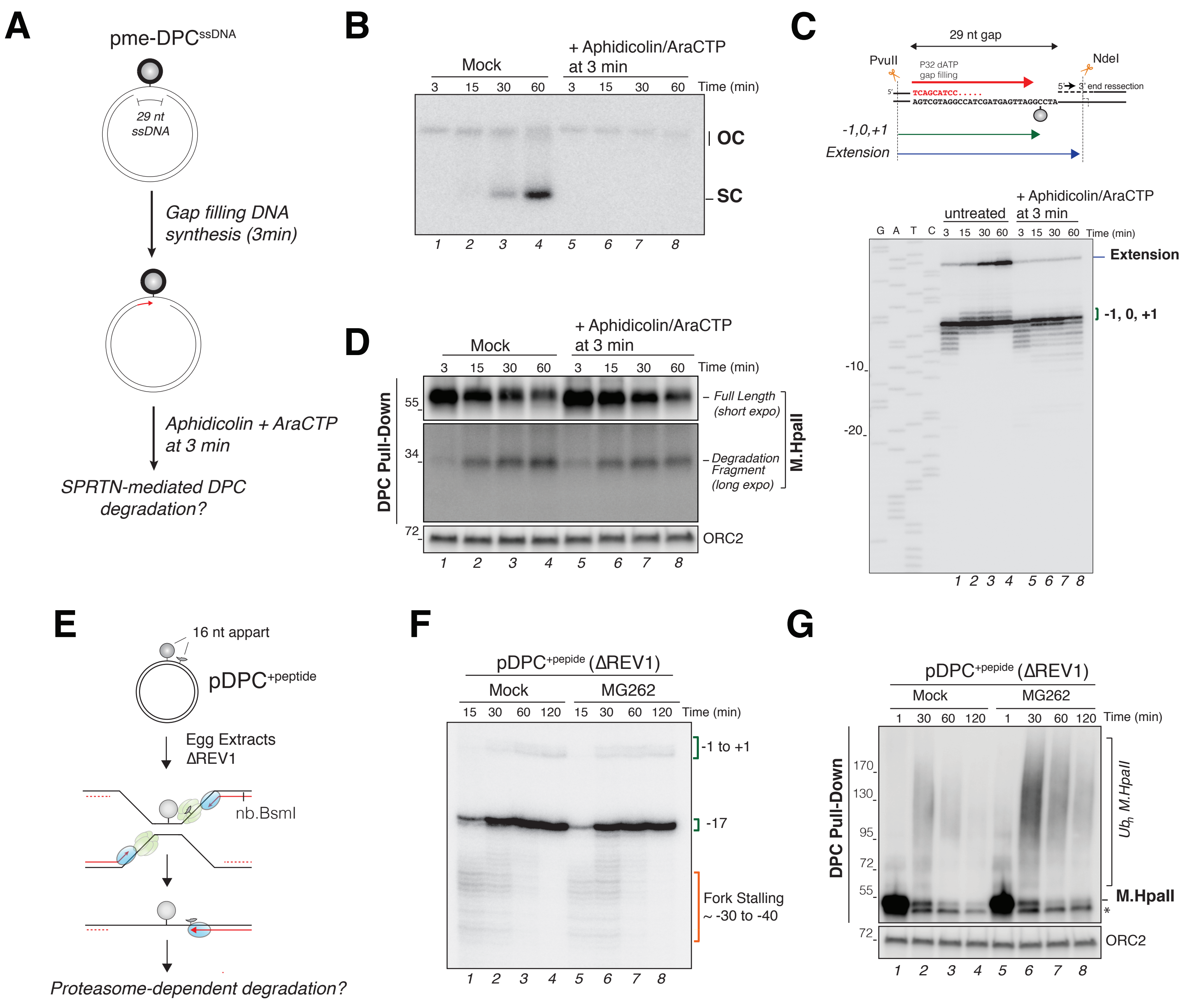

A
pme-DPC ssDNA
29 nt ssDNA
Gap filling DNA synthesis (3min)
Aphidicolin + AraCTP at 3 min
SPRTN-mediated DPC degradation?
B
Mock
+ Aphidicolin/AraCTP at 3 min
3 15 30 60 3 15 30 60 Time (min)
OC
SC
1 2 3 4 5 6 7 8
C
29 nt gap
PvuII
P32 dATP gap filling
NdeI
5' end ressection
TCAGCATCC.....
AGTCGTAGGCCATCGATGAGTTAGGCCTA
-1,0,+1
Extension
untreated
+ Aphidicolin/AraCTP at 3 min
G A T C 3 15 30 60 3 15 30 60 Time (min)
Extension
-1, 0, +1
-10
-20
1 2 3 4 5 6 7 8
D
Mock
+ Aphidicolin/AraCTP at 3 min
3 15 30 60 3 15 30 60 Time (min)
DPC Pull-Down
55
34
72
Full Length (short expo)
Degradation Fragment (long expo)
M.HpaII
ORC2
1 2 3 4 5 6 7 8
E
16 nt appart
pDPC +peptide
Egg Extracts ΔREV1
nb.BsmI
Proteasome-dependent degradation?
F
pDPC +pepide (ΔREV1)
Mock
MG262
15 30 60 120 15 30 60 120 Time (min)
-1 to +1
-17
Fork Stalling ~ -30 to -40
1 2 3 4 5 6 7 8
G
pDPC +pepide (ΔREV1)
Mock
MG262
1 30 60 120 1 30 60 120 Time (min)
DPC Pull-Down
170
130
95
72
55
Ubn M.HpaII
M.HpaII
*
72
ORC2
1 2 3 4 5 6 7 8

Figure S7

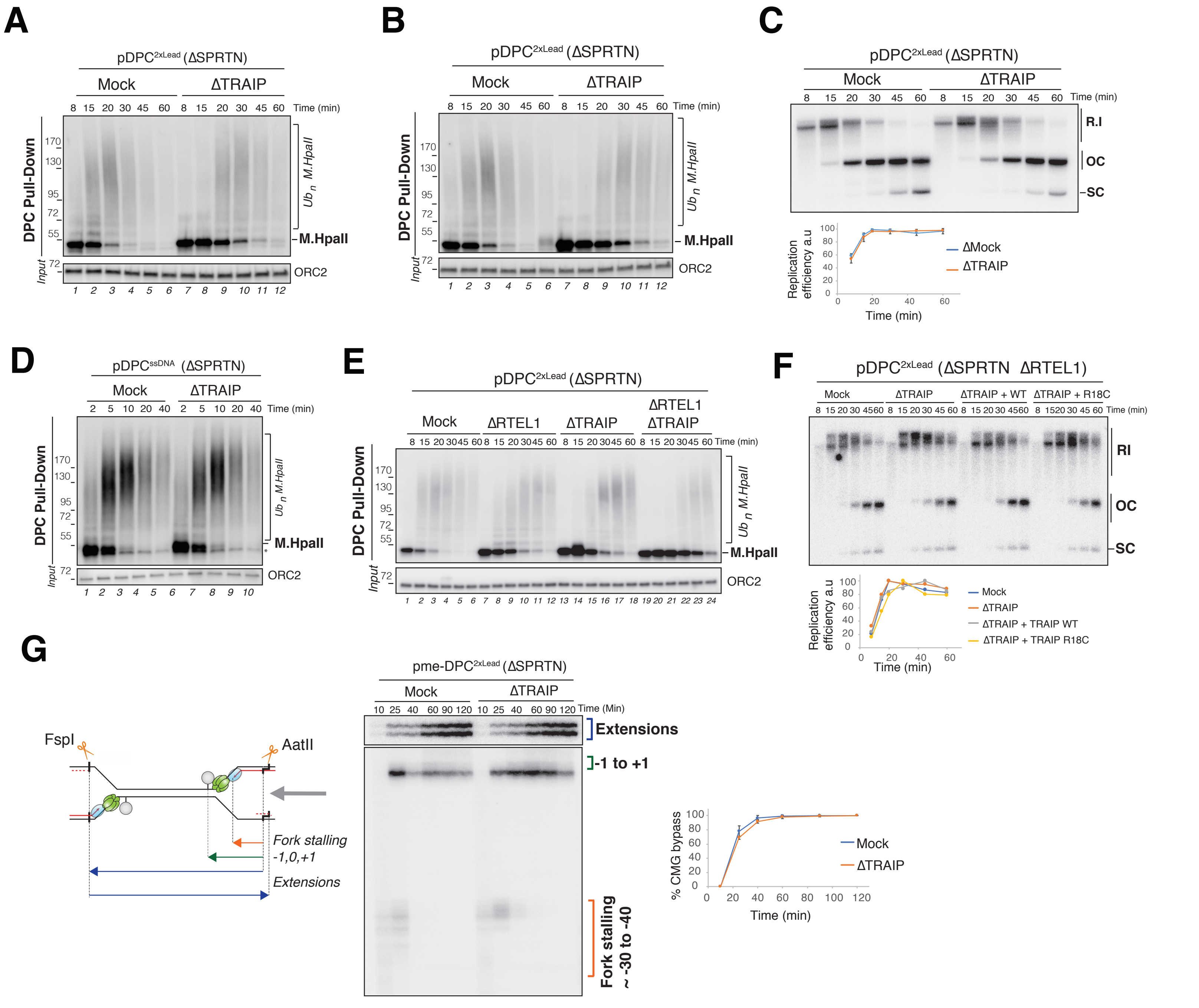
A
pDPC2xLead (ΔSPRTN)
Mock
ΔTRAIP
Time (min)
DPC Pull-Down
Input
Ub n M.HpaII
M.HpaII
ORC2
B
pDPC2xLead (ΔSPRTN)
Mock
ΔTRAIP
C
pDPC2xLead (ΔSPRTN)
R.I
OC
SC
Replication efficiency a.u
Time (min)
ΔMock
ΔTRAIP
D
pDPCssDNA (ΔSPRTN)
Mock
ΔTRAIP
E
pDPC2xLead (ΔSPRTN)
Mock
ΔRTEL1
ΔTRAIP
ΔRTEL1 ΔTRAIP
F
pDPC2xLead (ΔSPRTN ΔRTEL1)
Mock
ΔTRAIP
ΔTRAIP + WT
ΔTRAIP + R18C
RI
OC
SC
ΔTRAIP + TRAIP WT
ΔTRAIP + TRAIP R18C
G
FspI
AatII
Fork stalling
-1,0,+1
Extensions
pme-DPC2xLead (ΔSPRTN)
Time (Min)
Extensions
-1 to +1
Fork stalling ~ -30 to -40
% CMG bypass
Time (min)
Mock
ΔTRAIP
